# Dietary Risk Factors and Associated Disease Burden Among Chinese Adults Aged 25 Years and Older: Systematic Analysis of the Global Burden of Disease Study 2021

**DOI:** 10.2196/72978

**Published:** 2025-08-25

**Authors:** Yuze Xin, Dong Shui, Guangcan Yan, Wei Tian, Ning Tang, Jinyu Liang, Junyi Peng, Hongru Sun, Anqi Ge, Xinyan Liu, Katrina Kissock, Kathy Trieu, Jing Zhang, Xinyi Zhang, Pengpeng Ye, Maoyi Tian

**Affiliations:** 1 School of Public Health, Harbin Medical University, 157 Baojian Road, Nangang District, Harbin, 150081, China, 86 18701355688; 2 School of Basic Medicine, Harbin Medical University, Harbin, China; 3 The George Institute for Global Health, Faculty of Medicine and Health, University of New South Wales, New South Wales, Australia; 4 School of Population Health, Faculty of Medicine and Health, University of New South Wales, New South Wales, Australia; 5 National Center for Chronic and Non-communicable Disease Control and Prevention, Chinese Center for Disease Control and Prevention, Beijing, China; 6 Department of General Practice, The Second Affiliated Hospital of Harbin Medical University, Harbin, China

**Keywords:** global burden of disease, dietary risk factors, diet-related diseases, ultraprocessed food, China

## Abstract

**Background:**

With rapid economic development and lifestyle changes, diet-related diseases have become a major public health concern globally. China is experiencing significant dietary transitions. From 2001 to 2021, the intake of staple foods declined, while the consumption of animal-based foods and ultraprocessed foods increased significantly. But comprehensive assessments of major dietary risk factors and the long-term health impacts of shifting dietary patterns in China remain unclear.

**Objective:**

This study aims to assess the disease burden attributable to dietary risk factors in China using data from the Global Burden of Diseases, Injuries, and Risk Factors Study 2021 (GBD 2021), and to examine long-term trends over the past 3 decades. In addition, it provides an in-depth analysis of the 3 major diet-related disease categories in China: cardiovascular diseases, neoplasms, and diabetes and kidney diseases.

**Methods:**

We extracted data from GBD 2021, focusing on diet-related health outcomes in China across 33 provinces and regions. Measures included deaths, years of life lost (YLLs), years lived with disability (YLDs), and disability-adjusted life years (DALYs), stratified by age, sex, and region. Age-standardized rates (ASRs) were calculated, and temporal trends were analyzed using estimated annual percentage change (EAPC).

**Results:**

In 2021, dietary risk factors accounted for 1.70 million deaths and 38.39 million DALYs among Chinese adults aged 25 years and older. The leading contributors were high sodium intake, low fruit consumption, and low whole grain intake. Cardiovascular diseases were the largest contributors to diet-related DALYs. The burden was more pronounced in males than in females and highest among older adults aged 80 years and older. Substantial regional variation was observed, with the Northeastern and Western regions showing higher burden. From 1990 to 2021, overall disease burden due to dietary risks declined steadily, as reflected by decreasing ASR-DALYs (EAPC= -1.76), YLLs, and death rates. In contrast, YLDs showed a slight upward trend (EAPC=0.75), indicating a shift toward increased years lived with disability. In addition, the relative contributions of specific dietary risk factors changed considerably. Low vegetable intake, once ranked the third in 1990, dropped to the 12^th^ by 2021, while high red meat consumption rose from the 15^th^ to 7^th^ place. Although the ranking of high-sugar beverage consumption did not change, the ASR-DALYs rate increased significantly, with a percentage change of 689.14% between 1990 and 2021.

**Conclusions:**

In China, the burden of diet-related diseases remains substantial. While the overall age-standardized disease burden has declined, marked regional and demographic disparities persist. Certain dietary risks, such as high red meat and sugar-sweetened beverage consumption, are rising, and high sodium intake remains a serious concern. These trends highlight the urgent need for comprehensive, adaptable, and evidence-based nutrition policies to be implemented to address the evolving diet-related disease burden across diverse populations in China.

## Introduction

Noncommunicable diseases (NCDs) are the leading causes of death globally, accounting for approximately 74% of all deaths worldwide [1]. Among these, cardiovascular diseases, neoplasms, diabetes, and kidney diseases represent the most prominent contributors to both mortality and disability [2,3]. The role of dietary risks in the development of NCDs has been extensively documented through long-term prospective studies and randomized controlled trials [4]. Unhealthy dietary patterns, characterized by high intake of sodium, trans fats, sugar-sweetened beverages, and red or processed meats, as well as insufficient consumption of fruits, vegetables, fiber, and whole grains, have emerged as the most critical and modifiable risk factors for NCDs and premature death [5-7]. As dietary risk factors have imposed an increasing health and economic burden worldwide, improving diet quality has consequently become a central focus of global health efforts to reduce the burden of NCDs [8].

Socioeconomic transformations have driven substantial changes in the national dietary landscape over the past few decades in China [9,10]. For instance, between 2000 and 2021, the consumption of traditional staple foods declined by 36.9%, while the intake of animal-based products, such as meat, eggs, milk, and fish and seafood, increased by 44.7%, 39.9%, 406.4%, and 67.6%, respectively [11]. Concurrently, the expansion of the food industry has contributed to the widespread availability and growing consumption of ultraprocessed foods, which are often high in salt, sugar, and unhealthy fats [12,13]. Moreover, the transition has not occurred uniformly across the country. Significant regional disparities in dietary patterns persist due to variations in economic development, food availability, cultural preferences, and local agricultural practices [14]. These differences pose substantial challenges for developing and implementing unified national dietary guidelines and nutrition interventions that are both culturally appropriate and context-specific.

In response to growing concerns over population-level nutrition and diet-related health risks, China has implemented a series of comprehensive measures aimed at improving dietary quality nationwide [15]. One of the cornerstones of these efforts is the development and periodic revision of the Chinese Dietary Guidelines, which were first published in 1989 and subsequently updated in 1997, 2007, 2016 [16]. The most recent version (the 5th edition) was released in 2022 [17]. These guidelines provide recommendations for nutrient intake for the general population and specific population groups, such as pregnant women or breastfeeding mothers, infants, children, and adolescents [18,19]. Complementing these technical guidelines, the Chinese government launched the Healthy China Action Plan (2019‐2030), which identifies “healthy diet promotion” as one of its key strategic initiatives [20]. This plan underscores a multisectoral approach that calls for joint efforts from government bodies, health professionals, schools, and the food industry to improve dietary behaviors and create supportive environments for nutrition across all life stages [20-22]. However, despite these policy advancements and public health campaigns, significant challenges remain [23].

Analyzing the disease burden associated with dietary risk factors in China is important to inform the development of targeted and context-specific nutrition policies [24,25]. Although previous studies have explored the relationship between individual dietary risk factors and specific diseases, or between overall dietary patterns and single disease categories, such as high salt intake and cardiovascular disease, or the association between dietary patterns and kidney disease [26,27]. A comprehensive assessment of the cumulative burden of all major dietary risks and their long-term health consequences remains limited. This has resulted in a gap in understanding the broader and long-term impact of dietary risks on population health in China. To address this evidence gap, we used data from the Global Burden of Diseases, Injuries, and Risk Factors Study 2021 (GBD 2021) to conduct a systematic assessment of the disease burden attributable to dietary risk factors in China. This study also analyzed temporal trends over the past 3 decades and focused on the 3 leading disease categories contributing to diet-related disease burden in the country: cardiovascular diseases, neoplasms, and diabetes and kidney diseases [25,28,29].

## Methods

### Overview

Using data from the GBD 2021, the study provided estimates of disease burden related to dietary risks in China, by sex and age, between 1990 and 2021. A detailed description of the methods for estimating the disease burden attributable to risk factors has been published elsewhere [4]. We summarize the methods for estimating disease burden related to dietary risks below.

Briefly, this study analyzed data on dietary risk factors associated with all diseases among adults aged 25 years and older across 33 provinces in China from 1990 to 2021. These include all 31 mainland provinces, autonomous regions, municipalities, and two special administrative regions, Hong Kong and Macao (Taiwan Province was excluded due to data unavailability). Data were segmented by age, year, sex, geographical region, and different dietary risk factors [30]. Data were segmented by age, year, sex, geographical region, and different dietary risk factors.

### Data Source and Definitions

GBD 2021 consisted of the most recent epidemiological data and had improved standardized methodologies compared to previous GBD data [2]. In this study, data classification conformed to the GBD database classification standards, encompassing age, region, and disease categories. This study not only analyzed the all-cause disease burden caused by dietary risk factors but also separately analyzed the 3 major disease categories: cardiovascular diseases, neoplasms, and diabetes and kidney diseases. All 3 disease categories are classified as Level 2 causes within the GBD classification system [2].

Dietary risk factors were behavioral factors that were correlated with the increased or decreased risk of developing diseases [2]. The selection of dietary risk factors includes 15 specific dietary risks that meet GBD criteria for risk factor selection. These criteria considered the significance of the risk factors in contributing to disease burden, as well as the availability of adequate data to estimate exposure to these risks. The selection process was further guided by the strength and consistency of epidemiological evidence supporting a causal relationship between dietary factors and disease. The method used to evaluate the strength of this evidence for causality is detailed elsewhere [4] and summarized in Table S3 in Multimedia Appendix 1.

### Measurement of Disease Burden

GBD 2021 quantified the proportion of disease burden attributable to each dietary factor that could be prevented if exposure levels were maintained at the minimum risk level, defined as the theoretical minimum risk exposure level (TMREL) [31]. Assuming exposure levels for other risk factors remain constant, the population attributable fraction (PAF) for a given risk factor was estimated by comparing the TMREL with the exposure level in a specific population [32]. GBD 2021 applied a comparative risk assessment framework to calculate the disease burden attributable to major dietary risk factors.

### Statistical Analysis

This study used various epidemiological indicators, including the number of deaths, mortality rate, years of life lost (YLLs), years lived with disability (YLDs), and disability-adjusted life years (DALYs) to describe the disease burden attributable to dietary factors. When comparing populations across different regions or age groups over time, we used age-standardized rates (ASRs), calculated as a weighted average of age-specific rates. All measures were reported as unstandardized and ASRs (rate per 100,000 persons), including their 95% uncertainty intervals (UIs), representing the range within which the true value is expected to lie with 95% probability. To explore the trends of ASRs from 1990 to 2021, we calculated the estimated annual percentage change (EAPC) of ASRs based on the formula EAPC=100×(exp(β) – 1), where Y=(α + βX + ε)=ln (ASR), X=calendar y, and *ε*=the error term. This model is based on the assumption that a natural logarithmic scale will show a linear trend of ASR over a specified time. The ASR is considered to exhibit an increasing trend when EAPC and the lower bound of the UI are positive, whereas the ASR is considered to exhibit a decreasing trend when EAPC and the upper bound of the UI are negative. All data were analyzed using RStudio (Version 2024.04.0).

### Ethical Considerations

This study used data from the GBD study, which was approved by the institutional review board of the University of Washington School of Medicine. The original data collection obtained informed consent from study participants or was granted exemptions by the institutional review board. As this is a secondary analysis of existing data, no additional human participant research ethics review or informed consent was required. Study data were anonymized and deidentified to protect the privacy and confidentiality of study participants. Our study adhered to the GBD protocol and followed the Guidelines for Accurate and Transparent Health Estimates Reporting (GATHER) [33]. The GATHER checklist is provided in Table S1 in Multimedia Appendix 1.

## Results

### Overall Disease Burden Attributable to Dietary Risk Factors in 2021

In 2021, 1.70million deaths (95% UI 0.69-2.68) from all diseases were attributed to dietary risk factors among people aged 25 years and older in China. The YLLs due to dietary factors amounted to 34.18million person-years (95% UI 14.57-52.84). The YLDs were 4.21million person-years (95% UI 1.57-6.73), and the DALYs totaled 38.39million person-years (95% UI 16.21-58.61). YLLs accounted for 89% of DALYs. The ASR-YLDs were 204.17 per 100,000 persons (95% UI 75.67-326.07), and the ASR-DALYs were 1892.69 per 100,000 persons (95% UI 775.77-2904.23) (Table 1).

**Table 1.** Overall and cause-specific disease burden attributable to dietary risk factors in China, 1990‐2021: deaths, years of life lost, years lived with disability, disability-adjusted life years (95% uncertainty intervals), and estimated annual percentage change.

Burden of disease indicators	Absolute number, thousands		Age-standardized rate, per 100,000 people		EAPC^a^ of ASR^b^^c^
	1990	2021	1990	2021	1990‐2021
Deaths (95% UI)^d^					
All causes	1011.13 (518.27-1474.48)	1704.02 (687.92-2684.78)	152.13 (76.40-222.36)	90.37 (35.35-143.15)	−1.68 (-2.49 to −1.42)
Cardiovascular diseases	829.87 (471.40-1149.29)	1449.88 (594.79-2244.04)	128.62 (69.70-181.22)	77.76 (30.45-121.22)	−1.62 (−2.67 to −1.30)
Diabetes and kidney diseases	31.94 (16.00-48.16)	76.77 (32.01-121.77)	4.77 (2.41-7.06)	3.91 (1.65-6.18)	−0.64 (−1.22 to −0.43)
Neoplasms	147.02 (29.72-306.47)	176.53 (60.80-367.46)	18.44 (3.89-38.01)	8.66 (2.98-17.89)	−2.43 (−2.41 to −0.86)
YLLs^e^ (95% UI)					
All causes	25261.26 (12317.56-36672.75)	34186.08 (14575.66-52839.91)	3103.51 (1545.93-4508.14)	1688.52 (689.96-2601.5)	−1.96 (−2.60 to −1.77)
Cardiovascular diseases	20213.91 (11357.25-27664.76)	28353.16 (12271.3-42425.37)	2536.16 (1418.19-3489.77)	1409.55 (584.61-2114.00)	−1.89 (−2.86 to −1.62)
Diabetes and kidney diseases	793.65 (397.55-1209.75)	1581.38 (654.40-2528.97)	97.41 (49.20-146.73)	75.81 (31.53-120.59)	−0.81 (−1.44 to −0.63)
Neoplasms	4189.84 (855.34-8742.78)	4229.48 (1429.36-8797.48)	462.79 (95.46-964.05)	202.08 (68.33-417.31)	−2.67 (−2.70 to −1.08)
YLDs^f^ (95% UI)					
All causes	1440.14 (648.54-2200.15)	4207.73 (1567.38-6728.75)	161.77 (72.93-250.42)	204.17 (75.67-326.07)	0.75 (0.12-0.85)
Cardiovascular diseases	824.48 (425.83-1258.08)	1913 (786.69-3080.93)	94.68 (47.86-145.38)	90.03 (36.97-145.22)	−0.16 (−0.83 to 0.01)
Diabetes and kidney diseases	550.76 (125.63-981.24)	2109.35 (403.13-3857.85)	59.72 (14.66-105.88)	105.35 (20.53-193.47)	1.83 (1.09-1.94)
Neoplasms	58.94 (15.08-114.05)	177.77 (47.72-340.07)	6.70 (1.77-12.80)	8.41 (2.25-16.11)	0.74 (0.73-0.77)
DALYs^g^ (95% UI)					
All causes	26701.40 (13025.80-38381.93)	38393.81 (16213.07-58607.05)	3265.28 (1625.52-4693.91)	1892.69 (775.77-2904.23)	−1.76 (−2.39 to −1.55)
Cardiovascular diseases	21038.39 (11853.04-28797.03)	30266.17 (13228.42-45225.09)	2630.84 (1471.93-3614.80)	1499.58 (632.89-2247.22)	−1.81 (−2.72 to −1.53)
Diabetes and kidney diseases	1344.40 (546.84-2147.75)	3690.73 (1021.61-6162.48)	157.13 (66.38-247.18)	181.17 (50.05-301.66)	0.46 (−0.91 to 0.64)
Neoplasms	4248.78 (868.87-8847.49)	4407.25 (1479.11-9106.09)	469.49 (97.07-975.92)	210.49 (70.5-431.87)	−2.59 (−2.63 to −1.03)

a EAPC: estimated annual percentage change.

b ASR: age-standardized rate.

c EAPC of ASR: The EAPC of ASR to explore the trends of ASRs from 1990 to 2021.

d UI: uncertainty interval.

e YLL: year of life lost.

f YLD: year lived with disability.

g DALY: disability-adjusted life year.

### Regional Distribution of Disease Burden Attributable to Dietary Risk Factors

**Figure 1.**
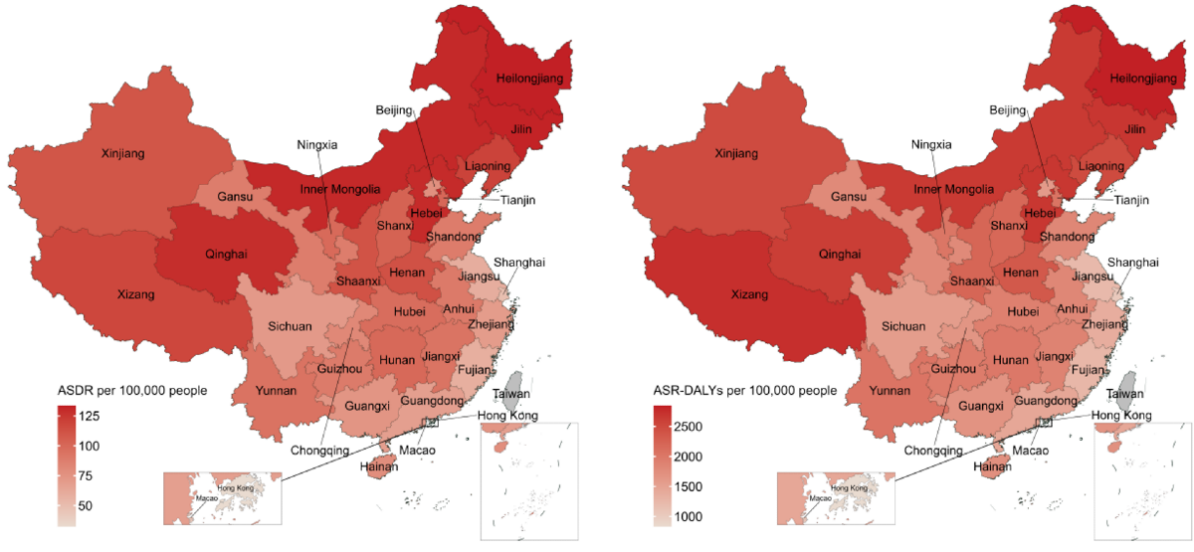
Regional distribution of age-standardized death rates and age-standardized disability-adjusted life year rates attributable to dietary risk factors in China, 2021. ASDR: age-standardized death rates; ASR-DALY: age-standardized disability-adjusted life year rates;

**Figure 2.**
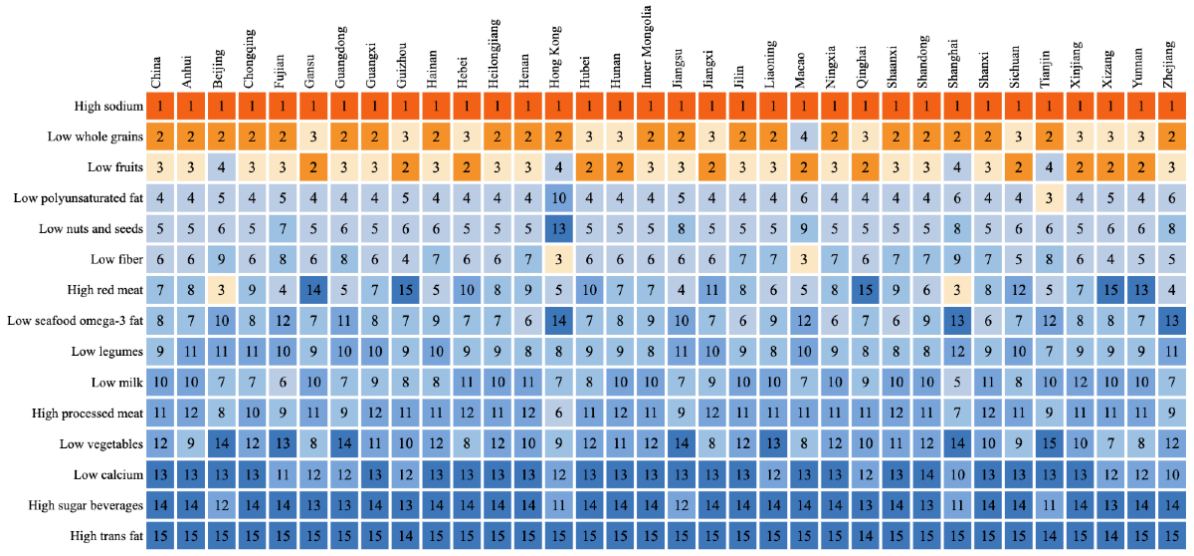
Provincial ranking of age-standardized disability-adjusted life year rates attributable to dietary risk factors in China, 2021.

There are significant regional differences in the disease burden attributed to dietary factors in China. Fujian, Shanghai, Macao, and Hong Kong experience a lower diet-related disease burden, while Hebei, Heilongjiang, Jilin, Inner Mongolia, and Qinghai have a higher diet-related disease burden. Figure 1 displays the distribution of age-standardized death rate (ASDR) and ASR-DALY attributed to dietary factors for all diseases. The contribution of individual dietary factors to the overall diet-related disease burden varies across regions. Figure 2 ranks the different dietary factors contributing to the disease burden in various regions of China, and the high-sodium diet emerges as the top risk factor for disease burden in all regions. A diet high in sodium and low in whole grains and fruit is the leading dietary risk factor in almost every region. In Shanghai and Beijing, high red meat consumption ranks third in contributing to the burden of diet-related diseases, while low fruit intake ranks fourth. In Macao and Hong Kong, low dietary fiber intake ranks third, with low whole-grain intake and low fruit intake ranking fourth, respectively.

### Dietary Risk Factors Associated With Individual Disease Burden in 2021

Cardiovascular diseases attributable to dietary risk factors were the leading cause of death and DALYs amongst disease groups, with 1.45million deaths (95% UI 0.59-2.24) and 30.26million person-years of DALYs (95% UI 13.23-45.23). The ASR-DALYs rate was 1499.58 per 100,000 persons (95% UI 632.89-2247.22). Meanwhile, diabetes and kidney diseases are attributed to dietary risk factors, with 0.08million deaths (95% UI 0.03-0.12) and 3.69million person-years of DALYs (95% UI 1.02-6.16). The ASR-DALYs rate was 181.17 per 100,000 persons (95% UI 50.05-301.66). Neoplasms attributed to dietary risk factors lead to 0.18million deaths (95% UI 0.06-0.37) and 4.41million person-years of DALYs (95% UI 1.47-9.11). The ASR-DALYs rate was 210.49 per 100,000 persons (95% UI 70.50,431.87).

### Sex and Age Distribution of Disease Burden Attributable to Dietary Risk Factors

The death rate for males due to dietary risk factors was 124.40 per 100,000 persons (95% UI 49.56-199.28), 1.89 times higher than females (65.73 per 100,000 persons, 95% UI 23.36-110.28). The YLDs rate for males was 225.21 per 100,000 persons (95% UI 86.13-355.83), and the DALYs rate was 2524.01 per 100,000 persons (95% UI 1034.57-3878.49). Whereas for females, the YLDs rate and DALYs rate were 183.97 per 100,000 persons (95% UI 64.49-299.26) and 1350.20 per 100,000 persons (95% UI 509.30-2179.77), respectively. The proportion of DALYs due to YLLs from dietary risk factors was 91.1% for males and 86.4% for females, with males having a slightly higher proportion compared to females. The disease burden attributable to dietary risk factors increases with age, particularly among individuals aged 60 years and older. Death rate, YLL rate, and DALY rate are the highest in the 80 years and older age group, while YLD rate was highest in the 75‐79 age group (1038.88 per 100,000 persons for the 75‐79 years age group compared with 964.02 per 100,000 persons for the 80 years and older age group). The proportion of YLLs to DALYs gradually increased with age, reaching 95.4% in the 80 years and older age group. Detailed data are presented in Tables S5-6 in Multimedia Appendix 1. The figures illustrating the trends are presented in Figures S1–5 in Multimedia Appendix 2.

### Trends of Disease Burden Attributed to Dietary Risk Factors From 1990 to 2021

**Figure 3.**
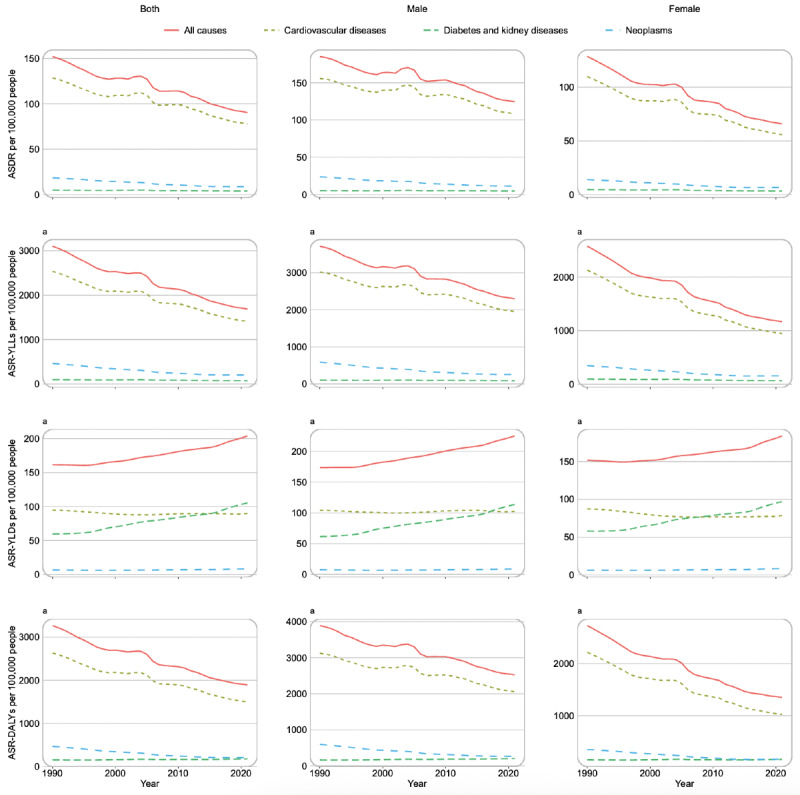
Temporal changes in disease burden (deaths, years of life lost, years lived with disability, and disability-adjusted life years) attributable to dietary risk factors in China, 1990‐2021. ASR-DALY: age-standardized rate-disability-adjusted life year; ASR-YLD: age-standardized rate-year lived with disability; ASR-YLL: age-standardized rate-year of life lost.

**Figure 4.**
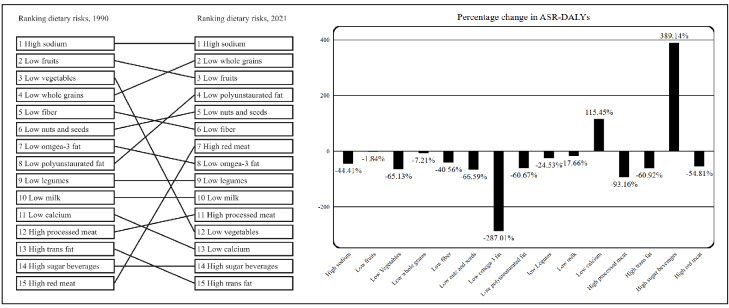
Changes in the ranking of dietary risk factors contributing to the proportion of disease burden (Age-standardized rates -disability-adjusted life years ) in China, 1990‐2021.

Figure 3 illustrates the temporal changes in the disease burden caused by dietary risk factors amongst Chinese adults aged 25 years and older from 1990 to 2021. The ASDR, ASR-YLLs, and ASR-DALYs for all diseases show a significant downward trend over time, with an EAPC of −1.76 (95% UI −2.39 to −1.55) for ASR-DALY. In contrast, ASR-YLDs exhibit an upward trend, with an EAPC of 0.75 (95% UI 0.12-0.85). Figure 4 illustrates the changes in the proportion of disease burden caused by different dietary risk factors. Over the past 30 years, there have been notable changes in dietary risk factors contributing to the diet-related disease burden. In 1990, low vegetable intake ranked third in its contribution but dropped to 12th place by 2021. In contrast, high red meat consumption rose from the lowest rank (15th) to seventh place. Although the ranking of sugar-sweetened beverages (SSBs) consumption as a dietary risk remained unchanged, the age-standardized DALY rate increased substantially, with a percentage change of 689.14% (95% UI 296.03%-512.16%) from 1990 to 2021.

## Discussion

### Principal Findings

In 2021, the dietary factors contributing to disease burden in China were more severe for males and older age groups. Cardiovascular diseases remain the primary contributors to the burden attributed to dietary risk factors. Key dietary contributors to disease burden include high sodium intake, low fruit consumption, and low whole-grain intake. The burden of disease, as measured by ASR-DALYs, is remarkably higher in certain areas of Northern and Western China compared with other regions.

The Chinese government has been striving to enhance the dietary health of its population [34], and since 1989, the Chinese Residents’ Dietary Guidelines have been issued and revised multiple times [20]. These initiatives have demonstrated effectiveness, as evidenced by improvements in disease burden over time. This progress is closely linked to China’s rapid economic expansion in recent years, resulting in greater dietary variety among the population and a relatively heightened consumption of specific food items, both healthful and unhealthful in nature [35,36]. One significant transformation is observed in vegetable consumption, which has transitioned in disease burden rankings from third place in 1990 to twelfth place. The discrepancy in the increasing trajectory of ASR-YLDs attributed to dietary factors, in contrast to other metrics, may be explained by advancements in medical treatment that prolong patient survival [37]. Furthermore, the rise in the prevalence of chronic diseases stemming from overnutrition could also play a role in this situation [38].

Although the overall disease burden from dietary factors has declined over time, the burden attributable to certain specific dietary risks, such as diets high in red meat and low in whole grains, has increased. In most provinces in China, especially in inland cities, red meat is still the main meat consumed [39,40], and economic development has also stimulated an increase in food variety, especially in meat consumption [41]. According to data from the China Statistical Yearbook, from 1990 to 2021, China’s red meat consumption showed a continuous upward trend except for a slight decline in 2019 [42,43]. In addition, the increased proportion of disease burden attributable to a diet low in whole grains may also be associated with economic development, which has led to a shift towards more refined grains in the diet. In economically underdeveloped periods, whole grains were more readily available than refined grains. As a result, higher whole-grain intake was often associated with increased poverty among the Chinese population [44]. This consciousness often results in reduced consumption of coarse grains and other whole-grain foods, exacerbating health risks [45].

Another change is the rise in the disease burden caused by a diet high in SSBs. The consumption of SSBs is rapidly increasing worldwide, especially among adolescents [46]. The rise in SSB intake is associated with various health issues, such as obesity, insulin resistance, and dental caries [47]. This has become a significant public health concern. In China, the intake of SSBs is also particularly concerning among young children, highlighting the need for early intervention [48]. To minimize the consumption of SSBs, the World Health Organization (WHO) released a manual in 2022 recommending taxation as an effective strategy to reduce SSB intake [49]. China may also adopt such measures in the future to curb SSB consumption [50]. The above findings suggest that as socioeconomic status improves, the nature of diet-related disease burden shifts. Instead of being driven by the lack of specific nutrients, it increasingly stems from imbalances in the overall dietary structure [51,52]. However, achieving this shift in dietary patterns is challenging for China, where economic growth is just beginning [53].

It is also noteworthy that from 1990 to 2021, high sodium intake and low fruit consumption consistently ranked among the leading dietary risk factors contributing to disease burden. Studies indicate that the average fruit intake among the Chinese population is around 64.30g per day [54], below both the recommendations of the Chinese Dietary Guidelines (daily intake for adults aged 18‐64 years is 200‐350 g of fruits) and those of the WHO (recommends a minimum combined daily intake of 400 g of fruits and vegetables) [17,55]. This can be attributed to factors such as low economic status, poor dietary literacy, and a lack of fruit consumption habits among residents [56]. Addressing these issues requires further efforts and plans at the national level, along with more frequent and in-depth health education initiatives. Educating individuals about healthy habits from a personal lifestyle perspective is relatively challenging and demands greater resourcing [57]. On the other hand, current sodium intakes of the Chinese population are high at around 4g per day [58], far exceeding the WHO maximum recommended level of <2g per day. Sodium intake in China typically comes from home cooking practices [59], which are deeply rooted in family cooking traditions and regional dietary cultures [60]. A more effective approach to reduce sodium is to promote the substitution of regular salt with potassium-enriched salt [61]. Addressing these factors also requires long-term health education efforts [60].

An analysis of population subgroups revealed variation in the disease burden attributable to dietary risk factors across different demographic characteristics. Our findings revealed notable differences in disease burden attributable to dietary risk factors by sex and age. Specifically, males experienced a substantially higher burden compared to females. This disparity may be partially explained by sex-specific differences in dietary behaviors, with men in China generally consuming higher amounts of salt, red and processed meats, and lower amounts of fruits and vegetables [62,63]. Thus, it potentially leads to a higher prevalence of diet-related NCDs among men [14]. In addition, we observed that the disease burden associated with dietary risks increases with age, particularly among individuals aged 60 years and older. This trend may reflect the cumulative effect of long-term exposure to unhealthy dietary habits, as well as increased physiological vulnerability and comorbidities in older adults [64].

The distribution of disease burden caused by dietary risk factors in China varies significantly between regions. According to the National Bureau of Statistics of China, the country is divided into Eastern, Central, Western, and Northeastern regions [65]. The death rate and DALYs are disproportionately higher in the Western and Northeastern regions, while the Eastern and Central regions exhibit comparatively lower disease burden, particularly the eastern coastal areas, which have the lowest overall disease burden. This can be attributed to both socioeconomic status [66] and dietary habits, such that diets are relatively healthful in Eastern regions such as Shanghai, Guangdong, Jiangsu, and Zhejiang, where coastal cities consume more seafood [67]. In contrast, the Northeastern region has heavier flavor profiles in its diet, which means a higher sodium intake [68]. The varying levels of economic development and dietary habits across regions can lead to an emphasis on differing dietary factors that require specific interventions. For instance, in Beijing and Shanghai, where economic levels are high, the disease burden caused by high red meat consumption is higher than in other areas [69]. These factors can serve as focal points for dietary interventions in different regions.

This study also has certain limitations. First, it lacks data on food intake levels across China, which hinders comprehensive analysis. In addition, although stratified analyses by urban-rural classification and socioeconomic status could provide more granular insights, such analyses were not feasible due to the lack of data in the GBD 2021 dataset. Future studies incorporating primary data collection or additional contextual datasets may help address these gaps. However, this study provides an analysis of the burden of diet-related diseases amongst Chinese adults aged 25 years and older, with a particular focus on regional variations and the burden attributed to specific dietary risk factors. This evidence can better inform the development of targeted dietary interventions.

### Conclusion

In conclusion, in China, the burden of diseases related to diet remains significant. With rapid economic growth and shifting dietary patterns, the disease burden caused by dietary risk factors should receive greater attention. In response, tailored and impactful nutrition policies and strategies that address diet-related disease burdens in China need to be developed and implemented.

## Supplementary material

10.2196/72978 Multimedia Appendix 1 Guidelines for Accurate and Transparent Health Estimates Reporting checklist, disease classifications & levels, dietary risk–disease references, and supplementary tables.

10.2196/72978 Multimedia Appendix 2 Deaths number and ASR-deaths.

## Abbreviations

ASR: age-standardized rate
DALY: disability-adjusted life year
EAPC: estimating annual percentage change
GATHER: Guidelines for Accurate and Transparent Health Estimates Reporting
GBD: The Global Burden of Disease
NCD: noncommunicable diseases
PAF: population attributable fraction
SSB: sugar-sweetened beverage
STROBE: STrengthening the Reporting of OBservational studies in Epidemiology
TMREL: theoretical minimum risk exposure level
UI: uncertainty interval
WHO: World Health Organization
YLD: year lived with disability
YLL: year of life lost
