# Supplementary material for: Dietary Risk Factors and Associated Disease Burden Among Chinese Adults Aged 25 Years and Older: Systematic Analysis of the Global Burden of Disease Study 2021
Source: JMIR Public Health Surveill. 2025 Aug 25;11:e72978. doi: 10.2196/72978 (PMC12441878; doi:10.2196/72978)
Supplement: Multimedia Appendix 1 [file publichealth-v11-e72978-s001.docx]

**Table S1: GATHER-Checklist of item that should be included [1].**

| **#** | **Checklist item** | **Reference** |
| --- | --- | --- |
| **Objectives and funding** | | |
| 1 | Define the indicators, populations, and time periods for which estimates were made. | Main text (Methods), the details have been published previously. |
| 2 | List the funding sources for the work. | No funding. |
| **Data Inputs** | | |
| *For all data inputs from multiple sources that are synthesized as part of the study:* | | |
| 3 | Describe how the data were identified and how the data were accessed. | Main text (Methods), the details have been published previously. |
| 4 | Specify the inclusion and exclusion criteria. Identify all ad-hoc exclusions. | Main text (Methods), the details have been published previously. |
| 5 | Provide information on all included data sources and their main characteristics. For each data source used, report reference information or contact name/institution, population represented, data collection method, year(s) of data collection, sex and age range, diagnostic criteria or measurement method, and sample size, as relevant. | Main text (Methods)  Available via online data source tools: (http://ghdx.healthdata.org/gbd-2021/data-input-sources). |
| 6 | Identify and describe any categories of input data that have potentially important biases (e.g., based on characteristics listed in item 5). | Main text (Methods and limitations), the details have been published previously. |
| *For data inputs that contribute to the analysis but were not synthesized as part of the study:* | | |
| 7 | Describe and give sources for any other data inputs. | Available via online data source tools: (http://ghdx.healthdata.org/gbd-2021/data-input-sources). |
| *For all data inputs:* | | |
| 8 | Provide all data inputs in a file format from which data can be efficiently extracted (e.g., a spreadsheet as opposed to a PDF), including all relevant meta-data listed in item 5. For any data inputs that cannot be shared due to ethical or legal reasons, such as third-party ownership, provide a contact name or the name of the institution that retains the right to the data. | Available via online data source tools: (http://ghdx.healthdata.org/gbd-2021/data-input-sources) |
| **Data analysis** | | |
| 9 | Provide a conceptual overview of the data analysis method. A diagram may be helpful. | Main text (Methods)  Flow diagrams were available online:  (http://ghdx.healthdata.org/gbd-2021/code) |
| 10 | Provide a detailed description of all steps of the analysis, including mathematical formulae. This description should cover, as relevant, data cleaning, data pre-processing, data adjustments and weighting of data sources, and mathematical or statistical model(s). | Main text (Methods), the details have been published previously. |
| 11 | Describe how candidate models were evaluated and how the final model(s) were selected. | The details have been published previously. |
| 12 | Provide the results of an evaluation of model performance, if done, as well as the results of any relevant sensitivity analysis. | The details have been published previously. |
| 13 | Describe methods for calculating uncertainty of the estimates. State which sources of uncertainty were, and were not, accounted for in the uncertainty analysis. | Main text (Methods) |
| 14 | State how analytic or statistical source code used to generate estimates can be accessed. | Statistical analysis code was available online:  (http://ghdx.healthdata.org/gbd-2021/code) |
| **Results and Discussion** | | |
| 15 | Provide published estimates in a file format from which data can be efficiently extracted. | Results, and online data tools: (http://ghdx.healthdata.org/gbd-2021) |
| 16 | Report a quantitative measure of the uncertainty of the estimates (e.g. uncertainty intervals). | Results, and online data tools: (http://ghdx.healthdata.org/gbd-2021) |
| 17 | Interpret results in light of existing evidence. If updating a previous set of estimates, describe the reasons for changes in estimates. | Main text (Discussion) |
| 18 | Discuss limitations of the estimates. Include a discussion of any modelling assumptions or data limitations that affect interpretation of the estimates. | Main text (Discussion) |

**Table S2: Cause hierarchy for all causes, cardiovascular disease, diabetes and kidney diseases, and neoplasms.**

| Cause | level |
| --- | --- |
| All causes | 0 |
| Cardiovascular diseases | 2 |
| Diabetes and kidney diseases | 2 |
| Neoplasms | 2 |

###### Table S3: References to demonstrate the relationship between dietary risks factors and cardiovascular diseases, diabetes and kidney diseases, and neoplasms.

| Risk | Cause | References |
| --- | --- | --- |
| Diet high in red meat | Cardiovascular diseases | Shi W, Huang X, Schooling CM, Zhao JV. Red meat consumption, cardiovascular diseases, and diabetes: a systematic review and meta-analysis. Eur Heart J. 2023 Jul; PMID: 37264855 [2]. |
| Diet high in red meat | Diabetes mellitus | Shi W, Huang X, Schooling CM, Zhao JV. Red meat consumption, cardiovascular diseases, and diabetes: a systematic review and meta-analysis. Eur Heart J. 2023 Jul; PMID: 37264855 [2]. |
| Diet high in red meat | Colon and rectum cancer | Veettil SK, Wong TY, Loo YS, Playdon MC, Lai NM, Giovannucci EL, Chaiyakunapruk N. Role of Diet in Colorectal Cancer Incidence: Umbrella Review of Meta-analyses of Prospective Observational Studies. JAMA Netw Open. 2021 Feb; PMID: 33591366 [3]. |
| Diet low in seafood omega - 3 fats | Cardiovascular diseases | Abdelhamid AS, Brown TJ, Brainard JS, Biswas P, Thorpe GC, Moore HJ, Deane KH, AlAbdulghafoor FK, Summerbell CD, Worthington HV, Song F, Hooper L. Omega-3 fatty acids for the primary and secondary prevention of cardiovascular disease. Cochrane Database Syst Rev. 2018 Jul 18;7(7):CD003177. doi: 10.1002/14651858.CD003177.pub3. Update in: Cochrane Database Syst Rev. 2018 Nov; PMID: 30019766 [4]. |
| Diet low in nuts and seeds | Cardiovascular diseases | Houston L, Probst YC, Chandra Singh M, Neale EP. Tree Nut and Peanut Consumption and Risk of Cardiovascular Disease: A Systematic Review and Meta-Analysis of Randomized Controlled Trials. Adv Nutr. 2023 Sep; PMID: 37149262 [5]. |
| Diet high in trans fats | Cardiovascular diseases | Hooper L, Martin N, Jimoh OF, Kirk C, Foster E, Abdelhamid AS. Reduction in saturated fat intake for cardiovascular disease. Cochrane Database Syst Rev. 2020 Aug; PMID: 32827219 [6]. |
| Dietary risks | Cardiovascular diseases | Chareonrungrueangchai K, Wongkawinwoot K, Anothaisintawee T, Reutrakul S. Dietary Factors and Risks of Cardiovascular Diseases: An Umbrella Review. Nutrients. 2020 Apr; PMID: 32326404 [7]. |
| Diet low in nuts and seeds | Diabetes melliyus | Luo C, Zhang Y, Ding Y, Shan Z, Chen S, Yu M, Hu FB, Liu L. Nut consumption and risk of type 2 diabetes, cardiovascular disease, and all-cause mortality: a systematic review and meta-analysis. Am J Clin Nutr. 2014 Jul; PMID: 24847854 [8]. |
| Diet high in fiber | Colon and rectum cancer | Veettil SK, Wong TY, Loo YS, Playdon MC, Lai NM, Giovannucci EL, Chaiyakunapruk N. Role of Diet in Colorectal Cancer Incidence: Umbrella Review of Meta-analyses of Prospective Observational Studies. JAMA Netw Open. 2021 Feb; PMID: 33591366 [3]. |
| Diet low in calcium | Colon and rectum cancer | Veettil SK, Wong TY, Loo YS, Playdon MC, Lai NM, Giovannucci EL, Chaiyakunapruk N. Role of Diet in Colorectal Cancer Incidence: Umbrella Review of Meta-analyses of Prospective Observational Studies. JAMA Netw Open. 2021 Feb; PMID: 33591366 [3]. |
| Diet high in vegetables | Breast cancer | Boushey C, Ard J, Bazzano L, Heymsfield S, Mayer-Davis E, Sabaté J, Snetselaar L, Van Horn L, Schneeman B, English LK, Bates M, Callahan E, Butera G, Terry N, Obbagy J. Dietary Patterns and Breast, Colorectal, Lung, and Prostate Cancer: A Systematic Review [Internet]. Alexandria (VA): USDA Nutrition Evidence Systematic Review; 2020 Jul. PMID: 35129907 [9]. |
| Diet high in fruits | Breast cancer | Boushey C, Ard J, Bazzano L, Heymsfield S, Mayer-Davis E, Sabaté J, Snetselaar L, Van Horn L, Schneeman B, English LK, Bates M, Callahan E, Butera G, Terry N, Obbagy J. Dietary Patterns and Breast, Colorectal, Lung, and Prostate Cancer: A Systematic Review [Internet]. Alexandria (VA): USDA Nutrition Evidence Systematic Review; 2020 Jul. PMID: 35129907 [9]. |
| Diet low in whole grains | Breast cancer | Boushey C, Ard J, Bazzano L, Heymsfield S, Mayer-Davis E, Sabaté J, Snetselaar L, Van Horn L, Schneeman B, English LK, Bates M, Callahan E, Butera G, Terry N, Obbagy J. Dietary Patterns and Breast, Colorectal, Lung, and Prostate Cancer: A Systematic Review [Internet]. Alexandria (VA): USDA Nutrition Evidence Systematic Review; 2020 Jul. PMID: 35129907 [9]. |
| Diet high sugar-sweetened beverages | Pancreatic cancer | Qin X, Chen J, Jia G, Yang Z. Dietary Factors and Pancreatic Cancer Risk: An Umbrella Review of Meta-Analyses of Prospective Observational Studies. Adv Nutr. 2023 May; PMID: 36849084 [10]. |
| Diet high sugar-sweetened beverages | Diabetes mellitus | Imamura F, O'Connor L, Ye Z, Mursu J, Hayashino Y, Bhupathiraju SN, Forouhi NG. Consumption of sugar sweetened beverages, artificially sweetened beverages, and fruit juice and incidence of type 2 diabetes: systematic review, meta-analysis, and estimation of population attributable fraction. Br J Sports Med. 2016 Apr; PMID: 27044603 [11]. |
| Diet high sugar-sweetened beverages | Cardiovascular disease | Yin J, Zhu Y, Malik V, Li X, Peng X, Zhang FF, Shan Z, Liu L. Intake of Sugar-Sweetened and Low-Calorie Sweetened Beverages and Risk of Cardiovascular Disease: A Meta-Analysis and Systematic Review. Adv Nutr. 2021 Feb; PMID: 32696948 [12]. |
| Diet high in sodium | Cardiovascular diseases | Aburto NJ, Hanson S, Gutierrez H, Hooper L, Elliott P, Cappuccio FP. Effect of increased potassium intake on cardiovascular risk factors and disease: systematic review and meta-analyses. BMJ. 2013 Apr; PMID: 23558164 [13]. |
| Diet high in sodium | Stomach cancer | D'Elia L, Rossi G, Ippolito R, Cappuccio FP, Strazzullo P. Habitual salt intake and risk of gastric cancer: a meta-analysis of prospective studies. Clin Nutr. 2012 Aug; PMID: 22296873 [14]. |
| Diet high in processed meat | Neoplasms | Kliemann N, Rauber F, Bertazzi Levy R, Viallon V, Vamos EP, Cordova R, Freisling H, Casagrande C, Nicolas G, Aune D, Tsilidis KK, Heath A, Schulze MB, Jannasch F, Srour B, Kaaks R, Rodriguez-Barranco M, Tagliabue G, Agudo A, Panico S, Ardanaz E, Chirlaque MD, Vineis P, Tumino R, Perez-Cornago A, Andersen JLM, Tjønneland A, Skeie G, Weiderpass E, Monteiro CA, Gunter MJ, Millett C, Huybrechts I. Food processing and cancer risk in Europe: results from the prospective EPIC cohort study. Lancet Planet Health. 2023 Mar; PMID: 36889863 [15]. |
| Diet high in processed meat | Diabetes mellitus | Micha R, Wallace SK, Mozaffarian D. Red and processed meat consumption and risk of incident coronary heart disease, stroke, and diabetes mellitus: a systematic review and meta-analysis. Circulation. 2010 Jun; PMID: 20479151 [16]. |
| Diet low in polyunsaturated fats | Cardiovascular disease | Djuricic I, Calder PC. Beneficial Outcomes of Omega-6 and Omega-3 Polyunsaturated Fatty Acids on Human Health: An Update for 2021. Nutrients. 2021 Jul; PMID: 34371930 [17]. |

**Table S4: Number and age-standardized proportion and rate of deaths, YLLs, YLDs, and DALYs attributable to dietary risk factors among adults in 2021, in China.**

|  | **Deaths** | | **YLLs** | | **YLDs** | | **DALYs** | |
| --- | --- | --- | --- | --- | --- | --- | --- | --- |
|  | **2021** | **Percentage change, 1990-2021** | **2021** | **Percentage change, 1990-2021** | **2021** | **Percentage change, 1990-2021** | **2021** | **Percentage change, 1990-2021** |
| **All causes** | | | | | | | | |
| Absolute number,  thousands | 1704.02 (687.92, 2684.78) | 68.53% (19.31%, 112.32%) | 34186.08 (14575.66, 52839.91) | 35.33% (0.44%, 69.77%) | 4207.73 (1567.38, 6728.75) | 192.17% (132.9%, 220.34%) | 38393.81 (16213.07, 58607.05) | 43.79% (7.97%, 78.13%) |
| Age-standardized  percent | 14.01% (5.68%, 21.43%) | 10.44% (-15.95%, 23.9%) | 12.15% (5.17%, 18.06%) | 32.27% (3.39%, 48.18%) | 2.31% (0.92%, 3.54%) | 33.18% (1.79%, 47.19%) | 8.34% (3.61%, 12.42%) | 10.06% (-15.22%, 25.14%) |
| Age-standardized rate,  per 100,000 people | 90.37 (35.35, 143.15) | -40.59% (-58.29%, -26.53%) | 1688.52 (689.96, 2601.5) | -45.59% (-60.08%, -32.27%) | 204.17 (75.67, 326.07) | 26.21% (-2.64%, 39.45%) | 1892.69 (775.77, 2904.23) | -42.04% (-57.32%, -28.45%) |
| **Cardiovascular diseases** | | | | | | | | |
| Absolute number,  thousands | 1449.88 (594.79, 2244.04) | 74.71% (17.31%, 123.45%) | 28353.16 (12271.3, 42425.37) | 40.27% (-0.62%, 79.61%) | 1913 (786.69, 3080.93) | 132.03% (86.17%, 158.28%) | 30266.17 (13228.42, 45225.09) | 43.86% (3.2%, 81.7%) |
| Age-standardized  percent | 27.72% (11.18%, 41.4%) | -12.04% (-37.03%, -1.18%) | 29.9% (12.9%, 42.73%) | -9.15% (-29.74%, -0.2%) | 21.94% (9.96%, 32.54%) | -12.92% (-28.92%, -5.24%) | 29.26% (12.65%, 41.87%) | -10.12% (-30.62%, -1.25%) |
| Age-standardized rate,  per 100,000 people | 77.76 (30.45, 121.22) | -39.54% (-59.31%, -24.82%) | 1409.55 (584.61, 2114) | -44.42% (-61.27%, -29.33%) | 90.03 (36.97, 145.22) | -4.91% (-22.85%, 3.94%) | 1499.58 (632.89, 2247.22) | -43% (-59.68%, -28.42%) |
| **Diabetes and kidney diseases** | | | | | | | | |
| Absolute number,  thousands | 76.77 (32.01, 121.77) | 140.33% (73.68%, 206.38%) | 1581.38 (654.4, 2528.97) | 99.25% (42.61%, 156.94%) | 2109.35 (403.13, 3857.85) | 282.99% (195.41%, 315.55%) | 3690.73 (1021.61, 6162.48) | 174.53% (88.06%, 219.76%) |
| Age-standardized  percent | 19.43% (8.3%, 29.38%) | 1.98% (-17.23%, 13.9%) | 18.11% (7.68%, 27.67%) | 13.73% (-9.82%, 27.41%) | 21.42% (4.36%, 35.64%) | 25.19% (-8.7%, 35.89%) | 19.89% (6.27%, 32.18%) | 21.62% (-17.61%, 36.95%) |
| Age-standardized rate,  per 100,000 people | 3.91 (1.65, 6.18) | -18.03% (-39.49%, 2.33%) | 75.81 (31.53, 120.59) | -22.17% (-43.05%, -1.19%) | 105.35 (20.53, 193.47) | 76.42% (25.23%, 94.18%) | 181.17 (50.05, 301.66) | 15.3% (-24.1%, 36.8%) |
| **Neoplasms** |  |  |  |  |  |  |  |  |
| Absolute number,  thousands | 176.53 (60.8, 367.46) | 20.07% (-14.96%, 77.1%) | 4229.48 (1429.36, 8797.48) | 0.95% (-29.13%, 46%) | 177.77 (47.72, 340.07) | 201.62% (108.89%, 317.44%) | 4407.25 (1479.11, 9106.09) | 3.73% (-27.44%, 49.54%) |
| Age-standardized  percent | 6.3% (2.13%, 12.76%) | -36.51% (-52.18%, -14.26%) | 5.98% (1.96%, 12.19%) | -34.18% (-51.1%, -11.77%) | 9.18% (3.02%, 15.79%) | -9.31% (-35.25%, 10%) | 6.06% (1.98%, 12.28%) | -33.34% (-50.67%, -10.54%) |
| Age-standardized rate,  per 100,000 people | 8.66 (2.98, 17.89) | -53.03% (-66.42%, -32.9%) | 202.08 (68.33, 417.31) | -56.33% (-69.35%, -37.31%) | 8.41 (2.25, 16.11) | 25.55% (-13.97%, 70.26%) | 210.49 (70.5, 431.87) | -55.17% (-68.61%, -35.86%) |

**Table S5: Number and age-standardized proportion and rate of deaths, YLLs, YLDs, and DALYs attributable to dietary risk factors by age in 2021, in China.**

|  | **metric** | **Deaths** | | **YLLs** | | **YLDs** | | **DALYs** | |
| --- | --- | --- | --- | --- | --- | --- | --- | --- | --- |
|  |  | **2021** | **Percentage change, 1990-2021** | **2021** | **Percentage change, 1990-2021** | **2021** | **Percentage change, 1990-2021** | **2021** | **Percentage change, 1990-2021** |
| **All causes** | | | | | | | | | |
| 25-29 years | Absolute number,  thousands | 2.83 (0.55, 4.12) | -49.48% (-67.71%, -33.03%) | 177.01 (34.57, 257.49) | -49.7% (-67.86%, -33.33%) | 95.52 (19.97, 167.02) | 58.34% (-5.15%, 100.84%) | 272.52 (54.88, 404.72) | -33.89% (-55.6%, -16.68%) |
| 30-34 years | Absolute number,  thousands | 8.31 (2.09, 12.09) | -10.75% (-34.1%, 17.33%) | 478.63 (120.29, 696.02) | -10.67% (-34.04%, 17.44%) | 166.7 (38.52, 293.1) | 156.97% (53.77%, 218.33%) | 645.33 (165.61, 915.26) | 7.44% (-20.32%, 36.06%) |
| 35-39 years | Absolute number,  thousands | 13.44 (4.34, 19.51) | -33.02% (-51.33%, -11.92%) | 709.97 (229.34, 1030.49) | -32.94% (-51.27%, -11.82%) | 178.72 (46.19, 294.07) | 89.68% (13.58%, 130.26%) | 888.69 (282.57, 1235.49) | -22.92% (-41.85%, -3.36%) |
| 40-44 years | Absolute number,  thousands | 21.6 (7.83, 31.66) | -26.23% (-44.89%, -2.5%) | 1032.54 (374.13, 1513.16) | -26.41% (-45.03%, -2.75%) | 195.06 (54.5, 326.68) | 114.96% (37.34%, 159.65%) | 1227.59 (433.45, 1751.74) | -17.82% (-37.77%, 5.55%) |
| 45-49 years | Absolute number,  thousands | 38.06 (16.13, 55.7) | 4.2% (-24.3%, 38.58%) | 1629.68 (690.76, 2384.82) | 3.92% (-24.51%, 38.2%) | 309.35 (91.75, 504.17) | 198.49% (105.38%, 247.69%) | 1939.03 (791.64, 2799.42) | 15.98% (-11.67%, 49.05%) |
| 50-54 years | Absolute number,  thousands | 71.85 (31.17, 108.18) | 10.49% (-19.94%, 45.26%) | 2737.79 (1187.62, 4122.67) | 10.49% (-19.94%, 45.27%) | 446.14 (142.18, 727.66) | 225.87% (134.53%, 282.84%) | 3183.93 (1349.13, 4715.12) | 21.77% (-8.1%, 55.81%) |
| 55-59 years | Absolute number,  thousands | 105.62 (51.56, 162.43) | 8.54% (-20.78%, 42.29%) | 3543 (1729.98, 5448.89) | 8.63% (-20.72%, 42.4%) | 538.22 (200.04, 884.66) | 218.39% (146.03%, 267.76%) | 4081.22 (1946.93, 6175.99) | 18.96% (-11.67%, 51.49%) |
| 60-64 years | Absolute number,  thousands | 117.94 (54.75, 180.17) | -1.58% (-27.22%, 25.99%) | 3393.96 (1575.62, 5184.81) | -1.9% (-27.46%, 25.57%) | 458.09 (165.23, 753.82) | 151.43% (94.96%, 182.95%) | 3852.06 (1740.33, 5769.49) | 5.77% (-20.5%, 31.89%) |
| 65-69 years | Absolute number,  thousands | 207.34 (94.7, 317.34) | 38.45% (4.93%, 78.88%) | 5026.83 (2295.93, 7693.89) | 38.26% (4.8%, 78.65%) | 642.32 (242.23, 1045.14) | 223.78% (153.04%, 266.55%) | 5669.15 (2554.15, 8651.49) | 47.86% (11.7%, 86.08%) |
| 70-74 years | Absolute number,  thousands | 248.49 (103.86, 386.39) | 51.49% (9.26%, 95.02%) | 4964.52 (2074.82, 7719.67) | 51.51% (9.26%, 95.04%) | 516.48 (189.12, 868.89) | 214.86% (140.89%, 257.48%) | 5481 (2291.16, 8484.32) | 59.3% (16.81%, 101.26%) |
| 75-79 years | Absolute number,  thousands | 243.02 (100.51, 392.09) | 69% (22.26%, 115.65%) | 3875.58 (1602.59, 6252.07) | 68.17% (21.63%, 114.66%) | 344.07 (124.3, 573.03) | 219.51% (139.52%, 265.73%) | 4219.64 (1732.92, 6812.11) | 74.93% (26.75%, 121.17%) |
| 80+ years | Absolute number,  thousands | 625.52 (194.6, 1046.33) | 266.68% (115.36%, 357.17%) | 6616.59 (2046.43, 11036.47) | 243.3% (103.83%, 327.11%) | 317.05 (115.98, 555.26) | 365.66% (242.16%, 438.5%) | 6933.65 (2172.57, 11528.65) | 247.48% (108.23%, 329.96%) |
| **Cardiovascular diseases** | | | | | | | | | |
| 25-29 years | Absolute number,  thousands | 2.2 (0.33, 3.25) | -48.52% (-68.74%, -29.71%) | 137.75 (20.59, 203.39) | -48.74% (-68.87%, -30.02%) | 14.67 (1.33, 27.81) | -49.27% (-88.54%, -30.33%) | 152.42 (23.55, 230.22) | -48.8% (-70.29%, -30.88%) |
| 30-34 years | Absolute number,  thousands | 6.31 (1.36, 9.21) | -8.37% (-36.14%, 22.63%) | 363.4 (78.17, 529.96) | -8.29% (-36.09%, 22.74%) | 31.68 (8.16, 54.79) | -1.02% (-58.43%, 27.88%) | 395.08 (83.91, 581.12) | -7.75% (-35.37%, 22.9%) |
| 35-39 years | Absolute number,  thousands | 10.36 (3.28, 14.96) | -30.42% (-52.43%, -6.82%) | 547.19 (173.29, 790.19) | -30.33% (-52.36%, -6.7%) | 41.95 (13.86, 68.81) | -10.03% (-45.6%, 12.35%) | 589.14 (198.38, 850.2) | -29.19% (-51.75%, -5.79%) |
| 40-44 years | Absolute number,  thousands | 17 (6.33, 24.31) | -22.11% (-44.66%, 4.9%) | 812.75 (302.38, 1161.74) | -22.32% (-44.81%, 4.63%) | 54.57 (22.64, 86.84) | 21.03% (-9.62%, 46.96%) | 867.31 (333.89, 1240.37) | -20.53% (-42.57%, 5.71%) |
| 45-49 years | Absolute number,  thousands | 29.69 (13.64, 43.39) | 8.9% (-22.83%, 42.96%) | 1271.12 (584.04, 1857.9) | 8.61% (-23.03%, 42.58%) | 104 (49.03, 160.03) | 92.64% (54.38%, 127.6%) | 1375.11 (645.48, 1991.2) | 12.32% (-19.09%, 45.82%) |
| 50-54 years | Absolute number,  thousands | 56.26 (25.37, 82.4) | 13.85% (-19.33%, 48.5%) | 2143.87 (966.59, 3140.15) | 13.86% (-19.33%, 48.51%) | 166.72 (70.8, 262.93) | 130.55% (89.7%, 163.51%) | 2310.59 (1056.81, 3362.32) | 18.17% (-14.51%, 51.82%) |
| 55-59 years | Absolute number,  thousands | 83.33 (42.74, 121.27) | 10.97% (-21.74%, 46.66%) | 2795.12 (1434.08, 4067.69) | 11.05% (-21.67%, 46.76%) | 226.41 (99.04, 361.85) | 147.08% (104.3%, 184.61%) | 3021.53 (1545.66, 4387.78) | 15.83% (-17.23%, 50.71%) |
| 60-64 years | Absolute number,  thousands | 95.22 (45.54, 139.08) | 0.39% (-29.33%, 29.21%) | 2740.11 (1310.38, 4002.24) | 0.05% (-29.57%, 28.78%) | 216.79 (90.47, 345.16) | 108.09% (66.76%, 138.57%) | 2956.9 (1398.32, 4278.99) | 4.01% (-25.74%, 32.9%) |
| 65-69 years | Absolute number,  thousands | 170.94 (80.61, 252.71) | 39.51% (1.83%, 81.31%) | 4144.67 (1954.25, 6127.34) | 39.33% (1.7%, 81.07%) | 350.68 (156.04, 562.12) | 178.99% (117.05%, 219.12%) | 4495.35 (2121.85, 6608.41) | 45% (5.08%, 86.35%) |
| 70-74 years | Absolute number,  thousands | 207.94 (86.84, 313.74) | 51.53% (4.43%, 98.29%) | 4154.71 (1734.47, 6269.08) | 51.57% (4.42%, 98.34%) | 300.5 (106.9, 496.2) | 178.44% (102.33%, 222.72%) | 4455.21 (1851.84, 6715.86) | 56.37% (8.16%, 102.18%) |
| 75-79 years | Absolute number,  thousands | 206.74 (84.32, 327.44) | 68.01% (15.02%, 119.18%) | 3297.13 (1344.82, 5221.95) | 67.19% (14.4%, 118.1%) | 209.92 (74.41, 360.29) | 192% (94.09%, 254.59%) | 3507.05 (1414.8, 5538.58) | 71.58% (16.73%, 121.63%) |
| 80+ years | Absolute number,  thousands | 563.88 (168.89, 940.1) | 269.61% (103.32%, 369.51%) | 5945.34 (1767.06, 9879.31) | 245.59% (92.4%, 337.29%) | 195.14 (56.94, 353.49) | 341.14% (181.13%, 438.06%) | 6140.48 (1855.31, 10206.75) | 247.99% (96.28%, 337.38%) |
| **Diabetes and kidney diseases** | | | | | | | | | |
| 25-29 years | Absolute number,  thousands | 0.09 (0.04, 0.16) | -48.89% (-70.98%, -18.31%) | 5.93 (2.76, 10.01) | -49.12% (-71.11%, -18.67%) | 79.39 (14.62, 149.27) | 162.59% (109.17%, 219.4%) | 85.32 (16.83, 157.65) | 103.71% (2.63%, 158.09%) |
| 30-34 years | Absolute number,  thousands | 0.29 (0.13, 0.48) | 5.76% (-33.6%, 56.13%) | 16.44 (7.55, 27.36) | 5.87% (-33.53%, 56.27%) | 131.04 (25.67, 247.05) | 323.01% (246.02%, 408.84%) | 147.47 (32.76, 273.01) | 217.13% (104.22%, 291.75%) |
| 35-39 years | Absolute number,  thousands | 0.45 (0.2, 0.74) | -19.51% (-48.41%, 15.43%) | 23.87 (10.63, 38.93) | -19.42% (-48.35%, 15.56%) | 131.08 (23.26, 247.37) | 198.29% (145.04%, 253.49%) | 154.95 (34.06, 281.21) | 110.62% (23.93%, 161.09%) |
| 40-44 years | Absolute number,  thousands | 0.73 (0.32, 1.18) | -9.11% (-40.91%, 31.23%) | 34.99 (15.38, 56.59) | -9.33% (-41.05%, 30.94%) | 132.44 (19.96, 246.96) | 220.88% (158.62%, 286.5%) | 167.43 (37.79, 296.35) | 109.64% (23.52%, 166.13%) |
| 45-49 years | Absolute number,  thousands | 1.58 (0.61, 2.6) | 39.95% (-6.7%, 90.69%) | 67.66 (26.06, 111.32) | 39.57% (-6.94%, 90.22%) | 192.6 (33.12, 362.6) | 329.37% (244.82%, 411.61%) | 260.26 (58.12, 470.96) | 178.85% (79.35%, 246.38%) |
| 50-54 years | Absolute number,  thousands | 3.2 (1.23, 5.29) | 59.99% (7.02%, 122.37%) | 122.06 (46.77, 201.73) | 59.98% (7.01%, 122.36%) | 259.27 (39.55, 487.04) | 349.77% (250.25%, 433.25%) | 381.33 (87.27, 668.55) | 184.7% (78.15%, 248.95%) |
| 55-59 years | Absolute number,  thousands | 5.19 (2.16, 8.51) | 65.33% (17.33%, 129.86%) | 174.23 (72.41, 285.47) | 65.49% (17.45%, 130.09%) | 287.39 (49.78, 538.84) | 319.82% (226.51%, 404.81%) | 461.61 (129.45, 796.29) | 165.7% (79.99%, 229.11%) |
| 60-64 years | Absolute number,  thousands | 5.93 (2.22, 9.91) | 51.95% (4.11%, 107.22%) | 170.72 (63.74, 285.05) | 51.45% (3.77%, 106.55%) | 219.79 (39.94, 414.66) | 218.23% (127.62%, 272.83%) | 390.51 (109.69, 680.87) | 114.82% (45.55%, 170.37%) |
| 65-69 years | Absolute number,  thousands | 10.83 (4.47, 17.59) | 125.5% (58.51%, 195.66%) | 262.53 (108.41, 426.33) | 125.14% (58.26%, 195.15%) | 262.92 (53.97, 473.67) | 311.16% (187.34%, 403.37%) | 525.45 (161.15, 881.5) | 191.02% (95.82%, 261.57%) |
| 70-74 years | Absolute number,  thousands | 12.89 (4.72, 21.26) | 146.39% (71.66%, 233.08%) | 257.49 (94.22, 424.57) | 146.42% (71.68%, 233.18%) | 190.36 (36.74, 352.76) | 291.98% (155.69%, 370.84%) | 447.86 (133.83, 745.17) | 192.61% (99.44%, 274.55%) |
| 75-79 years | Absolute number,  thousands | 12.27 (4.99, 19.71) | 156.01% (75.29%, 245.23%) | 195.6 (79.49, 314.17) | 154.74% (74.48%, 243.55%) | 117.04 (26.9, 209.66) | 277.58% (143.78%, 354.63%) | 312.64 (104.65, 509.83) | 190.07% (95.48%, 267.28%) |
| 80+ years | Absolute number,  thousands | 23.3 (10.24, 36.75) | 355.55% (232.57%, 467.91%) | 249.86 (109.37, 393.18) | 333.77% (213.66%, 442.12%) | 106.03 (32.59, 186.28) | 409.87% (244.93%, 512.41%) | 355.9 (141.17, 569.92) | 353.96% (232.14%, 448.71%) |
| **Neoplasms** | | | | | | | | | |
| 25-29 years | Absolute number,  thousands | 0.53 (0.18, 0.96) | -52.55% (-64.82%, -37.49%) | 32.86 (11.51, 60.15) | -52.77% (-64.98%, -37.77%) | 1.18 (0.27, 2.29) | 32.59% (-9.32%, 76.68%) | 34.03 (11.85, 62.13) | -51.69% (-64.47%, -35.91%) |
| 30-34 years | Absolute number,  thousands | 1.7 (0.55, 3.17) | -19.7% (-41.21%, 7.61%) | 97.9 (31.49, 182.8) | -19.61% (-41.13%, 7.74%) | 3.65 (0.82, 7.14) | 121.55% (50.66%, 201.88%) | 101.55 (32.35, 188.6) | -17.72% (-40%, 10.43%) |
| 35-39 years | Absolute number,  thousands | 2.61 (0.79, 5.02) | -42.53% (-57.54%, -23.57%) | 137.94 (41.78, 265.36) | -42.48% (-57.5%, -23.5%) | 5.33 (1.12, 10.73) | 63.12% (9.47%, 122.96%) | 143.27 (42.81, 274.29) | -41.06% (-56.58%, -21.67%) |
| 40-44 years | Absolute number,  thousands | 3.84 (1.14, 7.64) | -41.41% (-58.38%, -16.35%) | 183.58 (54.28, 364.91) | -41.55% (-58.46%, -16.54%) | 7.73 (1.46, 15.79) | 89.77% (12.37%, 164.52%) | 191.31 (55.86, 381.59) | -39.86% (-57.51%, -14.21%) |
| 45-49 years | Absolute number,  thousands | 6.75 (1.93, 13.85) | -15.79% (-42.14%, 25.88%) | 288.94 (82.54, 592.91) | -16.04% (-42.3%, 25.55%) | 12.3 (2.27, 24.91) | 180.32% (62.75%, 298.53%) | 301.24 (84.7, 616.21) | -13.56% (-41.17%, 29.32%) |
| 50-54 years | Absolute number,  thousands | 12.31 (3.52, 26.17) | -8.35% (-41.86%, 39.86%) | 469.09 (134.3, 997.16) | -8.37% (-41.87%, 39.83%) | 19.62 (4.2, 38.69) | 202.3% (83.45%, 332.03%) | 488.71 (138.26, 1031.79) | -5.73% (-40.02%, 43.71%) |
| 55-59 years | Absolute number,  thousands | 17.03 (5.38, 36.87) | -9.61% (-42.66%, 40%) | 571.2 (180.48, 1236.88) | -9.53% (-42.6%, 40.13%) | 23.84 (5.9, 46.64) | 186.45% (79.42%, 309.21%) | 595.04 (188.45, 1287.34) | -6.98% (-41.17%, 42.73%) |
| 60-64 years | Absolute number,  thousands | 16.71 (5.28, 35.97) | -19.58% (-47.73%, 21%) | 480.79 (152.01, 1035.23) | -19.85% (-47.91%, 20.61%) | 20.97 (5.53, 40.11) | 153.65% (68.01%, 273.6%) | 501.76 (158.49, 1071.65) | -17.49% (-46.23%, 23.77%) |
| 65-69 years | Absolute number,  thousands | 25.44 (8.2, 54.54) | 15.27% (-21.63%, 81.59%) | 616.62 (198.87, 1322.01) | 15.08% (-21.77%, 81.28%) | 27.29 (7.65, 50.43) | 246.59% (131.74%, 407.63%) | 643.91 (208.82, 1365.48) | 18.43% (-18.72%, 85.46%) |
| 70-74 years | Absolute number,  thousands | 27.53 (9.59, 58.84) | 29.72% (-13.7%, 97.91%) | 549.77 (191.6, 1174.82) | 29.68% (-13.74%, 97.79%) | 24.08 (7.74, 43.83) | 263.02% (150.97%, 428.58%) | 573.85 (200.5, 1217.67) | 33.28% (-11.12%, 102.36%) |
| 75-79 years | Absolute number,  thousands | 23.89 (8.34, 50.44) | 52.56% (7.3%, 135.06%) | 381.07 (133.03, 804.64) | 51.81% (6.75%, 133.96%) | 16.37 (5.67, 29.71) | 281.61% (173.65%, 413.17%) | 397.43 (139.13, 834.33) | 55.67% (9.37%, 139.42%) |
| 80+ years | Absolute number,  thousands | 38.2 (13.66, 75.07) | 201.26% (128.11%, 303.14%) | 419.74 (149.91, 826.97) | 186.24% (117.59%, 284.42%) | 15.41 (5.43, 27.94) | 451.15% (323.26%, 606.68%) | 435.15 (156.36, 850.62) | 191.2% (121.46%, 289.95%) |

**Table S6: Number and age-standardized proportion and rate of deaths, YLLs, YLDs, and DALYs attributable to dietary risk factors by sex in 2021, in China.**

| **Metric** | **Deaths** | | **YLLs** | | **YLDs** | | **DALYs** | |
| --- | --- | --- | --- | --- | --- | --- | --- | --- |
|  | **Male** | **Female** | **Male** | **Female** | **Male** | **Female** | **Male** | **Female** |
| **All causes** | | | | | | | | |
| Absolute number, thousands | 1018.89 (436.40, 1617.24) | 685.13 (250.51, 1145.30) | 21651.13 (9591.53, 33734.92) | 12534.95 (5079.04, 20905.77) | 2279.41 (880.52, 3606.96) | 1928.32 (680.27, 3183.07) | 23930.54 (10295.03, 36638.65) | 14463.27 (5677.07, 23362.20) |
| Age-standardized rate, per 100,000 people | 124.40 (49.56, 199.28) | 65.73 (23.36, 110.28) | 2298.80 (953.41, 3596.84) | 1166.23 (453.59, 1942.40) | 225.21 (86.13, 355.83) | 183.97 (64.49, 299.26) | 2524.01 (1034.57, 3878.49) | 1350.20 (509.30, 2179.77) |
| Age-standardized percent | 14.43% (6.22%, 21.85%) | 13.56% (4.89%, 21.42%) | 12.61% (5.47%, 18.47%) | 11.61% (4.60%, 17.88%) | 2.76% (1.15%, 4.18%) | 1.94% (0.74%, 3.03%) | 9.56% (4.20%, 14.29%) | 6.92% (2.82%, 10.79%) |
| **Cardiovascular diseases** | | | | | | | | |
| Absolute number, thousands | 872.56 (384.62, 1351.18) | 577.32 (212.00, 958.42) | 18219.43 (8262.76, 27594.67) | 10133.73 (4151.03, 16664.96) | 1060.20 (476.11, 1661.45) | 852.80 (317.50, 1391.83) | 19279.63 (8807.81, 28918.49) | 10986.53 (4565.51, 17883.96) |
| Age-standardized rate, per 100,000 people | 108.29 (43.13, 168.25) | 55.76 (19.88, 92.57) | 1954.44 (833.43, 2985.11) | 944.49 (378.20, 1549.29) | 102.39 (45.84, 160.75) | 78.56 (28.87, 128.16) | 2056.84 (884.56, 3112.12) | 1023.05 (414.21, 1668.74) |
| Age-standardized percent | 29.04% (12.26%, 42.99%) | 25.63% (9.13%, 39.30%) | 31.45% (13.98%, 44.50%) | 27.06% (10.92%, 39.94%) | 23.92% (11.39%, 34.84%) | 19.95% (8.11%, 30.48%) | 30.96% (13.90%, 43.82%) | 26.33% (10.64%, 38.91%) |
| **Diabetes and kidney diseases** | | | | | | | | |
| Absolute number, thousands | 40.15 (16.87, 66.43) | 36.62 (15.29, 62.17) | 850.04 (357.50, 1407.28) | 731.34 (288.36, 1250.04) | 1125.66 (213.64, 2064.66) | 983.69 (190.58, 1800.82) | 1975.70 (553.29, 3274.75) | 1715.03 (491.99, 2928.81) |
| Age-standardized rate, per 100,000 people | 4.77 (1.98, 7.73) | 3.38 (1.45, 5.70) | 88.62 (37.90, 144.98) | 65.98 (26.41, 112.53) | 113.66 (21.89, 208.30) | 96.87 (19.28, 177.88) | 202.28 (58.05, 334.29) | 162.85 (45.64, 279.95) |
| Age-standardized percent | 19.50% (8.28%, 29.67%) | 19.36% (7.95%, 30.13%) | 18.19% (7.78%, 27.43%) | 18.09% (7.21%, 28.17%) | 21.71% (4.25%, 36.33%) | 21.07% (4.48%, 35.30%) | 20.01% (6.37%, 32.02%) | 19.75% (6.08%, 31.93%) |
| **Neoplasms** | | | | | | | | |
| Absolute number, thousands | 105.57 (34.77, 237.33) | 70.96 (22.42, 140.38) | 2565.22 (827.82, 5826.05) | 1664.26 (496.97, 3309.09) | 88.10 (29.63, 161.21) | 89.67 (19.71, 184.46) | 2653.32 (865.13, 5999.66) | 1753.94 (517.03, 3504.61) |
| Age-standardized rate, per 100,000 people | 11.28 (3.86, 24.79) | 6.57 (2.08, 12.98) | 254.09 (83.50, 568.76) | 155.24 (46.07, 309.27) | 8.62 (2.94, 15.67) | 8.33 (1.79, 17.26) | 262.71 (87.10, 585.07) | 163.57 (47.80, 327.39) |
| Age-standardized percent | 5.92% (2.11%, 12.58%) | 6.99% (2.25%, 13.49%) | 5.66% (1.95%, 12.11%) | 6.57% (2.04%, 12.57%) | 8.43% (3.22%, 14.48%) | 9.98% (2.45%, 17.82%) | 5.72% (1.98%, 12.15%) | 6.69% (2.06%, 12.76%) |

**Table S7: Number and age-standardized proportion and rate of deaths, YLLs, YLDs, and DALYs attributable to dietary risk factors by region in 2021, in China.**

|  | **metric** | **Deaths** | | **YLLs** | | **YLDs** | | **DALYs** | |
| --- | --- | --- | --- | --- | --- | --- | --- | --- | --- |
|  |  | **2021** | **Percentage change, 1990-2021** | **2021** | **Percentage change, 1990-2021** | **2021** | **Percentage change, 1990-2021** | **2021** | **Percentage change, 1990-2021** |
| **All causes** | | | | | | | | | |
| Anhui | Absolute number,  thousands | 79.32 (33.15, 125.47) | 47.69% (1.39%, 92.93%) | 1471.54 (635.41, 2333.77) | 7.55% (-25.52%, 42.95%) | 197.21 (76.19, 315.34) | 166.02% (111.7%, 197.81%) | 1668.75 (712.5, 2623.6) | 15.69% (-16.59%, 50.59%) |
| Beijing | Absolute number,  thousands | 22.71 (7.29, 37.47) | 96.79% (25.22%, 170.2%) | 434.36 (145.11, 696.22) | 56.33% (2.26%, 115.9%) | 74.34 (25.18, 121.43) | 311.54% (181.9%, 383.53%) | 508.7 (170.21, 803.49) | 71.91% (18.93%, 130.82%) |
| Chongqing | Absolute number,  thousands | 41.35 (19.8, 68.26) | 185.41% (98.69%, 303.3%) | 780.38 (398.86, 1277.07) | 109.68% (46.02%, 203.54%) | 97.69 (35.93, 158.83) | 423.87% (323.32%, 482.83%) | 878.06 (439.33, 1400.12) | 124.67% (60.32%, 216.37%) |
| Fujian | Absolute number,  thousands | 28.46 (11.68, 46.72) | 52.72% (5.84%, 111.55%) | 567.64 (248.8, 913.17) | 20.66% (-15.62%, 68.32%) | 105.57 (36.23, 171.24) | 218.68% (136.86%, 264.81%) | 673.21 (292.76, 1058.34) | 33.68% (-3.04%, 80.74%) |
| Gansu | Absolute number,  thousands | 26.1 (8.91, 43.57) | 94% (33.03%, 163.56%) | 548.9 (200.63, 895.09) | 48.95% (4.83%, 102.15%) | 53.24 (17.12, 88.95) | 180.21% (129.55%, 210.66%) | 602.14 (220.07, 969.84) | 55.38% (11.3%, 106.96%) |
| Guangdong | Absolute number,  thousands | 80.8 (26.14, 135.62) | 69.62% (6.05%, 131.79%) | 1678.8 (559.18, 2724.13) | 48.95% (0.38%, 108.33%) | 284.89 (74.83, 475.13) | 298.93% (190.74%, 358.33%) | 1963.69 (643, 3169.71) | 63.85% (12.24%, 121.9%) |
| Guangxi | Absolute number,  thousands | 47.98 (16.65, 79.18) | 74.21% (9.14%, 136.68%) | 996.84 (360.56, 1620.41) | 45.21% (-6.51%, 102.04%) | 127.21 (40.01, 211.45) | 176.91% (100.5%, 212.21%) | 1124.05 (411.21, 1795.3) | 53.47% (2.81%, 106.54%) |
| Guizhou | Absolute number,  thousands | 39.58 (19.31, 63.12) | 47.89% (3.1%, 98.19%) | 843.47 (440.01, 1345.82) | 22.23% (-16.78%, 69.82%) | 95.25 (37.02, 152.75) | 169.12% (116.24%, 199.21%) | 938.72 (474.05, 1456.9) | 29.4% (-8.85%, 74.5%) |
| Hainan | Absolute number,  thousands | 9.28 (2.97, 15.92) | 94.19% (19.39%, 179.65%) | 187.45 (63.19, 310.37) | 65.75% (6.93%, 148.41%) | 27.81 (7.7, 46.64) | 269.67% (168.2%, 323.69%) | 215.26 (71.94, 348.51) | 78.47% (16.14%, 160.04%) |
| Hebei | Absolute number,  thousands | 125.18 (56.44, 200.45) | 85.17% (26.12%, 147.32%) | 2610.59 (1206.91, 4111.58) | 60.04% (12.1%, 120.45%) | 248.51 (97.35, 395.98) | 190.32% (125.8%, 224.92%) | 2859.1 (1301.84, 4446.03) | 66.54% (18.23%, 124.85%) |
| Heilongjiang | Absolute number,  thousands | 64.52 (16.51, 103.74) | 70.19% (5.66%, 124.78%) | 1407.39 (409.4, 2181.96) | 32.62% (-9.54%, 75.7%) | 122.57 (39.23, 200.77) | 168.32% (103.95%, 205.77%) | 1529.97 (456.94, 2385.1) | 38.22% (-3.78%, 79.64%) |
| Henan | Absolute number,  thousands | 146.33 (56.51, 237.73) | 73.87% (15.24%, 120.44%) | 2926.89 (1161.4, 4639.8) | 43.06% (-1.78%, 86.03%) | 294.43 (111.29, 472.15) | 171.24% (113.08%, 204.98%) | 3221.32 (1299.78, 5039.97) | 49.52% (3.46%, 89.99%) |
| Hong Kong | Absolute number,  thousands | 5.71 (2.43, 9.01) | 53.43% (13.21%, 102.86%) | 100.71 (45.91, 154.6) | 23.02% (-8.29%, 63.12%) | 25.5 (9.57, 40.05) | 223.92% (173.29%, 260.21%) | 126.21 (57.12, 192.51) | 40.64% (11.76%, 78.74%) |
| Hubei | Absolute number,  thousands | 77.51 (34.81, 124.69) | 30.96% (-12.36%, 71.57%) | 1512.5 (686.12, 2422.82) | 4.79% (-27.83%, 38.62%) | 169.84 (66.31, 273.31) | 158.72% (100.06%, 189.33%) | 1682.34 (753.01, 2656.97) | 11.49% (-22.58%, 44.27%) |
| Hunan | Absolute number,  thousands | 94.66 (44.05, 145.49) | 57.47% (7.96%, 109.51%) | 1807.71 (873.03, 2744.36) | 21.09% (-15.38%, 60.53%) | 196.64 (75.07, 313.74) | 167.83% (114.51%, 194.19%) | 2004.35 (950.47, 2997.9) | 27.97% (-9.11%, 66.55%) |
| Inner Mongolia | Absolute number,  thousands | 40.41 (15.05, 63.73) | 92.25% (31.65%, 157.32%) | 865.21 (348.83, 1328.48) | 52.51% (6.49%, 106.33%) | 81.44 (29.28, 130.82) | 220.14% (149.79%, 259.13%) | 946.66 (385.76, 1449.87) | 59.7% (13.75%, 112.06%) |
| Jiangsu | Absolute number,  thousands | 78.88 (32.11, 131.51) | 65.91% (14.37%, 118.36%) | 1403.73 (603.16, 2303.84) | 25.84% (-8.62%, 68.76%) | 281.12 (103.16, 456.47) | 200.66% (143.3%, 237.87%) | 1684.86 (709.7, 2684.28) | 39.37% (3.79%, 80.22%) |
| Jiangxi | Absolute number,  thousands | 49.82 (24.22, 77.51) | 19.56% (-17.11%, 62.05%) | 980.4 (481.78, 1500.97) | -6.28% (-33.07%, 26.99%) | 112.54 (43.71, 180.24) | 159.1% (110.05%, 188.96%) | 1092.94 (528.08, 1659.85) | 0.31% (-27.15%, 33.66%) |
| Jilin | Absolute number,  thousands | 44.39 (15, 71.46) | 43.93% (-12.74%, 95.81%) | 939.61 (349.59, 1497.43) | 15.03% (-24.66%, 57.79%) | 97.34 (34.64, 158.52) | 159.89% (96.77%, 193.09%) | 1036.95 (388.12, 1630.98) | 21.38% (-18.35%, 62.64%) |
| Liaoning | Absolute number,  thousands | 82.67 (21.62, 138.39) | 141.69% (64.26%, 224.74%) | 1690.65 (515.34, 2763.49) | 98.43% (40.58%, 169.05%) | 177.45 (57.15, 297.15) | 199.73% (136.36%, 232.1%) | 1868.09 (575.28, 3000.94) | 105.01% (48.08%, 170.86%) |
| Macao | Absolute number,  thousands | 0.43 (0.18, 0.67) | 72.46% (32.01%, 125.18%) | 8.64 (3.93, 13.31) | 58.16% (21.25%, 106.2%) | 1.86 (0.67, 3) | 339.8% (214.67%, 420.7%) | 10.5 (4.63, 15.96) | 78.46% (40.37%, 132.22%) |
| Ningxia | Absolute number,  thousands | 6.74 (1.77, 11.81) | 139.73% (37.21%, 242.41%) | 149.9 (43.47, 258.71) | 94.56% (21.76%, 178.53%) | 15.01 (4.81, 24.49) | 295.59% (205.3%, 340.94%) | 164.91 (48.15, 278.76) | 103.99% (29.4%, 185.81%) |
| Qinghai | Absolute number,  thousands | 6.83 (3.47, 10.87) | 129.95% (64.28%, 211.25%) | 155.96 (83.1, 250.77) | 81.29% (28.44%, 152.9%) | 12.76 (5.13, 20.66) | 240.42% (169.72%, 286.23%) | 168.72 (89.84, 266.35) | 87.93% (35.63%, 155.89%) |
| Shaanxi | Absolute number,  thousands | 55.26 (22.3, 88.35) | 76.36% (14.78%, 139.72%) | 1123.05 (490.08, 1748.08) | 36.96% (-5.43%, 87.3%) | 108.74 (42.48, 171) | 181.33% (126.9%, 208.23%) | 1231.78 (534.01, 1907.94) | 43.46% (-0.09%, 92.66%) |
| Shandong | Absolute number,  thousands | 130.39 (45.47, 209.58) | 83.11% (19.02%, 146.81%) | 2515.93 (917.41, 4014.46) | 47.67% (-3.76%, 101.77%) | 333.44 (120.93, 536.55) | 192.59% (130.89%, 229.11%) | 2849.37 (1050.31, 4496.82) | 56.75% (7.54%, 108.66%) |
| Shanghai | Absolute number,  thousands | 19.81 (6.98, 34.47) | 89.99% (13.3%, 165.27%) | 346.73 (133.49, 595.92) | 47.83% (-4.57%, 109.21%) | 80.38 (24.41, 134.41) | 240.03% (151.29%, 284.13%) | 427.1 (163.37, 712.05) | 65.43% (9.69%, 124.14%) |
| Shanxi | Absolute number,  thousands | 45.17 (14.5, 72.37) | 75.38% (18.77%, 139.14%) | 955.1 (326.18, 1502.73) | 41.38% (-4.74%, 94.04%) | 115.97 (40.63, 188.84) | 215.75% (150.89%, 252.94%) | 1071.07 (368.27, 1657.98) | 50.37% (4.64%, 100.57%) |
| Sichuan | Absolute number,  thousands | 96.03 (40.94, 161.5) | 27.52% (-11.04%, 71.95%) | 1901.95 (874.75, 3132.3) | -2.33% (-29.56%, 35.15%) | 248.2 (89.04, 397.3) | 95.05% (58.25%, 116.02%) | 2150.15 (964.01, 3498.11) | 3.64% (-24.37%, 39.4%) |
| Tianjin | Absolute number,  thousands | 19.28 (4.28, 33.04) | 129.92% (32.55%, 218.37%) | 377.78 (100.7, 631.23) | 88.49% (22.25%, 159.04%) | 50.74 (15, 83.52) | 271.79% (168.33%, 322.96%) | 428.51 (115.55, 699.73) | 100.18% (33.54%, 169.63%) |
| Xinjiang | Absolute number,  thousands | 25.51 (12.34, 39.7) | 68.78% (24.64%, 128.67%) | 618.55 (293.77, 953.23) | 54.39% (10.49%, 112.25%) | 64.98 (24.15, 103.85) | 310.72% (231.65%, 354.18%) | 683.53 (319.87, 1042.87) | 64.13% (20.62%, 118.64%) |
| Xizang | Absolute number,  thousands | 3.11 (1.8, 4.82) | -10.02% (-36.96%, 22.22%) | 82.43 (45.38, 127.24) | -13.34% (-42.95%, 20.3%) | 5.82 (2.72, 8.91) | 152.5% (111.71%, 184.17%) | 88.26 (51.56, 134.63) | -9.41% (-38.67%, 23.16%) |
| Yunnan | Absolute number,  thousands | 49.83 (23.56, 78.25) | 97.83% (36.54%, 166.82%) | 1095.78 (538.05, 1681.13) | 68.11% (17.08%, 130.9%) | 111.32 (42.73, 178.48) | 203.68% (146.43%, 233.87%) | 1207.1 (579.42, 1824.52) | 75.33% (25.58%, 134.54%) |
| Zhejiang | Absolute number,  thousands | 59.96 (27.46, 96.26) | 85% (29.67%, 147.83%) | 1099.52 (527.88, 1736.74) | 49.52% (5.02%, 102.56%) | 187.92 (73.64, 298.09) | 244.94% (163.99%, 285.7%) | 1287.44 (599.83, 2010.7) | 63% (17.18%, 113.08%) |
| Anhui | Age-standardized  percent | 14.9% (6.48%, 22.87%) | 10.51% (-14.19%, 25.66%) | 12.4% (5.68%, 18.76%) | 26.86% (3.18%, 43.81%) | 2.53% (1.05%, 3.86%) | 33.32% (3.99%, 49.2%) | 8.47% (3.81%, 12.7%) | 3.49% (-17.91%, 19.23%) |
| Beijing | Age-standardized  percent | 15.36% (5.14%, 24.16%) | -5.64% (-34.68%, 8.19%) | 14.26% (5.05%, 21.73%) | -4.16% (-31.48%, 7.63%) | 2.62% (0.95%, 4.05%) | 29.71% (-14.75%, 56.36%) | 8.77% (3.19%, 13.64%) | -20.72% (-43.87%, -5.64%) |
| Chongqing | Age-standardized  percent | 11.36% (5.21%, 17.5%) | 11.32% (-12.96%, 28.84%) | 9.6% (4.8%, 14.31%) | 23.42% (2.45%, 44.13%) | 2.1% (0.83%, 3.23%) | 37.88% (8.48%, 55.48%) | 6.85% (3.43%, 10.33%) | 4.15% (-15.51%, 24.62%) |
| Fujian | Age-standardized  percent | 11.89% (5.21%, 18.76%) | 6.57% (-19.66%, 23.62%) | 9.82% (4.25%, 15.2%) | 20.27% (-4.63%, 38.92%) | 2.18% (0.82%, 3.38%) | 29.93% (-8.68%, 49.79%) | 6.45% (2.86%, 10.11%) | -2.27% (-22.37%, 16.13%) |
| Gansu | Age-standardized  percent | 12.55% (4.48%, 19.81%) | 30.06% (-5.04%, 52.92%) | 10.6% (3.9%, 16.37%) | 59.34% (24.34%, 86.27%) | 1.7% (0.59%, 2.66%) | 29.67% (2.93%, 41.72%) | 7.36% (2.66%, 11.56%) | 30.02% (0.27%, 53.87%) |
| Guangdong | Age-standardized  percent | 12.46% (3.77%, 19.69%) | 9.94% (-29.89%, 27.59%) | 11.36% (3.86%, 17.39%) | 20.87% (-13.57%, 38.73%) | 2.16% (0.67%, 3.4%) | 37.82% (0.06%, 57.63%) | 7.29% (2.54%, 11.3%) | -1.03% (-31.24%, 16.46%) |
| Guangxi | Age-standardized  percent | 12.04% (4.25%, 18.53%) | 13.44% (-27.24%, 31.38%) | 10.41% (3.87%, 15.55%) | 37.95% (-4.16%, 64.13%) | 2.18% (0.73%, 3.47%) | 36.73% (-3.07%, 55.77%) | 7.28% (2.58%, 11.29%) | 18.09% (-17.07%, 41.6%) |
| Guizhou | Age-standardized  percent | 11.04% (5.43%, 16.83%) | 14.57% (-6.38%, 30.26%) | 9.89% (5.2%, 14.59%) | 54.99% (27.08%, 81.56%) | 2.16% (0.91%, 3.29%) | 33.77% (5.03%, 48.36%) | 7.29% (3.76%, 11.02%) | 29.64% (4.11%, 54.82%) |
| Hainan | Age-standardized  percent | 11.9% (3.76%, 19.07%) | 5.13% (-31.51%, 28.61%) | 10.44% (3.64%, 16.2%) | 33.81% (-7.05%, 64.95%) | 2.45% (0.76%, 3.89%) | 45.06% (2.06%, 66.7%) | 7.4% (2.54%, 11.57%) | 14.65% (-22.34%, 44.09%) |
| Hebei | Age-standardized  percent | 17.36% (8.14%, 25.52%) | -5.11% (-25.53%, 8.23%) | 15.33% (7.17%, 22.08%) | 10.67% (-11.85%, 28.08%) | 2.56% (1.12%, 3.89%) | 30.1% (-0.06%, 45.91%) | 10.8% (5%, 15.82%) | -0.87% (-22.03%, 15.91%) |
| Heilongjiang | Age-standardized  percent | 16.89% (3.89%, 26.28%) | 3.77% (-32.71%, 18.76%) | 14.99% (4.19%, 22.36%) | 11.36% (-21.84%, 26.4%) | 2.52% (0.88%, 3.9%) | 21.66% (-10.59%, 38.69%) | 10.76% (3.03%, 16.37%) | -4% (-32.51%, 10.94%) |
| Henan | Age-standardized  percent | 16.92% (6.37%, 25.82%) | 9.17% (-18.34%, 23.34%) | 15% (6.12%, 22.25%) | 42.26% (12.71%, 63.62%) | 2.46% (1%, 3.78%) | 33.46% (2.91%, 51.54%) | 10.23% (4.37%, 15.27%) | 18.39% (-9.68%, 36.87%) |
| Hong Kong | Age-standardized  percent | 10.65% (5.12%, 16.04%) | -17.52% (-27.81%, -6.83%) | 9.85% (5.01%, 14.26%) | -10.77% (-19.61%, -1.11%) | 2.17% (0.88%, 3.19%) | 33.72% (8.33%, 51.44%) | 5.55% (2.66%, 8.16%) | -25.04% (-35.19%, -14.74%) |
| Hubei | Age-standardized  percent | 13.92% (6.31%, 21.39%) | -9.64% (-35.86%, 6.05%) | 11.89% (5.82%, 17.73%) | 4.18% (-21.57%, 20.15%) | 2.13% (0.93%, 3.2%) | 22.27% (-8.49%, 37.55%) | 8.2% (3.97%, 12.32%) | -12.88% (-34.74%, 1.61%) |
| Hunan | Age-standardized  percent | 15.42% (6.58%, 22.76%) | 14.21% (-21.17%, 33.51%) | 13.12% (6.04%, 18.84%) | 36.85% (1.82%, 57.64%) | 2.19% (0.9%, 3.34%) | 35.82% (7.04%, 50.64%) | 8.79% (4.18%, 12.98%) | 10.81% (-16.56%, 29.84%) |
| Inner Mongolia | Age-standardized  percent | 16.99% (6.36%, 25.71%) | 6.89% (-21.01%, 20.45%) | 14.93% (6.14%, 21.8%) | 23.18% (-2.29%, 38.96%) | 2.48% (1.04%, 3.81%) | 27.54% (-1.82%, 42.71%) | 10.6% (4.31%, 15.83%) | 4.52% (-18.62%, 20.47%) |
| Jiangsu | Age-standardized  percent | 11.12% (4.5%, 18.03%) | 4.79% (-22.09%, 21.1%) | 9.86% (4.4%, 15.55%) | 17.59% (-4.34%, 34.5%) | 2.39% (0.94%, 3.61%) | 37.57% (6.04%, 55.15%) | 6.47% (3.01%, 10.12%) | -2.1% (-21.33%, 13.64%) |
| Jiangxi | Age-standardized  percent | 14.34% (7.16%, 21.16%) | 9.36% (-15.39%, 25.74%) | 11.82% (6.21%, 17.26%) | 25.24% (-0.76%, 44.03%) | 2.19% (0.95%, 3.32%) | 24.37% (-3.89%, 38.99%) | 8.21% (4.16%, 12.06%) | 0.83% (-24.63%, 18.52%) |
| Jilin | Age-standardized  percent | 18.73% (5.5%, 28.41%) | -0.92% (-41.02%, 14.35%) | 16.8% (5.96%, 24.5%) | 7.13% (-28.61%, 21.52%) | 2.67% (1.07%, 4.09%) | 16.41% (-13.74%, 31.46%) | 11.48% (4.14%, 17.34%) | -10.22% (-40.47%, 4.31%) |
| Liaoning | Age-standardized  percent | 15.95% (3.96%, 25.59%) | 4.68% (-26.11%, 19.95%) | 14.41% (4.45%, 22.02%) | 20.58% (-6.15%, 39.81%) | 2.66% (0.95%, 4.22%) | 30.11% (2.62%, 45.16%) | 10.25% (3.26%, 16.08%) | 9.78% (-16.08%, 28.6%) |
| Macao | Age-standardized  percent | 13.26% (5.63%, 20.1%) | -7.54% (-23.85%, 5.26%) | 12.26% (5.56%, 17.91%) | 1.31% (-15.51%, 16.88%) | 2.21% (0.87%, 3.38%) | 33.57% (-4.38%, 57.67%) | 6.81% (3.07%, 10.17%) | -19.62% (-34.25%, -5.1%) |
| Ningxia | Age-standardized  percent | 14.1% (3.64%, 22.55%) | 23.25% (-18.29%, 42.62%) | 12.15% (3.49%, 18.96%) | 57.93% (13.42%, 86.41%) | 1.91% (0.66%, 3.02%) | 26.49% (-3.38%, 41.4%) | 8.41% (2.5%, 13.46%) | 27.88% (-6.6%, 54.27%) |
| Qinghai | Age-standardized  percent | 13.14% (6.35%, 20.17%) | 28.68% (3.18%, 47.79%) | 10.79% (5.63%, 16.21%) | 55.79% (28.6%, 86.53%) | 2.07% (0.9%, 3.14%) | 35.06% (5.85%, 51.36%) | 8.37% (4.44%, 12.72%) | 36.95% (12.57%, 65.99%) |
| Shaanxi | Age-standardized  percent | 16.61% (6.71%, 25.36%) | 18.6% (-11.98%, 33.76%) | 14.26% (6.48%, 21.02%) | 48.49% (17.7%, 67.25%) | 2.2% (0.96%, 3.34%) | 24.87% (0.02%, 36.98%) | 9.76% (4.48%, 14.59%) | 19.92% (-8.75%, 39.83%) |
| Shandong | Age-standardized  percent | 15.53% (5.32%, 24.46%) | 13.81% (-22.15%, 29.84%) | 13.26% (5.02%, 20.2%) | 29.38% (-4.33%, 46.71%) | 2.42% (0.96%, 3.68%) | 33.8% (1.49%, 51.22%) | 8.72% (3.33%, 13.46%) | 7.71% (-21.78%, 26.11%) |
| Shanghai | Age-standardized  percent | 10.83% (3.66%, 17.97%) | 5.14% (-31.15%, 23.11%) | 9.21% (3.58%, 14.73%) | 5.57% (-23.93%, 24.64%) | 2.3% (0.77%, 3.62%) | 31.56% (-3.88%, 50.78%) | 5.88% (2.37%, 9.46%) | -10.06% (-36.63%, 9.25%) |
| Shanxi | Age-standardized  percent | 15.22% (5.15%, 23.96%) | 9.82% (-17.27%, 23.5%) | 13.43% (5.05%, 20.41%) | 24.41% (-3.57%, 40.45%) | 2.59% (0.93%, 4.03%) | 42.75% (10.65%, 60.61%) | 9.37% (3.48%, 14.53%) | 6.85% (-18.34%, 24.04%) |
| Sichuan | Age-standardized  percent | 10.73% (4.73%, 16.54%) | 13.08% (-13.97%, 27.74%) | 9.06% (4.4%, 13.4%) | 38.37% (13.58%, 59.31%) | 2.09% (0.79%, 3.2%) | 35.09% (5.08%, 50.58%) | 6.48% (3.18%, 9.81%) | 17.32% (-4.58%, 35.69%) |
| Tianjin | Age-standardized  percent | 14.68% (2.71%, 24.36%) | 6.45% (-39.85%, 24.03%) | 13.36% (3.27%, 20.95%) | 10.88% (-25.78%, 26.43%) | 2.71% (0.85%, 4.34%) | 40.17% (-2.9%, 60.74%) | 9.21% (2.48%, 14.61%) | -1.08% (-33.47%, 16.49%) |
| Xinjiang | Age-standardized  percent | 14.16% (7.05%, 20.66%) | 12.51% (-3.52%, 26.8%) | 11.23% (5.48%, 15.94%) | 33.97% (11.54%, 58.77%) | 2.43% (0.99%, 3.68%) | 40.89% (12.86%, 56.31%) | 8.51% (4.22%, 12.21%) | 15.41% (-4.58%, 38.19%) |
| Xizang | Age-standardized  percent | 12.26% (7.24%, 18.13%) | -4.69% (-24.31%, 11.58%) | 9.72% (5.74%, 14.09%) | 8.79% (-17.78%, 37.05%) | 1.87% (0.96%, 2.74%) | 21.11% (1.7%, 35.45%) | 7.73% (4.58%, 11.42%) | -3.79% (-27.04%, 20.33%) |
| Yunnan | Age-standardized  percent | 11.74% (5.68%, 17.46%) | 35.95% (5.44%, 56.41%) | 10.04% (5.05%, 14.46%) | 78.8% (44.24%, 109.12%) | 2% (0.81%, 3.06%) | 40.5% (11.86%, 55.98%) | 7.41% (3.7%, 10.8%) | 51.05% (21.72%, 79.81%) |
| Zhejiang | Age-standardized  percent | 12.12% (5.63%, 18.53%) | 12.45% (-10.9%, 28.11%) | 10.58% (5.28%, 16.02%) | 27.28% (2.93%, 43.58%) | 2.21% (0.92%, 3.29%) | 37.58% (2.6%, 54.18%) | 6.94% (3.45%, 10.56%) | 5.15% (-17.44%, 21.32%) |
| Anhui | Age-standardized rate,  per 100,000 people | 90.77 (36.24, 143.07) | -49.07% (-64.67%, -34.09%) | 1618.6 (681.75, 2559.19) | -54.77% (-68.07%, -40.81%) | 218.86 (84.59, 351.43) | 24.72% (-3.49%, 39.4%) | 1837.46 (762.92, 2864.58) | -51.05% (-65.13%, -37.11%) |
| Beijing | Age-standardized rate,  per 100,000 people | 77.73 (23.88, 129.06) | -52.51% (-69.35%, -35.3%) | 1382.86 (446.95, 2232.8) | -56.32% (-70.59%, -39.74%) | 227.69 (76.65, 372.12) | 25.59% (-16.76%, 49.93%) | 1610.55 (521.18, 2542.67) | -51.88% (-67.04%, -35.75%) |
| Chongqing | Age-standardized rate,  per 100,000 people | 82.49 (37.73, 135.2) | -43.97% (-60.49%, -22.06%) | 1487.44 (746.06, 2434.89) | -52.16% (-66.31%, -31.54%) | 189.29 (68.51, 311.72) | 30.41% (2.6%, 47.03%) | 1676.74 (816.11, 2666.65) | -48.48% (-62.26%, -28.22%) |
| Fujian | Age-standardized rate,  per 100,000 people | 59.15 (23.7, 97.57) | -48.93% (-64.92%, -30.76%) | 1079.75 (462.65, 1747.11) | -55.14% (-68.92%, -37.65%) | 190.17 (66.38, 309.46) | 20.92% (-13.51%, 38.92%) | 1269.92 (533.81, 2014.42) | -50.47% (-64.28%, -33.55%) |
| Gansu | Age-standardized rate,  per 100,000 people | 88.87 (30.32, 148.3) | -35.52% (-55.95%, -14.85%) | 1648.83 (577.58, 2694.42) | -41.33% (-58.77%, -20.14%) | 151.29 (47.64, 249.15) | 20.34% (-3.59%, 32.17%) | 1800.12 (635, 2905.11) | -38.69% (-56.15%, -18.53%) |
| Guangdong | Age-standardized rate,  per 100,000 people | 68.38 (20.63, 115.59) | -44.64% (-66.31%, -25.17%) | 1262.45 (424.2, 2079.29) | -49.69% (-66.68%, -31.43%) | 191.75 (52.54, 323.04) | 30.35% (-6.04%, 49.01%) | 1454.2 (476.68, 2353.33) | -45.26% (-62.7%, -27.22%) |
| Guangxi | Age-standardized rate,  per 100,000 people | 74.52 (25.48, 122.98) | -31.91% (-57.57%, -8.12%) | 1504.64 (543.14, 2442.24) | -36.26% (-58.93%, -11.96%) | 194.08 (58.9, 320.29) | 28.6% (-9.36%, 45.92%) | 1698.72 (610.13, 2699.71) | -32.37% (-55.86%, -9.41%) |
| Guizhou | Age-standardized rate,  per 100,000 people | 89.94 (43.24, 143.98) | -42.85% (-60.33%, -24.7%) | 1772.68 (912.89, 2819.21) | -47.29% (-63.72%, -27.88%) | 196.47 (76.59, 314.77) | 22.3% (-3.42%, 35.55%) | 1969.14 (983.49, 3042.02) | -44.12% (-60.03%, -25.48%) |
| Hainan | Age-standardized rate,  per 100,000 people | 78.72 (25.28, 135.58) | -38.72% (-62.49%, -13.38%) | 1514.24 (499.96, 2528.78) | -41.5% (-62.67%, -14.25%) | 218.23 (60.62, 364.94) | 38.13% (-3.18%, 58.59%) | 1732.48 (568.17, 2816.91) | -36.91% (-59.69%, -9.92%) |
| Hebei | Age-standardized rate,  per 100,000 people | 130 (57.23, 205.42) | -33.1% (-54.01%, -10.45%) | 2461.47 (1103.67, 3889.73) | -35.02% (-54.78%, -12.91%) | 226.38 (87.49, 360.82) | 26.94% (-1.84%, 41.89%) | 2687.85 (1195.28, 4200.89) | -32.23% (-51.62%, -10.02%) |
| Heilongjiang | Age-standardized rate,  per 100,000 people | 133.55 (29.48, 218.8) | -44.42% (-64.84%, -27.35%) | 2620.95 (701.1, 4104.82) | -48.38% (-65.44%, -32.84%) | 225.81 (71.41, 365.86) | 17.45% (-13.42%, 34.32%) | 2846.76 (785.53, 4448.94) | -45.98% (-63.39%, -30.69%) |
| Henan | Age-standardized rate,  per 100,000 people | 113.08 (40.88, 185.23) | -32.87% (-54.21%, -14.73%) | 2141.03 (842.26, 3387.79) | -37.26% (-57.44%, -19.07%) | 215.62 (79.64, 345.72) | 29.13% (-0.39%, 46.3%) | 2356.65 (926.73, 3699.85) | -34.16% (-54.55%, -16.65%) |
| Hong Kong | Age-standardized rate,  per 100,000 people | 32.53 (14.21, 50.11) | -55.62% (-66.41%, -42.05%) | 650.46 (301.79, 993.46) | -55.89% (-66.57%, -41.46%) | 182.76 (67.81, 287.59) | 33.69% (7.5%, 51.1%) | 833.22 (376.91, 1251.68) | -48.29% (-58.8%, -35.27%) |
| Hubei | Age-standardized rate,  per 100,000 people | 98.04 (43.11, 155.97) | -53.4% (-68.33%, -39.59%) | 1729.82 (793.86, 2777.01) | -57.62% (-70.68%, -44.69%) | 189.18 (73.96, 302.8) | 16.09% (-13.9%, 30.72%) | 1919 (864.95, 3011.83) | -54.79% (-68.37%, -42.02%) |
| Hunan | Age-standardized rate,  per 100,000 people | 100.34 (46.11, 154.92) | -44.44% (-63%, -27.36%) | 1809.68 (858.06, 2741.92) | -49.2% (-64.73%, -33.24%) | 198.44 (75.64, 316.58) | 26.41% (-1.13%, 39.58%) | 2008.12 (940.59, 2999.59) | -46.01% (-62.15%, -30.52%) |
| Inner Mongolia | Age-standardized rate,  per 100,000 people | 130.75 (45.52, 210.12) | -43.25% (-60.87%, -26.78%) | 2440.94 (925.88, 3799.45) | -47.15% (-63.13%, -29.54%) | 216.77 (76.39, 349.01) | 21.04% (-6.66%, 35.71%) | 2657.71 (1004.86, 4112.23) | -44.6% (-60.25%, -27.93%) |
| Jiangsu | Age-standardized rate,  per 100,000 people | 60.21 (24.12, 100.52) | -44.55% (-61.89%, -27.59%) | 1028.57 (437.49, 1686.07) | -50.88% (-64.55%, -34.71%) | 207.81 (75.4, 338.87) | 31.09% (0.59%, 47.67%) | 1236.38 (519.77, 1969.4) | -45.11% (-58.39%, -29.69%) |
| Jiangxi | Age-standardized rate,  per 100,000 people | 94.29 (44.13, 147.04) | -56.51% (-69.9%, -42.3%) | 1692.56 (812.42, 2593.93) | -61.86% (-73.04%, -48.64%) | 188.18 (73.29, 302.67) | 12.02% (-12.61%, 25.77%) | 1880.73 (895.35, 2865.31) | -59.16% (-71.22%, -45.78%) |
| Jilin | Age-standardized rate,  per 100,000 people | 132.65 (39.19, 216.5) | -49.17% (-71.94%, -32.52%) | 2453.66 (837.29, 3900.49) | -53.56% (-72.11%, -37.5%) | 235.92 (82.41, 385.22) | 10.32% (-18.56%, 25.31%) | 2689.59 (929.63, 4229.77) | -51.08% (-70.43%, -35.61%) |
| Liaoning | Age-standardized rate,  per 100,000 people | 120.01 (28.66, 200.83) | -20.13% (-46.07%, 5.21%) | 2275.82 (655.36, 3741.32) | -22.98% (-44.31%, 3.59%) | 230.12 (71.57, 382.64) | 27.13% (-0.55%, 42.2%) | 2505.94 (731.64, 4052.4) | -20.09% (-41.48%, 4.63%) |
| Macao | Age-standardized rate,  per 100,000 people | 45.47 (18.25, 71.91) | -50.63% (-62.61%, -35.64%) | 883.46 (389.79, 1360.19) | -55.33% (-65.84%, -41.65%) | 190.46 (67.47, 306.63) | 31.56% (-6.18%, 54.81%) | 1073.92 (459.12, 1640.64) | -49.41% (-60.63%, -34.67%) |
| Ningxia | Age-standardized rate,  per 100,000 people | 99.48 (23.05, 175.21) | -36.93% (-62.96%, -14.82%) | 1864.67 (496.99, 3237.55) | -42.37% (-65.98%, -19.91%) | 168.96 (53.95, 275.27) | 20.44% (-7.62%, 33.98%) | 2033.62 (549.42, 3483.5) | -39.76% (-63.11%, -17.74%) |
| Qinghai | Age-standardized rate,  per 100,000 people | 129.11 (59.82, 204.93) | -23.91% (-45.48%, -1.16%) | 2447.05 (1259.12, 3867.92) | -32.45% (-51.55%, -9.22%) | 180.13 (71.62, 290.42) | 26.5% (-1.07%, 41.57%) | 2627.18 (1348.52, 4090.37) | -30.22% (-49.11%, -7.83%) |
| Shaanxi | Age-standardized rate,  per 100,000 people | 111.97 (42.8, 180.48) | -40.79% (-60.98%, -21.11%) | 2035.19 (855.91, 3182.49) | -46.87% (-63.74%, -28.12%) | 186.4 (71.87, 293.58) | 16.06% (-6.1%, 27.78%) | 2221.59 (929.8, 3473.27) | -44.34% (-61.42%, -26.17%) |
| Shandong | Age-standardized rate,  per 100,000 people | 89.29 (29.38, 144.1) | -35.89% (-58.5%, -15.9%) | 1623.66 (562.14, 2590.7) | -40.49% (-60.05%, -19.31%) | 213.67 (77.42, 341.1) | 29.5% (-2.23%, 46.42%) | 1837.34 (642.84, 2899.33) | -36.5% (-56.04%, -16.23%) |
| Shanghai | Age-standardized rate,  per 100,000 people | 49.7 (17.12, 86.61) | -44.93% (-67.05%, -24.78%) | 853.11 (325.57, 1465.6) | -50.11% (-67.52%, -30.27%) | 198.99 (59.83, 331.65) | 27.42% (-7.35%, 46.63%) | 1052.11 (397.04, 1751.89) | -43.62% (-62.2%, -24.28%) |
| Shanxi | Age-standardized rate,  per 100,000 people | 103.81 (31.02, 167.99) | -37.32% (-58.61%, -18.54%) | 1965.15 (645.82, 3104.07) | -42.46% (-60.99%, -21.98%) | 227.93 (78.78, 372.28) | 36.11% (5.27%, 52.53%) | 2193.08 (726.64, 3403.45) | -38.79% (-58.04%, -18.86%) |
| Sichuan | Age-standardized rate,  per 100,000 people | 72 (30.17, 121.13) | -36.74% (-55.47%, -16.55%) | 1389.73 (630.02, 2277.53) | -42.23% (-58.52%, -21.94%) | 187.83 (65.64, 302.69) | 29.21% (1.04%, 43.18%) | 1577.56 (719.01, 2537.68) | -38.16% (-54.57%, -18.17%) |
| Tianjin | Age-standardized rate,  per 100,000 people | 101.11 (19.16, 175.07) | -31.82% (-61.72%, -6.78%) | 1822.67 (441.5, 3055.84) | -35.93% (-60.66%, -12.51%) | 236.92 (68.75, 393.97) | 39.63% (-2.77%, 60.62%) | 2059.59 (509.52, 3390.86) | -31.68% (-55.61%, -8.63%) |
| Xinjiang | Age-standardized rate,  per 100,000 people | 110.74 (53.01, 171.79) | -47.35% (-60.69%, -29.84%) | 2275.62 (1110.07, 3525.67) | -49.7% (-63.59%, -31.36%) | 221.52 (85.29, 355.3) | 32.7% (6.94%, 46.98%) | 2497.15 (1201.41, 3777.65) | -46.77% (-60.24%, -29.22%) |
| Xizang | Age-standardized rate,  per 100,000 people | 118.08 (68.3, 181.17) | -57.3% (-69.79%, -42.64%) | 2576.88 (1514.43, 3990.78) | -60.07% (-72.2%, -45.16%) | 167.55 (76.99, 261.82) | 9.45% (-7.66%, 21.83%) | 2744.43 (1632.85, 4211.65) | -58.46% (-70.43%, -44.09%) |
| Yunnan | Age-standardized rate,  per 100,000 people | 94.73 (43.8, 148.44) | -27.36% (-48.54%, -4.71%) | 1872.68 (896.75, 2873.01) | -31.32% (-51.72%, -7.36%) | 181.65 (69.74, 292.86) | 28.73% (2.34%, 42.09%) | 2054.33 (967.78, 3115.4) | -28.36% (-48.2%, -5.41%) |
| Zhejiang | Age-standardized rate,  per 100,000 people | 70.52 (31.13, 113.91) | -40.67% (-58.95%, -22.41%) | 1190.47 (564.66, 1887.03) | -47.01% (-62.7%, -29.48%) | 192.73 (74.99, 305.5) | 28.86% (-3.26%, 45.3%) | 1383.2 (638.87, 2163.59) | -42.27% (-59.33%, -25.68%) |
| **Cardiovascular diseases** | | | | | | | | | |
| Anhui | Absolute number,  thousands | 67.93 (29.31, 103.92) | 61.06% (6.24%, 113.36%) | 1218.47 (554.87, 1845.27) | 17.07% (-21.92%, 57.42%) | 90.82 (39.74, 142.78) | 116.65% (73.44%, 146.09%) | 1309.3 (611.68, 1964.52) | 20.92% (-17.94%, 59.43%) |
| Beijing | Absolute number,  thousands | 19.12 (6.08, 31.12) | 93.36% (15.13%, 167.27%) | 356.82 (121.69, 564.38) | 52.79% (-4.25%, 114.89%) | 30.65 (11.86, 49.38) | 175.21% (108.37%, 210.7%) | 387.46 (137.55, 603.05) | 58.36% (2.37%, 118.71%) |
| Chongqing | Absolute number,  thousands | 34.09 (16.23, 56.42) | 206.55% (107.36%, 339.05%) | 620.06 (316.23, 1005.87) | 125.25% (54.36%, 225%) | 43.73 (18.37, 70.49) | 327.18% (235.33%, 392.49%) | 663.79 (340.45, 1063.97) | 132.5% (62.89%, 229.91%) |
| Fujian | Absolute number,  thousands | 22.26 (9.3, 35.44) | 62.66% (4.21%, 128.37%) | 416.7 (195.74, 636.3) | 26.13% (-16.52%, 81.18%) | 41.88 (17.88, 67.29) | 125.75% (80.43%, 154.08%) | 458.58 (220.07, 690.74) | 31.42% (-11.08%, 84.62%) |
| Gansu | Absolute number,  thousands | 22.13 (7.73, 34.78) | 108.11% (37.26%, 190.81%) | 456.45 (169.03, 701.53) | 59.62% (7.67%, 120.11%) | 25.28 (8.79, 42.94) | 151.69% (91.65%, 197.06%) | 481.73 (179.7, 738.68) | 62.75% (11.47%, 121.16%) |
| Guangdong | Absolute number,  thousands | 65.81 (19.99, 108.34) | 75.96% (-5.91%, 145.52%) | 1309.18 (447.34, 2068.35) | 54.04% (-5.68%, 117.97%) | 89.76 (34.34, 145.99) | 161.12% (101.31%, 191.61%) | 1398.94 (483.35, 2211.13) | 58.21% (-1.8%, 120.58%) |
| Guangxi | Absolute number,  thousands | 39.92 (13.91, 65.12) | 75.93% (3.34%, 143.13%) | 799.66 (292.96, 1278.4) | 45.52% (-10.12%, 104.89%) | 48.02 (17.1, 79.75) | 100.57% (46.82%, 135.35%) | 847.68 (313.02, 1354.81) | 47.82% (-7.58%, 105.08%) |
| Guizhou | Absolute number,  thousands | 32.71 (15.9, 53.15) | 47.17% (-0.13%, 99.19%) | 680.34 (359.98, 1084.02) | 21.41% (-20.17%, 69.66%) | 45.17 (18.44, 72.01) | 126.54% (78.08%, 165.98%) | 725.51 (381.93, 1158.56) | 25.03% (-15.43%, 73.83%) |
| Hainan | Absolute number,  thousands | 7.56 (2.41, 12.81) | 97.34% (14.71%, 191.33%) | 146.22 (50.55, 237.97) | 68.17% (2.42%, 152.26%) | 9.3 (3.19, 15.19) | 144.89% (82.17%, 177.05%) | 155.52 (53.73, 251.32) | 71.38% (7.89%, 152.99%) |
| Hebei | Absolute number,  thousands | 111.24 (51.93, 174.62) | 89.51% (26.59%, 153.96%) | 2283.94 (1084.01, 3550.67) | 64.22% (11.13%, 125.06%) | 132.37 (57.83, 211.3) | 135.48% (88.17%, 165.77%) | 2416.31 (1155.25, 3724.34) | 66.98% (13.92%, 125.53%) |
| Heilongjiang | Absolute number,  thousands | 57.31 (14.3, 91.74) | 74.74% (1.46%, 133.71%) | 1234.11 (354.82, 1912.85) | 35.82% (-10.78%, 82.91%) | 58.96 (19.69, 98.13) | 118.75% (58.6%, 157.46%) | 1293.07 (384.25, 1990.53) | 38.21% (-8.74%, 84.54%) |
| Henan | Absolute number,  thousands | 129.89 (51.28, 204.52) | 83.95% (18.53%, 142.63%) | 2559.09 (1054.12, 3940.14) | 52.12% (-1.09%, 102.41%) | 150.35 (63.01, 242.52) | 118.55% (70.28%, 149.46%) | 2709.44 (1137.52, 4132.15) | 54.73% (2.08%, 103.1%) |
| Hong Kong | Absolute number,  thousands | 3.78 (1.71, 5.86) | 39.32% (0.65%, 86.22%) | 63.53 (31.22, 95.38) | 10.68% (-17.87%, 50.55%) | 10.91 (5.03, 17.17) | 156.64% (110.18%, 198.96%) | 74.45 (37.51, 110.01) | 20.74% (-6.76%, 59.26%) |
| Hubei | Absolute number,  thousands | 66.39 (30.09, 102.8) | 30.33% (-15.39%, 70.92%) | 1258.83 (582.95, 1948.71) | 3.49% (-30.56%, 37.26%) | 78.3 (33.31, 125.32) | 107.22% (59.04%, 138.45%) | 1337.13 (611.92, 2052.03) | 6.61% (-27.93%, 39.91%) |
| Hunan | Absolute number,  thousands | 81.72 (38.27, 125) | 58.78% (4.94%, 114.12%) | 1515.86 (741.35, 2324.29) | 21.69% (-16.4%, 60.37%) | 87.42 (37.64, 137.7) | 130.78% (83.36%, 166.84%) | 1603.28 (787.46, 2457.2) | 24.91% (-13.06%, 62.37%) |
| Inner Mongolia | Absolute number,  thousands | 36.16 (13.53, 57.2) | 99% (29.97%, 167.25%) | 764.66 (304.7, 1171.49) | 58.17% (7.42%, 116.45%) | 41.68 (17.92, 66.06) | 159.03% (104.87%, 196%) | 806.35 (323.87, 1232.8) | 61.42% (12.28%, 117.75%) |
| Jiangsu | Absolute number,  thousands | 63.44 (26.6, 102.17) | 79.91% (13.89%, 145.75%) | 1078.29 (510.29, 1685.5) | 36.41% (-7.42%, 87.83%) | 124.92 (53.25, 204.29) | 141.48% (94.03%, 178.29%) | 1203.2 (571.03, 1866.21) | 42.86% (-0.71%, 91.93%) |
| Jiangxi | Absolute number,  thousands | 42.48 (20.96, 64.27) | 21.91% (-18.48%, 66.38%) | 811.64 (423.46, 1207.47) | -4.88% (-34.21%, 29.61%) | 51.52 (24.88, 81.24) | 106.29% (70.19%, 131.95%) | 863.16 (453.38, 1273.32) | -1.72% (-31.04%, 32.47%) |
| Jilin | Absolute number,  thousands | 39.44 (12.79, 63.67) | 44.7% (-17.23%, 98%) | 820.69 (296.88, 1301.53) | 15.47% (-26.99%, 60.21%) | 51.27 (20.31, 81.94) | 114.48% (61.81%, 147.37%) | 871.96 (322.94, 1382.99) | 18.69% (-22.9%, 62.62%) |
| Liaoning | Absolute number,  thousands | 70.34 (17.01, 117.8) | 145.24% (59.05%, 236.22%) | 1411.66 (412.08, 2304.87) | 100.73% (36.15%, 179.34%) | 83.97 (29.83, 141.8) | 150.8% (86.22%, 189.46%) | 1495.63 (443.57, 2412.97) | 103.01% (39.22%, 177.49%) |
| Macao | Absolute number,  thousands | 0.32 (0.13, 0.5) | 64.2% (23.76%, 114.9%) | 6.2 (2.86, 9.47) | 48.88% (10.5%, 98.52%) | 0.9 (0.39, 1.41) | 230.15% (168.32%, 288.1%) | 7.1 (3.31, 10.69) | 59.97% (22.36%, 108.59%) |
| Ningxia | Absolute number,  thousands | 5.81 (1.53, 9.63) | 150.25% (40.74%, 261.89%) | 127.27 (37.77, 203.61) | 102.9% (19.37%, 198.26%) | 7.37 (2.54, 12.12) | 231.03% (152.23%, 280.39%) | 134.64 (41.22, 214.56) | 107.29% (24.11%, 200.86%) |
| Qinghai | Absolute number,  thousands | 5.75 (2.99, 8.87) | 139.81% (64.37%, 232.77%) | 128.51 (69.35, 198.45) | 88.44% (30.64%, 168.44%) | 5.82 (2.79, 9) | 167.82% (117.49%, 207.91%) | 134.33 (73.22, 206.73) | 90.89% (33.82%, 168.99%) |
| Shaanxi | Absolute number,  thousands | 49.17 (19.92, 76.57) | 85.55% (18.28%, 159.34%) | 983.1 (434.57, 1493.18) | 44.12% (-4.94%, 100.28%) | 60.85 (27.02, 94.95) | 153.83% (102.97%, 190.36%) | 1043.95 (459.37, 1581.84) | 47.84% (-0.67%, 102.93%) |
| Shandong | Absolute number,  thousands | 114.12 (40.82, 179.84) | 93.26% (18.66%, 162.29%) | 2144.59 (837.15, 3315.77) | 56.03% (-0.02%, 114.56%) | 165.17 (66.74, 267.44) | 132.31% (81.81%, 163.07%) | 2309.77 (916.33, 3538.21) | 59.78% (5.23%, 116%) |
| Shanghai | Absolute number,  thousands | 14.15 (4.56, 24.66) | 87.99% (1.14%, 167.45%) | 231.13 (88.57, 388.39) | 43.45% (-17.98%, 106.25%) | 29.55 (10.95, 48.88) | 149.7% (92.16%, 184.49%) | 260.68 (102.04, 435.53) | 50.72% (-10.67%, 111.02%) |
| Shanxi | Absolute number,  thousands | 39.09 (12.7, 61.08) | 85.31% (20.34%, 152.42%) | 814.71 (286.26, 1258.73) | 49.75% (-3.12%, 107.77%) | 53.1 (20.24, 85.76) | 153.09% (105.55%, 188.94%) | 867.81 (308.84, 1320.63) | 53.59% (1.44%, 109.82%) |
| Sichuan | Absolute number,  thousands | 76.95 (33.7, 125.8) | 36.12% (-9.13%, 83.57%) | 1471.47 (716.57, 2328.06) | 5.11% (-26.62%, 43.09%) | 107.87 (46.26, 172.89) | 65.84% (29.24%, 92.49%) | 1579.34 (776.54, 2481.09) | 7.8% (-23.1%, 44.83%) |
| Tianjin | Absolute number,  thousands | 16.71 (3.27, 28.61) | 130.01% (19.24%, 225.46%) | 321.02 (78.9, 526.5) | 87.38% (17.45%, 158.44%) | 22.37 (6.5, 38.16) | 174.78% (89.99%, 220.63%) | 343.39 (86.28, 561.47) | 91.34% (20.33%, 160.4%) |
| Xinjiang | Absolute number,  thousands | 22.16 (11.1, 33.99) | 70.5% (25.28%, 133.14%) | 527.93 (262.43, 791.42) | 56% (10.31%, 116.04%) | 26.21 (12.47, 40.79) | 217.69% (175.51%, 251.62%) | 554.15 (278.46, 829.04) | 59.85% (14.82%, 118.37%) |
| Xizang | Absolute number,  thousands | 2.79 (1.54, 4.38) | -10.41% (-39.84%, 23.81%) | 73.07 (38.04, 114.5) | -13.85% (-46.52%, 20.92%) | 3.01 (1.62, 4.6) | 98.91% (61.67%, 129.63%) | 76.09 (40.3, 118.63) | -11.87% (-44.58%, 22.91%) |
| Yunnan | Absolute number,  thousands | 42.44 (20.25, 66.13) | 101.91% (39.44%, 172.95%) | 914.67 (463.9, 1392.37) | 71.91% (18.55%, 135.97%) | 52.32 (23.06, 83.49) | 149.23% (106.84%, 183.23%) | 966.99 (490.83, 1460.17) | 74.84% (22.74%, 136.6%) |
| Zhejiang | Absolute number,  thousands | 46.69 (21.98, 72.02) | 88.58% (23.88%, 155.69%) | 803.25 (413.01, 1209.4) | 50.07% (2.2%, 105.85%) | 82.19 (39.04, 125.99) | 157% (115.02%, 184.72%) | 885.45 (454.28, 1320.75) | 56.1% (10.27%, 109.29%) |
| Anhui | Age-standardized  percent | 29.23% (12.56%, 42.78%) | -12.83% (-36.04%, -1.05%) | 31.08% (14.39%, 43.83%) | -11.09% (-30.48%, -1.23%) | 23.59% (11.18%, 34.08%) | -14.09% (-30.37%, -5.48%) | 30.43% (14.24%, 42.87%) | -12.13% (-31.28%, -2.7%) |
| Beijing | Age-standardized  percent | 28.22% (9.42%, 44.15%) | -8.11% (-39.76%, 6.43%) | 30.62% (11.46%, 45.51%) | -7.55% (-37.11%, 3.98%) | 22.96% (9.51%, 34.56%) | -12.13% (-32.16%, -3.45%) | 29.86% (11.48%, 44.42%) | -8.84% (-36.97%, 2.11%) |
| Chongqing | Age-standardized  percent | 25.49% (11.77%, 38.39%) | -11.25% (-32.92%, 2.04%) | 26.95% (13.83%, 39.11%) | -9.35% (-24.77%, 2.22%) | 20.73% (9.37%, 31.24%) | -12.49% (-27.47%, -2.39%) | 26.44% (13.47%, 38.41%) | -10.3% (-25.47%, 0.76%) |
| Fujian | Age-standardized  percent | 26.18% (10.88%, 39.53%) | -14.58% (-41.81%, -0.86%) | 28.1% (12.9%, 40.71%) | -12.36% (-35.74%, -0.81%) | 23.06% (10.41%, 34.46%) | -13.84% (-32.15%, -4.93%) | 27.58% (12.59%, 39.92%) | -13.16% (-35.62%, -1.86%) |
| Gansu | Age-standardized  percent | 25.09% (8.79%, 38.44%) | -8.33% (-36.81%, 5.4%) | 26.6% (10.32%, 39.34%) | -5.09% (-28.77%, 7.74%) | 17.81% (7.14%, 28.04%) | -13% (-32.28%, -1.54%) | 25.98% (10.13%, 38.37%) | -6.3% (-29.05%, 6.18%) |
| Guangdong | Age-standardized  percent | 25.08% (7.6%, 39.6%) | -10.66% (-46.22%, 4.3%) | 27.38% (9.41%, 40.82%) | -8.38% (-37.87%, 4.04%) | 20.85% (8.54%, 31.54%) | -12.12% (-33.18%, -2.21%) | 26.87% (9.27%, 40.35%) | -9.25% (-37.63%, 2.63%) |
| Guangxi | Age-standardized  percent | 24.73% (8.94%, 37.62%) | -16.14% (-51.48%, -3.2%) | 26.6% (10.28%, 39.12%) | -12.76% (-41.66%, -0.36%) | 17.96% (6.94%, 28.54%) | -20.39% (-41.22%, -8.81%) | 25.9% (10.09%, 38.22%) | -13.85% (-42.27%, -1.74%) |
| Guizhou | Age-standardized  percent | 24.1% (11.44%, 36.23%) | -16.35% (-33.9%, -5.55%) | 26.1% (13.85%, 38.03%) | -12.48% (-26.62%, -2.27%) | 21.39% (9.68%, 32.18%) | -16.9% (-34.56%, -6.65%) | 25.76% (13.57%, 37.55%) | -13.19% (-26.98%, -3.19%) |
| Hainan | Age-standardized  percent | 24.05% (7.8%, 37.86%) | -11.42% (-46.9%, 5.26%) | 26.48% (9.27%, 39.73%) | -6.72% (-38.94%, 8.29%) | 18.57% (7.1%, 29.13%) | -14.68% (-36.09%, -4.4%) | 25.83% (9.02%, 38.86%) | -7.93% (-39.18%, 6.41%) |
| Hebei | Age-standardized  percent | 30.4% (14.14%, 44.08%) | -14.94% (-33.06%, -3.63%) | 32.69% (15.72%, 46.24%) | -11.46% (-30.16%, -1.96%) | 23.17% (10.66%, 34.53%) | -13.35% (-31.42%, -4.54%) | 32% (15.59%, 45.45%) | -12.15% (-30.47%, -2.63%) |
| Heilongjiang | Age-standardized  percent | 28.36% (6.31%, 44.46%) | -6.07% (-42.08%, 8.27%) | 30.78% (8.82%, 45.67%) | -4.8% (-35.22%, 7.29%) | 20.36% (7.55%, 31.32%) | -14.12% (-34.73%, -4.48%) | 30.11% (9.02%, 44.65%) | -5.98% (-34.76%, 5.31%) |
| Henan | Age-standardized  percent | 29.96% (11.54%, 45.04%) | -12.84% (-39%, -1.63%) | 32.39% (13.76%, 46.29%) | -9.52% (-31.5%, 0.13%) | 22.94% (10.27%, 34.01%) | -14.42% (-32.07%, -5.04%) | 31.68% (13.52%, 45.34%) | -10.41% (-31.73%, -1.25%) |
| Hong Kong | Age-standardized  percent | 29.25% (15.12%, 40.95%) | -8.37% (-16.54%, 3.85%) | 30.74% (17.23%, 41.76%) | -7.04% (-13.37%, 4.74%) | 22.73% (11%, 32.7%) | -6.51% (-20.9%, 4.17%) | 29.15% (16%, 40.08%) | -9.71% (-15.99%, 0.51%) |
| Hubei | Age-standardized  percent | 27.27% (12.3%, 40.46%) | -20.07% (-43.86%, -5.99%) | 28.87% (14.11%, 41.04%) | -18.23% (-39.02%, -6.51%) | 21.16% (9.73%, 31.51%) | -19.03% (-36.96%, -9.04%) | 28.3% (13.79%, 40.31%) | -19.09% (-39.21%, -7.59%) |
| Hunan | Age-standardized  percent | 29.84% (12.67%, 43.61%) | -11.52% (-39.33%, 5.29%) | 31.51% (14.53%, 44.81%) | -7.31% (-29.3%, 6.71%) | 21.82% (10.51%, 31.8%) | -14.78% (-31.38%, -5.51%) | 30.8% (14.43%, 43.74%) | -8.61% (-30.21%, 5.34%) |
| Inner Mongolia | Age-standardized  percent | 29.91% (11%, 44.71%) | -8.82% (-35.7%, 2.34%) | 32.19% (13.26%, 46.34%) | -6.86% (-27.66%, 2.01%) | 23.13% (10.61%, 34.36%) | -12.09% (-28.43%, -3.62%) | 31.59% (13.04%, 45.43%) | -7.79% (-28.02%, 0.89%) |
| Jiangsu | Age-standardized  percent | 24.09% (9.38%, 37.4%) | -17.27% (-45.71%, -3.66%) | 26.26% (11.95%, 38.6%) | -14.29% (-35.21%, -3.07%) | 22.99% (10.17%, 34.28%) | -14.12% (-30.91%, -4.3%) | 25.89% (11.77%, 38.08%) | -14.82% (-35.15%, -4.16%) |
| Jiangxi | Age-standardized  percent | 29.96% (14.89%, 42.91%) | -15.36% (-35.69%, -2.52%) | 31.49% (16.43%, 43.48%) | -13.26% (-30.52%, -2.55%) | 22.63% (11.25%, 32.83%) | -16.31% (-29.93%, -8.15%) | 30.8% (16.04%, 42.57%) | -14.39% (-31.56%, -4.06%) |
| Jilin | Age-standardized  percent | 30.45% (8.88%, 45.97%) | -12.57% (-50.08%, 1.66%) | 32.64% (11.43%, 47.23%) | -11.21% (-42.08%, 0.25%) | 22.49% (9.55%, 33.82%) | -16.27% (-34.09%, -7.15%) | 31.87% (11.32%, 46.13%) | -12.37% (-41.87%, -1.2%) |
| Liaoning | Age-standardized  percent | 26.49% (6.24%, 42.61%) | -5.73% (-36.78%, 6.67%) | 29.18% (8.74%, 44.03%) | -2.7% (-27.62%, 8.95%) | 20.37% (7.72%, 31.82%) | -6.94% (-25.64%, 2.72%) | 28.53% (8.67%, 43%) | -3.41% (-27.3%, 8.2%) |
| Macao | Age-standardized  percent | 32.05% (13.54%, 45.93%) | -3.24% (-21.36%, 11.67%) | 33.86% (16.06%, 46.71%) | -0.02% (-17.1%, 15.79%) | 20.38% (10%, 29.54%) | -6.4% (-25.29%, 5.54%) | 31.23% (14.84%, 43.42%) | -4.81% (-21.01%, 8.55%) |
| Ningxia | Age-standardized  percent | 26.91% (7.21%, 42.18%) | -5.1% (-39.28%, 7.2%) | 29.63% (8.77%, 43.9%) | -1.46% (-32.46%, 9.82%) | 19.38% (6.98%, 30.2%) | -10.78% (-31.28%, -1.24%) | 28.85% (8.9%, 42.77%) | -2.91% (-32.78%, 8.13%) |
| Qinghai | Age-standardized  percent | 27.98% (13.46%, 40.96%) | -10.13% (-30.48%, 0.34%) | 29.4% (15.85%, 41.46%) | -7.96% (-23.15%, 0.87%) | 21.58% (10.77%, 32.01%) | -13.4% (-28.09%, -3.64%) | 28.98% (15.7%, 40.95%) | -8.55% (-23.26%, 0.23%) |
| Shaanxi | Age-standardized  percent | 29.17% (12.07%, 43.43%) | -12.25% (-36.69%, -1.04%) | 31.29% (14.82%, 44.54%) | -8.65% (-29.71%, 0.56%) | 22.8% (10.82%, 33.72%) | -13.05% (-28.15%, -3.82%) | 30.67% (14.52%, 43.61%) | -9.63% (-29.69%, -0.89%) |
| Shandong | Age-standardized  percent | 29.05% (9.79%, 44.15%) | -9% (-41.49%, 4.64%) | 31.39% (12.05%, 45.24%) | -7.09% (-34.22%, 3.55%) | 22.62% (10.13%, 33.83%) | -11.78% (-27.66%, -3.97%) | 30.58% (11.8%, 44.2%) | -8.21% (-34.22%, 2%) |
| Shanghai | Age-standardized  percent | 23.24% (7.32%, 37.67%) | -5.6% (-41.96%, 13.5%) | 25.18% (9.38%, 38.49%) | -6.24% (-35.95%, 8.41%) | 21.27% (8.25%, 32.29%) | -8.84% (-28.82%, 1.68%) | 24.68% (9.38%, 37.59%) | -7.25% (-35.9%, 6.62%) |
| Shanxi | Age-standardized  percent | 27.33% (9.09%, 41.8%) | -5.89% (-33.4%, 6.3%) | 30.25% (11.41%, 43.92%) | -1.81% (-25.71%, 9.36%) | 21.14% (8.69%, 31.86%) | -7.63% (-23.04%, 2.77%) | 29.52% (11.16%, 42.85%) | -3.05% (-25.53%, 7.57%) |
| Sichuan | Age-standardized  percent | 25.5% (11.6%, 38.2%) | -11.23% (-35.12%, 0.69%) | 27.28% (14%, 39.19%) | -8.19% (-25.81%, 2.22%) | 19.54% (8.82%, 29.34%) | -15.55% (-31.43%, -5.32%) | 26.57% (13.51%, 38.29%) | -9.51% (-26.16%, 0.63%) |
| Tianjin | Age-standardized  percent | 24.7% (3.98%, 41.14%) | -8.71% (-54.16%, 6.34%) | 27.04% (6.18%, 42.03%) | -7.93% (-43.28%, 4.43%) | 19.81% (6.27%, 31.27%) | -10.59% (-36.77%, -0.02%) | 26.45% (6.28%, 41%) | -8.75% (-42.28%, 3.28%) |
| Xinjiang | Age-standardized  percent | 33.58% (16.7%, 47.41%) | -6.27% (-20.07%, 4.04%) | 35.37% (17.28%, 47.88%) | -3.14% (-14.42%, 6.88%) | 23.47% (12.17%, 33.91%) | -7.19% (-19.37%, 1.29%) | 34.56% (17.01%, 46.88%) | -4.4% (-15.46%, 5.22%) |
| Xizang | Age-standardized  percent | 27.89% (15.82%, 40.61%) | -17.52% (-35.77%, -3.67%) | 28.57% (16.25%, 41.26%) | -16.61% (-38.34%, -1.74%) | 22.55% (13.05%, 33.04%) | -14.78% (-29.48%, -4.76%) | 28.28% (16.24%, 40.79%) | -17.05% (-38.36%, -2.38%) |
| Yunnan | Age-standardized  percent | 27.09% (12.76%, 39.64%) | -6.85% (-29.05%, 5%) | 29.05% (14.85%, 40.73%) | -2.08% (-18.48%, 9.01%) | 21.27% (10.09%, 31.45%) | -11.8% (-25.72%, -1.72%) | 28.51% (14.64%, 40.23%) | -3.12% (-19.64%, 7.68%) |
| Zhejiang | Age-standardized  percent | 27.7% (12.8%, 41.15%) | -12.61% (-35.97%, -1.29%) | 29.64% (15%, 42.48%) | -11.04% (-31.96%, -1.13%) | 25.84% (13.18%, 37%) | -11.46% (-24.14%, -4.44%) | 29.26% (14.8%, 41.94%) | -11.54% (-31.9%, -1.94%) |
| Anhui | Age-standardized rate,  per 100,000 people | 78.51 (31.52, 119.86) | -46.6% (-65.34%, -30.02%) | 1345.76 (590.3, 2047.1) | -52.21% (-67.82%, -37.34%) | 96.16 (42.62, 151.29) | -5.07% (-23.12%, 5.79%) | 1441.92 (648.89, 2161.83) | -50.57% (-66.33%, -36.37%) |
| Beijing | Age-standardized rate,  per 100,000 people | 66.06 (20.2, 108.98) | -53.85% (-71.75%, -37.35%) | 1143.54 (377.47, 1808.02) | -57.73% (-73.43%, -42.09%) | 92.48 (35.24, 149.34) | -19.57% (-37.98%, -10.45%) | 1236.02 (424.4, 1938.5) | -56.17% (-71.46%, -41.02%) |
| Chongqing | Age-standardized rate,  per 100,000 people | 68.89 (30.88, 114.78) | -41.62% (-60.31%, -18.38%) | 1186.4 (617.49, 1916.01) | -49.93% (-64.61%, -28.07%) | 80 (34.29, 128.94) | -1.63% (-19.07%, 10.42%) | 1266.4 (656.09, 2036.95) | -48.33% (-63.09%, -26.81%) |
| Fujian | Age-standardized rate,  per 100,000 people | 47.28 (19.12, 76.45) | -47.17% (-67.15%, -27.91%) | 807.68 (365.43, 1240.5) | -53.86% (-70.18%, -35.65%) | 76.06 (31.82, 122.64) | -16.02% (-33.49%, -6.87%) | 883.74 (402.72, 1344.8) | -52% (-67.99%, -34.55%) |
| Gansu | Age-standardized rate,  per 100,000 people | 76.89 (26.4, 122.87) | -32.86% (-56.71%, -9.09%) | 1388.44 (496.5, 2122.11) | -38.34% (-58.4%, -15.34%) | 70.23 (23.6, 119) | 2.24% (-19.77%, 18.38%) | 1458.67 (527.31, 2238.67) | -37.14% (-56.87%, -14.87%) |
| Guangdong | Age-standardized rate,  per 100,000 people | 56.69 (16.15, 94.07) | -43.25% (-69.63%, -22%) | 1001.83 (328.01, 1618.61) | -48.21% (-69.74%, -27.93%) | 64.37 (23.92, 105.71) | -11.43% (-33.18%, -1%) | 1066.19 (352.93, 1715.9) | -46.88% (-68.33%, -27.16%) |
| Guangxi | Age-standardized rate,  per 100,000 people | 62.38 (21.43, 101.6) | -32.19% (-59.92%, -7.55%) | 1209.2 (438.55, 1925.87) | -36.97% (-61.34%, -11.83%) | 70.92 (25, 117.69) | -11.27% (-34.65%, 2.86%) | 1280.12 (472.27, 2036.13) | -35.95% (-60.42%, -11.67%) |
| Guizhou | Age-standardized rate,  per 100,000 people | 75.07 (35.5, 121.34) | -43.73% (-61.98%, -25.38%) | 1435.98 (758.69, 2301.37) | -48.3% (-65.65%, -29.28%) | 91.28 (37.34, 146.04) | -0.04% (-21.21%, 14.59%) | 1527.26 (800.33, 2434.57) | -46.76% (-63.8%, -28.09%) |
| Hainan | Age-standardized rate,  per 100,000 people | 64.65 (20.52, 110.29) | -39.13% (-65.37%, -12.59%) | 1190.17 (408.25, 1951.01) | -41.63% (-64.3%, -14.02%) | 73.84 (24.72, 120.9) | -9.89% (-32.12%, 1.39%) | 1264.01 (433.6, 2056.06) | -40.41% (-62.56%, -13.55%) |
| Hebei | Age-standardized rate,  per 100,000 people | 116.57 (53.39, 182.47) | -32.56% (-54.38%, -10.28%) | 2163.16 (994.54, 3356.18) | -34.13% (-54.61%, -11.66%) | 116.78 (51.29, 186.07) | -1.98% (-21.96%, 9.23%) | 2279.94 (1059.46, 3517.34) | -33% (-53.37%, -10.99%) |
| Heilongjiang | Age-standardized rate,  per 100,000 people | 120.09 (25.32, 196.04) | -43.66% (-66.42%, -25.63%) | 2315.13 (609.34, 3591.72) | -47.58% (-64.48%, -30.7%) | 103.23 (33.86, 173.05) | -11.68% (-34.54%, -0.5%) | 2418.36 (663.52, 3746.67) | -46.65% (-63.51%, -30.07%) |
| Henan | Age-standardized rate,  per 100,000 people | 101.06 (36.9, 159.82) | -30.32% (-55.67%, -9.4%) | 1878.38 (740.08, 2899.53) | -34.14% (-58.14%, -12.86%) | 105.84 (44.1, 171.14) | -1.29% (-21.96%, 11.06%) | 1984.22 (790.41, 3023.66) | -32.95% (-56.24%, -12.2%) |
| Hong Kong | Age-standardized rate,  per 100,000 people | 21 (9.81, 32.17) | -61.47% (-70.83%, -49.64%) | 403.09 (203.17, 602.99) | -61.53% (-70.85%, -49.23%) | 72.7 (33.02, 114.25) | -3.18% (-18.46%, 8.73%) | 475.8 (246.27, 700.12) | -57.63% (-66.45%, -45.89%) |
| Hubei | Age-standardized rate,  per 100,000 people | 85.35 (38.01, 132.22) | -54.13% (-69.65%, -39.81%) | 1454.28 (689.66, 2215.93) | -58.59% (-71.94%, -45.67%) | 84.01 (35.33, 134.46) | -12.66% (-32.28%, -1.45%) | 1538.3 (721.53, 2342.35) | -57.36% (-71.13%, -44.83%) |
| Hunan | Age-standardized rate,  per 100,000 people | 87.48 (39.7, 134.02) | -44.94% (-64.14%, -27.07%) | 1524.63 (731.91, 2339.8) | -49.8% (-66.45%, -34.04%) | 83.35 (35.87, 130.68) | -1.07% (-19.87%, 12.22%) | 1607.98 (775.1, 2454.69) | -48.48% (-64.89%, -33.07%) |
| Inner Mongolia | Age-standardized rate,  per 100,000 people | 118.38 (40.82, 188.52) | -42.27% (-61.84%, -25.26%) | 2173.74 (820.9, 3344.6) | -45.9% (-62.98%, -27.97%) | 108.94 (46.26, 172.44) | -6.14% (-24.31%, 4.84%) | 2282.68 (873.78, 3504.65) | -44.78% (-61.86%, -27.25%) |
| Jiangsu | Age-standardized rate,  per 100,000 people | 49.16 (19.86, 79.57) | -41.91% (-62.34%, -22.52%) | 796.82 (365.3, 1238.69) | -48.18% (-64.25%, -31.43%) | 87.34 (36.7, 142.28) | -2.16% (-20.41%, 10.25%) | 884.15 (403.76, 1368.09) | -45.66% (-62.07%, -29.4%) |
| Jiangxi | Age-standardized rate,  per 100,000 people | 81.49 (37.93, 123.52) | -56.29% (-70.31%, -41.73%) | 1413.11 (714.71, 2110.62) | -61.75% (-73.55%, -48.09%) | 84.28 (40.39, 132.18) | -14.61% (-28.78%, -5.29%) | 1497.39 (758.69, 2225.13) | -60.52% (-72.31%, -47.1%) |
| Jilin | Age-standardized rate,  per 100,000 people | 119.72 (34.3, 194.33) | -49.18% (-73.1%, -31.65%) | 2163.93 (718.52, 3427.92) | -53.71% (-73.42%, -37.31%) | 120.24 (46.78, 193.11) | -14.32% (-33.39%, -4.46%) | 2284.17 (777.43, 3602.42) | -52.56% (-71.87%, -36.45%) |
| Liaoning | Age-standardized rate,  per 100,000 people | 103.65 (22.99, 174.4) | -19.98% (-48.26%, 6.72%) | 1917.76 (524.43, 3105.51) | -22.7% (-46.97%, 4.55%) | 104.55 (36.69, 176.63) | -0.34% (-20.76%, 11.74%) | 2022.3 (563.78, 3253.46) | -21.79% (-45.45%, 4.62%) |
| Macao | Age-standardized rate,  per 100,000 people | 33.88 (13.75, 53.09) | -53.27% (-65.75%, -38.59%) | 636.24 (284.93, 973.69) | -58.15% (-69.09%, -44.32%) | 91.71 (39.9, 145.4) | -1.76% (-20.76%, 11.75%) | 727.95 (332.08, 1098.98) | -54.89% (-65.81%, -41.56%) |
| Ningxia | Age-standardized rate,  per 100,000 people | 87.39 (20.22, 148.36) | -35.24% (-64.42%, -11.59%) | 1601.99 (429.35, 2623.56) | -40.5% (-65.81%, -16.36%) | 84.07 (27.88, 139.15) | -0.29% (-23.84%, 13.22%) | 1686.06 (467.79, 2739.66) | -39.28% (-64.13%, -15.44%) |
| Qinghai | Age-standardized rate,  per 100,000 people | 111.35 (52.42, 171.04) | -22.26% (-46.35%, 3.18%) | 2050.08 (1086.84, 3162.53) | -30.78% (-52.02%, -5.54%) | 84.2 (39.88, 131.03) | -0.84% (-17.73%, 11.96%) | 2134.28 (1142.79, 3274.23) | -29.94% (-50.77%, -5.29%) |
| Shaanxi | Age-standardized rate,  per 100,000 people | 100.8 (38.4, 159.61) | -38.95% (-62.08%, -19.07%) | 1793.9 (763.56, 2758.29) | -44.86% (-63.42%, -24.39%) | 101.91 (44.53, 160.03) | 0.08% (-18.51%, 12.41%) | 1895.81 (812.53, 2905.38) | -43.5% (-61.78%, -23.58%) |
| Shandong | Age-standardized rate,  per 100,000 people | 78.99 (26.42, 124.87) | -33.71% (-59.61%, -11.73%) | 1392.52 (512.23, 2148.69) | -38.08% (-59.8%, -14.94%) | 100.84 (41.28, 163.37) | -3.68% (-21.28%, 6.83%) | 1493.36 (561.35, 2291.26) | -36.55% (-57.7%, -14.6%) |
| Shanghai | Age-standardized rate,  per 100,000 people | 35.84 (11.14, 63.16) | -47.43% (-70.98%, -28.11%) | 572.46 (219.1, 967.47) | -52.83% (-72.73%, -34.2%) | 70.22 (25.74, 115.55) | -11.55% (-31.2%, -0.57%) | 642.68 (255.79, 1078.78) | -50.29% (-70%, -32.3%) |
| Shanxi | Age-standardized rate,  per 100,000 people | 91.1 (27.27, 142.26) | -35.13% (-59.97%, -15.42%) | 1691.3 (556.53, 2595.56) | -39.78% (-60.9%, -18.24%) | 101.52 (38.38, 163.06) | 3.94% (-13.74%, 17.53%) | 1792.81 (594.64, 2725.02) | -38.31% (-58.86%, -17.29%) |
| Sichuan | Age-standardized rate,  per 100,000 people | 58.36 (25.16, 94.74) | -34.83% (-55.88%, -12.99%) | 1078.38 (516.94, 1703.19) | -39.79% (-57.68%, -19.38%) | 76.29 (31.39, 121.26) | -0.51% (-18.89%, 12.58%) | 1154.67 (558.64, 1811) | -38.18% (-55.37%, -18.09%) |
| Tianjin | Age-standardized rate,  per 100,000 people | 88.69 (14.21, 155.43) | -32.34% (-66.02%, -6.83%) | 1560.24 (340.28, 2595.6) | -36.71% (-63.07%, -12.69%) | 100.15 (29.5, 170.42) | -2.74% (-31.22%, 10.49%) | 1660.39 (371.87, 2754.33) | -35.35% (-61.27%, -12.21%) |
| Xinjiang | Age-standardized rate,  per 100,000 people | 97.61 (48.04, 148.8) | -47.15% (-60.93%, -28.93%) | 1963.74 (997.53, 2961.42) | -49.4% (-63.12%, -30.34%) | 94.19 (43.84, 144.5) | 2.98% (-11.75%, 13.9%) | 2057.93 (1049.82, 3084.42) | -48.19% (-61.8%, -29.52%) |
| Xizang | Age-standardized rate,  per 100,000 people | 107.27 (61.65, 166.42) | -57.44% (-70.07%, -42.65%) | 2307.11 (1272.05, 3626.99) | -60.22% (-73.48%, -44.96%) | 91.67 (49.34, 140.53) | -8.9% (-24.33%, 3.27%) | 2398.77 (1336.41, 3730.41) | -59.35% (-72.57%, -44.23%) |
| Yunnan | Age-standardized rate,  per 100,000 people | 81.68 (37.5, 127.63) | -26.81% (-49.15%, -3.2%) | 1575.59 (780.09, 2418.59) | -30.5% (-51.33%, -6.27%) | 85.16 (36.84, 136.03) | 3.36% (-13.35%, 16.51%) | 1660.75 (829.09, 2536.98) | -29.32% (-49.69%, -5.71%) |
| Zhejiang | Age-standardized rate,  per 100,000 people | 56.2 (25.68, 87.54) | -40.64% (-59.92%, -21.41%) | 888.03 (440.26, 1345.44) | -47.45% (-63.66%, -29.29%) | 83.68 (39.16, 128.06) | -6.17% (-20.31%, 2.66%) | 971.71 (483.9, 1459) | -45.38% (-61.57%, -27.98%) |
| **Diabetes and kidney diseases** | | | | | | | | | |
| Anhui | Absolute number,  thousands | 2.93 (1.16, 4.79) | 93.2% (34.67%, 161.59%) | 58.66 (22.13, 94.9) | 51.63% (3.09%, 107.41%) | 98.22 (17.91, 180.23) | 247.8% (158.48%, 292.34%) | 156.88 (41.86, 262.84) | 134.4% (48.11%, 182.73%) |
| Beijing | Absolute number,  thousands | 1.03 (0.37, 1.74) | 192.61% (104.75%, 310.83%) | 20.58 (7.38, 34.74) | 142.85% (68.3%, 243.15%) | 40.46 (6.94, 74.27) | 553.2% (433.9%, 659.19%) | 61.03 (15.3, 106.71) | 316.13% (170.82%, 414.1%) |
| Chongqing | Absolute number,  thousands | 2.16 (0.85, 3.61) | 341.62% (197.24%, 539.23%) | 42.76 (16.69, 72.26) | 239.72% (125.39%, 392.81%) | 48.77 (9.19, 90.59) | 570.59% (395.03%, 652.96%) | 91.53 (26.08, 153.11) | 360.89% (226.4%, 488.71%) |
| Fujian | Absolute number,  thousands | 1.61 (0.59, 2.61) | 133.98% (56.25%, 224.5%) | 33.28 (12.36, 53.96) | 92.62% (29.23%, 173.59%) | 58.53 (10.76, 108.1) | 353.45% (251.43%, 414.15%) | 91.81 (21.97, 158.89) | 204.16% (96.69%, 272.43%) |
| Gansu | Absolute number,  thousands | 1.26 (0.51, 2.05) | 156.5% (73.52%, 243.2%) | 26.18 (10.29, 42.88) | 100.8% (32.29%, 171.89%) | 26.01 (5.09, 47.79) | 222.87% (146.04%, 266.45%) | 52.19 (14.19, 87.53) | 147.42% (65.72%, 197.94%) |
| Guangdong | Absolute number,  thousands | 4.04 (1.62, 6.58) | 129.77% (59.75%, 212.61%) | 85.53 (32.84, 142.24) | 109.5% (42.84%, 190.21%) | 177.76 (30.11, 331.03) | 441.16% (336.95%, 526.85%) | 263.28 (63.59, 458.11) | 257.37% (128.89%, 332.6%) |
| Guangxi | Absolute number,  thousands | 2.63 (1.1, 4.26) | 134.36% (62.75%, 223.69%) | 55.57 (23.34, 89.81) | 99.57% (38.17%, 180.9%) | 72.99 (14.58, 131.82) | 262.86% (152.23%, 316.19%) | 128.56 (37.28, 214.16) | 168.05% (75.56%, 227.49%) |
| Guizhou | Absolute number,  thousands | 2.3 (1.1, 3.68) | 102.81% (35.67%, 178.31%) | 49.01 (23.24, 78.63) | 70.14% (10.88%, 143.52%) | 46.52 (10.6, 85.15) | 232.64% (149.43%, 278.9%) | 95.53 (34.32, 158.14) | 123.24% (49.94%, 179.73%) |
| Hainan | Absolute number,  thousands | 0.53 (0.19, 0.9) | 189.49% (93.35%, 323.49%) | 10.92 (3.77, 18.69) | 149.12% (63.64%, 269.2%) | 17.41 (2.84, 32.49) | 416.83% (298.57%, 493.9%) | 28.32 (6.63, 48.69) | 265.44% (136.1%, 367.55%) |
| Hebei | Absolute number,  thousands | 4.47 (1.85, 7.35) | 189.21% (99.82%, 298.83%) | 94.25 (38.88, 155.68) | 152.32% (74.8%, 253.63%) | 107.44 (20.74, 197.59) | 307.78% (182.19%, 366.08%) | 201.7 (60.42, 337.44) | 216.62% (121.25%, 292.93%) |
| Heilongjiang | Absolute number,  thousands | 1.87 (0.72, 3) | 88.99% (25.61%, 160.37%) | 42.04 (15.64, 67.69) | 55.61% (0.6%, 117.81%) | 58.41 (11.37, 106.05) | 249.78% (185.43%, 291.9%) | 100.45 (26.64, 167.98) | 129.78% (50.42%, 181.68%) |
| Henan | Absolute number,  thousands | 4.93 (2.09, 8.01) | 165.91% (79.59%, 250.82%) | 102.78 (41.96, 169.59) | 127.85% (50.23%, 207.65%) | 133.98 (26.1, 247.07) | 278.17% (162.22%, 325.47%) | 236.76 (64.61, 404.84) | 193.98% (102.48%, 249.85%) |
| Hong Kong | Absolute number,  thousands | 0.55 (0.24, 0.92) | 136.87% (71.63%, 220.56%) | 8.82 (3.94, 14.52) | 80.7% (30.61%, 145.02%) | 13.05 (3.16, 22.73) | 316.51% (271.21%, 401.88%) | 21.87 (7.47, 34.76) | 172.88% (119.36%, 223.33%) |
| Hubei | Absolute number,  thousands | 3.57 (1.62, 5.74) | 116.97% (54.91%, 199.93%) | 72.48 (32.29, 116.62) | 77.84% (23.9%, 148.15%) | 81.8 (17.08, 146.49) | 230.79% (140.44%, 275.58%) | 154.28 (53.64, 251.16) | 135.6% (63.93%, 187.18%) |
| Hunan | Absolute number,  thousands | 5.45 (2.42, 8.94) | 113.68% (49.13%, 193.29%) | 109.17 (48.73, 177.09) | 66.59% (14.85%, 131.05%) | 101.76 (21.32, 184.63) | 213.05% (151.69%, 254.07%) | 210.93 (75.05, 354.35) | 115.15% (56.52%, 164.21%) |
| Inner Mongolia | Absolute number,  thousands | 1.17 (0.49, 1.87) | 178.12% (77.85%, 293.82%) | 25.13 (10.72, 40.33) | 125.24% (42.19%, 219.04%) | 36.26 (6.95, 66.2) | 339.69% (229.72%, 398.64%) | 61.39 (17.89, 104.02) | 216.37% (111.72%, 289.08%) |
| Jiangsu | Absolute number,  thousands | 4.5 (1.53, 7.62) | 158.66% (69.89%, 250.02%) | 84.6 (28.71, 144.65) | 107.76% (31.42%, 183.97%) | 145.47 (30.01, 259.53) | 286.01% (202.66%, 341.82%) | 230.07 (59.22, 397.06) | 193.43% (97.91%, 255.78%) |
| Jiangxi | Absolute number,  thousands | 2.52 (1.11, 4.1) | 62.56% (14.72%, 121.97%) | 50.98 (22.1, 84.02) | 30.2% (-9.05%, 82.06%) | 56.43 (10.52, 104.51) | 249.04% (163.58%, 294.61%) | 107.42 (33.53, 176.05) | 94.15% (28.77%, 143.73%) |
| Jilin | Absolute number,  thousands | 1.47 (0.6, 2.43) | 115.09% (43.81%, 193.62%) | 32.45 (12.82, 54.51) | 80.94% (18.04%, 151.41%) | 42.23 (7.63, 78.51) | 248.06% (166.22%, 291.81%) | 74.67 (20.54, 125.7) | 148.38% (54.96%, 200.54%) |
| Liaoning | Absolute number,  thousands | 3.88 (1.31, 6.58) | 232.64% (136.46%, 358.15%) | 81.78 (27.26, 140.13) | 189.67% (97.98%, 304.4%) | 83.28 (16.27, 154.36) | 257.75% (197.68%, 308.01%) | 165.06 (43.79, 285.57) | 220.43% (141.07%, 283.34%) |
| Macao | Absolute number,  thousands | 0.03 (0.01, 0.05) | 112.11% (56.29%, 188.5%) | 0.58 (0.27, 0.91) | 96.09% (43.71%, 171.05%) | 0.89 (0.15, 1.66) | 573.73% (412.1%, 654.68%) | 1.48 (0.45, 2.49) | 242.83% (135.97%, 326.82%) |
| Ningxia | Absolute number,  thousands | 0.27 (0.1, 0.46) | 238.15% (118.7%, 372.25%) | 6.04 (2.07, 10.17) | 182.71% (78.49%, 297.89%) | 7.01 (1.21, 13.02) | 401.11% (275.71%, 470.97%) | 13.05 (3.38, 21.94) | 269.09% (148.08%, 356.23%) |
| Qinghai | Absolute number,  thousands | 0.37 (0.16, 0.61) | 233.67% (121.92%, 361.98%) | 8.39 (3.51, 13.88) | 168.2% (77.02%, 274.64%) | 6.42 (1.16, 11.97) | 361.4% (227.84%, 434.1%) | 14.81 (5.17, 24.83) | 227.72% (120.27%, 312.26%) |
| Shaanxi | Absolute number,  thousands | 2.24 (0.97, 3.62) | 150.68% (69.51%, 249.41%) | 46.74 (19.87, 76.3) | 102.54% (35.44%, 185.22%) | 44.52 (8.98, 83.24) | 238% (160.18%, 284.15%) | 91.25 (28.99, 147.87) | 151.77% (78.6%, 210.21%) |
| Shandong | Absolute number,  thousands | 4.12 (1.67, 6.75) | 133.79% (60.96%, 225.53%) | 84.71 (34.73, 140.46) | 103.21% (35.43%, 188.77%) | 155.96 (29.3, 279.79) | 303.52% (219.66%, 357.69%) | 240.67 (65.53, 412.38) | 199.58% (109.43%, 260.18%) |
| Shanghai | Absolute number,  thousands | 1.54 (0.49, 2.71) | 167.13% (67.88%, 276.16%) | 28.1 (9.03, 49.33) | 116.27% (36.35%, 208.04%) | 46.62 (8.84, 88.04) | 343.12% (263.98%, 408%) | 74.71 (16.68, 130.73) | 217.78% (125.28%, 286.34%) |
| Shanxi | Absolute number,  thousands | 1.62 (0.64, 2.63) | 148.74% (62.74%, 247.31%) | 34.04 (13.36, 55.82) | 105.06% (32.82%, 186.73%) | 58.99 (11.2, 110.56) | 317.02% (220.35%, 377.63%) | 93.02 (24.45, 160.57) | 202.57% (86.49%, 266.25%) |
| Sichuan | Absolute number,  thousands | 5.25 (2.06, 8.95) | 106.61% (39.25%, 187.35%) | 107 (42.3, 180.81) | 64.21% (12.62%, 135.82%) | 128.19 (24.19, 239.71) | 130.47% (82.68%, 159.75%) | 235.18 (70.34, 401.8) | 94.72% (40.35%, 137.08%) |
| Tianjin | Absolute number,  thousands | 0.89 (0.29, 1.59) | 233.2% (118.25%, 365.55%) | 18.38 (5.93, 32.17) | 190.4% (90.14%, 309.64%) | 26.26 (4.83, 48.01) | 424.47% (312.25%, 491.87%) | 44.64 (10.87, 77.74) | 293.77% (177.38%, 384.54%) |
| Xinjiang | Absolute number,  thousands | 1.26 (0.49, 2.06) | 170.9% (85.24%, 279.81%) | 31 (12.29, 51.73) | 151.77% (67.95%, 257.01%) | 36.69 (6.72, 67.5) | 441.46% (325.47%, 511.14%) | 67.69 (18.74, 114.45) | 254.62% (145.86%, 341.3%) |
| Xizang | Absolute number,  thousands | 0.13 (0.06, 0.2) | 23.24% (-15.19%, 72.41%) | 3.32 (1.65, 5.35) | 16.88% (-20.81%, 67.02%) | 2.69 (0.56, 4.92) | 286.02% (136.3%, 357.9%) | 6.01 (2.42, 9.61) | 69.93% (6.76%, 134.1%) |
| Yunnan | Absolute number,  thousands | 2.85 (1.21, 4.6) | 159.32% (73.01%, 264.08%) | 62.32 (26.05, 101.64) | 120.23% (46.31%, 210.19%) | 54.95 (10.05, 101.31) | 285.08% (193.12%, 336.76%) | 117.27 (37.37, 196.07) | 175.49% (91.52%, 244.64%) |
| Zhejiang | Absolute number,  thousands | 3.34 (1.32, 5.22) | 163.08% (81.27%, 260.5%) | 63.78 (26.2, 99.06) | 119.85% (48.96%, 206.2%) | 93.4 (18.22, 167.64) | 375.59% (233.01%, 440.25%) | 157.19 (42.44, 258.4) | 223.09% (117.03%, 292.76%) |
| Anhui | Age-standardized  percent | 20.19% (8.43%, 30.98%) | -0.34% (-20.88%, 12.6%) | 18.85% (7.81%, 29.27%) | 8.61% (-13.53%, 22.46%) | 21.61% (4.04%, 36.09%) | 21.34% (-16.84%, 35.32%) | 20.55% (5.8%, 33.48%) | 17.15% (-25.55%, 34.82%) |
| Beijing | Age-standardized  percent | 19.85% (7.85%, 31.01%) | 1.38% (-23.79%, 16.58%) | 19.27% (7.63%, 30.2%) | 9.4% (-16.8%, 24.35%) | 23.09% (4.14%, 38.77%) | 34.49% (4.87%, 51.42%) | 21.63% (5.8%, 35.39%) | 23.99% (-22.77%, 43.08%) |
| Chongqing | Age-standardized  percent | 18.96% (7.91%, 29.25%) | 2.11% (-20.31%, 16.24%) | 17.88% (7.45%, 27.48%) | 14.39% (-10.73%, 31.01%) | 21.27% (4.06%, 35.93%) | 25.84% (-9.57%, 41.77%) | 19.63% (6.21%, 31.92%) | 22.25% (-14.77%, 41.05%) |
| Fujian | Age-standardized  percent | 19.31% (7.14%, 30.34%) | 2.97% (-23.56%, 17.55%) | 18.13% (6.68%, 28.11%) | 13.69% (-14.34%, 29.5%) | 21.63% (4.13%, 36.4%) | 27.79% (-5.36%, 42.05%) | 20.18% (5.68%, 33.37%) | 23.55% (-21.04%, 40.55%) |
| Gansu | Age-standardized  percent | 18.02% (7.54%, 27.79%) | 1.79% (-18.47%, 16.72%) | 16.58% (6.98%, 25.57%) | 14.4% (-10.64%, 31.13%) | 20.33% (4.23%, 34.15%) | 21.02% (-11.15%, 34.88%) | 18.26% (5.71%, 29.25%) | 20.18% (-15.02%, 37.84%) |
| Guangdong | Age-standardized  percent | 18.43% (7.42%, 28.73%) | 4.35% (-19.56%, 20.39%) | 17.54% (6.95%, 27.27%) | 16.88% (-8.91%, 33.96%) | 21.8% (3.87%, 36.58%) | 31.47% (2.31%, 47.4%) | 20.05% (5.58%, 33.33%) | 28.31% (-15.14%, 47.88%) |
| Guangxi | Age-standardized  percent | 18.59% (8.31%, 28.07%) | -1.78% (-23.8%, 12.88%) | 16.92% (7.44%, 25.69%) | 9.44% (-16.38%, 26.31%) | 19.81% (4.41%, 32.71%) | 21.07% (-17.92%, 36.65%) | 18.48% (6.04%, 29.43%) | 16.84% (-22.91%, 34.04%) |
| Guizhou | Age-standardized  percent | 19.67% (9.58%, 29.54%) | -1.4% (-20.61%, 13.07%) | 17.81% (8.61%, 26.6%) | 11.17% (-10.33%, 28.42%) | 19.93% (5.07%, 33.04%) | 17.66% (-18.16%, 33.25%) | 18.78% (7.28%, 29.54%) | 15.32% (-18.26%, 33.79%) |
| Hainan | Age-standardized  percent | 18.22% (6.77%, 28.59%) | 3.27% (-20.4%, 18.03%) | 17.2% (6.3%, 27.06%) | 14.85% (-10.72%, 30.81%) | 21.5% (3.49%, 36.12%) | 26.74% (-4.09%, 41.62%) | 19.57% (4.95%, 32.34%) | 24.39% (-14.08%, 40.25%) |
| Hebei | Age-standardized  percent | 19.11% (8.83%, 29%) | -2.32% (-20.96%, 11.05%) | 17.65% (7.88%, 26.84%) | 7.75% (-13.94%, 23.86%) | 21.06% (4.31%, 35.57%) | 23.97% (-18.2%, 38.84%) | 19.34% (6.48%, 30.94%) | 16.5% (-19.49%, 33.64%) |
| Heilongjiang | Age-standardized  percent | 18.69% (7.51%, 28.72%) | 1.17% (-20.68%, 15.98%) | 17.47% (6.86%, 27.2%) | 7.93% (-17.27%, 23.34%) | 21.02% (4.6%, 35.17%) | 22.44% (-9.5%, 36.25%) | 19.46% (5.89%, 32.01%) | 17.83% (-22.48%, 35.84%) |
| Henan | Age-standardized  percent | 19.67% (7.99%, 30.5%) | 1.82% (-21.01%, 15.9%) | 18.53% (7.43%, 29.02%) | 14.02% (-12.1%, 30.28%) | 21.35% (4.26%, 35.84%) | 22.65% (-21.29%, 35.13%) | 20.09% (6.25%, 32.78%) | 20.2% (-21.91%, 36.26%) |
| Hong Kong | Age-standardized  percent | 24.38% (11.88%, 36.25%) | 11.31% (-1.74%, 23.22%) | 23.38% (11.56%, 34.53%) | 16.89% (3.62%, 29.05%) | 29.92% (7.09%, 46.82%) | 51.98% (37.35%, 80.27%) | 27.23% (9.26%, 41.29%) | 36.9% (8.93%, 57.46%) |
| Hubei | Age-standardized  percent | 19.95% (9.39%, 30.61%) | -3.13% (-23.37%, 12.26%) | 18.75% (8.63%, 28.65%) | 6.71% (-16.46%, 23.61%) | 20.36% (5.22%, 33.29%) | 23.05% (-15.45%, 38.79%) | 19.6% (7.11%, 30.96%) | 13.86% (-21.56%, 30.82%) |
| Hunan | Age-standardized  percent | 19.68% (9.42%, 29.83%) | 4.96% (-16.49%, 20.03%) | 18.04% (8.54%, 27.16%) | 17.03% (-5.01%, 34.63%) | 20.87% (4.68%, 34.84%) | 23.32% (-6.7%, 35.68%) | 19.37% (6.77%, 30.73%) | 22.23% (-12.62%, 40.31%) |
| Inner Mongolia | Age-standardized  percent | 19.46% (8.64%, 29.64%) | -2.72% (-25.23%, 13.45%) | 18.16% (8.14%, 27.44%) | 9.48% (-16.02%, 27.6%) | 21.54% (4.55%, 36.16%) | 24.44% (-17.3%, 39.66%) | 20.01% (6.5%, 32.27%) | 18.67% (-22.22%, 38.87%) |
| Jiangsu | Age-standardized  percent | 20.49% (7.2%, 32.69%) | 1% (-26.09%, 16.16%) | 19.86% (7.1%, 31.61%) | 12.31% (-17.91%, 27.76%) | 21.87% (4.57%, 36.59%) | 27.49% (-11.9%, 43.93%) | 21.14% (5.79%, 34.56%) | 21.15% (-21.31%, 38.46%) |
| Jiangxi | Age-standardized  percent | 19.6% (9.05%, 29.68%) | -0.64% (-21.79%, 14.41%) | 18.1% (8.3%, 27.16%) | 11.25% (-11.45%, 28.23%) | 21.12% (4.02%, 35.46%) | 22.45% (-13.9%, 38.62%) | 19.58% (6.91%, 31.75%) | 18.52% (-21.36%, 39.76%) |
| Jilin | Age-standardized  percent | 18.69% (7.48%, 28.8%) | -1.77% (-26.11%, 14.55%) | 17.48% (6.94%, 26.97%) | 7.91% (-19.29%, 25.9%) | 21.27% (3.92%, 36.01%) | 23.22% (-14.61%, 37.59%) | 19.46% (5.82%, 31.77%) | 17.48% (-27.3%, 36.33%) |
| Liaoning | Age-standardized  percent | 19.56% (6.9%, 31.35%) | 5.4% (-16.34%, 18.43%) | 18.9% (6.7%, 30.41%) | 13.3% (-8.64%, 27.63%) | 21.45% (4.4%, 36.18%) | 24.01% (-2.22%, 38.48%) | 20.15% (5.92%, 33.31%) | 18.99% (-8.69%, 33.26%) |
| Macao | Age-standardized  percent | 23.94% (11.93%, 35.23%) | 4.28% (-5.22%, 15.79%) | 22.42% (10.77%, 32.85%) | 8.51% (-1.37%, 19.72%) | 23.17% (4.03%, 38.85%) | 29.07% (-6.69%, 44.07%) | 22.86% (7.57%, 35.97%) | 15.45% (-18.42%, 31.81%) |
| Ningxia | Age-standardized  percent | 18.81% (7.25%, 29.16%) | 2.26% (-22.67%, 17.4%) | 17.52% (6.61%, 27.08%) | 17.19% (-11.69%, 35.18%) | 20.8% (3.84%, 34.77%) | 22.47% (-15.05%, 36.94%) | 19.07% (5.72%, 31.14%) | 22.24% (-21.03%, 40.32%) |
| Qinghai | Age-standardized  percent | 19.19% (8.64%, 29.01%) | 1.58% (-19.62%, 17.23%) | 17.62% (7.77%, 26.93%) | 16.03% (-9.01%, 34.3%) | 20.77% (4.18%, 34.69%) | 23.02% (-21.1%, 39.1%) | 18.8% (6.95%, 29.37%) | 20.28% (-14.45%, 38.5%) |
| Shaanxi | Age-standardized  percent | 19.87% (9.01%, 30.21%) | 4.09% (-17.08%, 19.1%) | 18.32% (8.33%, 28.19%) | 17.74% (-5.54%, 34.43%) | 21.03% (4.74%, 34.84%) | 25.42% (-9.34%, 39.77%) | 19.56% (7.06%, 30.77%) | 22.72% (-9.97%, 39.81%) |
| Shandong | Age-standardized  percent | 20.18% (8.2%, 31.23%) | 1.68% (-19.72%, 15.16%) | 18.75% (7.78%, 29.15%) | 12.55% (-9.54%, 26.73%) | 21.77% (4.3%, 36.01%) | 26.14% (-6.4%, 41.13%) | 20.67% (6.13%, 33.57%) | 22.07% (-16.96%, 37.9%) |
| Shanghai | Age-standardized  percent | 18.69% (6.43%, 29.71%) | 3.21% (-21.33%, 18.59%) | 18.49% (6.32%, 29.1%) | 11.45% (-13.55%, 25.49%) | 22.12% (4.17%, 37.78%) | 32.78% (6.88%, 49.27%) | 20.65% (5.48%, 34.27%) | 24.19% (-10.57%, 40.8%) |
| Shanxi | Age-standardized  percent | 18.93% (8.04%, 28.72%) | 2.94% (-18.3%, 17.19%) | 17.73% (7.44%, 27.02%) | 13.8% (-11.27%, 29.47%) | 21.5% (4.2%, 35.96%) | 24.48% (-9.09%, 38.57%) | 19.95% (5.99%, 32.5%) | 22.72% (-21.11%, 40.97%) |
| Sichuan | Age-standardized  percent | 19.3% (8.47%, 29.55%) | 4.33% (-16.97%, 17.74%) | 17.76% (7.62%, 27.24%) | 18.89% (-6.16%, 35.5%) | 21.53% (4.15%, 36.3%) | 22.32% (-9.49%, 35.25%) | 19.75% (6.29%, 32.24%) | 23.57% (-13.04%, 39.63%) |
| Tianjin | Age-standardized  percent | 19% (6.29%, 31.28%) | 5.13% (-23.47%, 20.65%) | 18.57% (6%, 30.31%) | 14.19% (-14.14%, 28.11%) | 22.39% (4.49%, 37.45%) | 34.34% (1.98%, 51.07%) | 20.69% (5.56%, 34.31%) | 25.92% (-11.33%, 42.08%) |
| Xinjiang | Age-standardized  percent | 18.66% (7.53%, 28.13%) | 3.63% (-15.67%, 17.46%) | 16.97% (6.78%, 25.78%) | 13.67% (-9.08%, 28.68%) | 21.31% (4.16%, 35.5%) | 25.01% (-9.66%, 40.07%) | 18.98% (5.91%, 30.34%) | 21.91% (-15.79%, 38.41%) |
| Xizang | Age-standardized  percent | 19.34% (9.96%, 29.54%) | 0.89% (-17.6%, 16.92%) | 16.16% (8.38%, 24.12%) | 9.86% (-10.84%, 29.18%) | 20.27% (5.09%, 33.69%) | 24.48% (-28.86%, 46.3%) | 17.63% (7.77%, 27.08%) | 17.6% (-19.04%, 43.6%) |
| Yunnan | Age-standardized  percent | 18.02% (8.38%, 27.01%) | 7.58% (-12.1%, 21.12%) | 16.34% (7.47%, 24.41%) | 23.5% (-2.08%, 40.54%) | 20.63% (4.19%, 34.76%) | 24.63% (-10.04%, 39.37%) | 18.07% (6.4%, 28.48%) | 28.11% (-7.46%, 45.63%) |
| Zhejiang | Age-standardized  percent | 20.96% (9.41%, 32.22%) | -1.31% (-22.19%, 12.92%) | 20.24% (9.19%, 30.62%) | 8.76% (-13.75%, 24.36%) | 22.53% (4.34%, 37.48%) | 30.14% (-9.82%, 46.71%) | 21.54% (6.65%, 34.6%) | 18.9% (-21.32%, 38%) |
| Anhui | Age-standardized rate,  per 100,000 people | 3.19 (1.28, 5.19) | -34.43% (-55%, -13.67%) | 62.29 (23.7, 102.14) | -37.45% (-56.93%, -15.76%) | 113.9 (20.93, 209.81) | 75.83% (17.37%, 101.16%) | 176.19 (46.14, 296.02) | 7.2% (-36%, 30.43%) |
| Beijing | Age-standardized rate,  per 100,000 people | 3.42 (1.23, 5.73) | -31.33% (-52.02%, -4.75%) | 63.91 (23.02, 107.46) | -33.72% (-53.97%, -6.68%) | 125.38 (21.8, 231.13) | 113.3% (66.49%, 150.5%) | 189.29 (48.11, 329.93) | 21.96% (-23.85%, 52.36%) |
| Chongqing | Age-standardized rate,  per 100,000 people | 4.08 (1.63, 6.79) | -16.1% (-42.61%, 19.35%) | 78.38 (30.78, 132.17) | -24.77% (-49.39%, 8.77%) | 99.57 (18.48, 181.58) | 80.92% (28.96%, 106.74%) | 177.95 (50.15, 299.46) | 11.76% (-24.22%, 43.75%) |
| Fujian | Age-standardized rate,  per 100,000 people | 3.23 (1.19, 5.25) | -23.67% (-49.52%, 5.32%) | 61.79 (22.81, 99.82) | -29.93% (-53.11%, -2.31%) | 104.94 (19.59, 193.2) | 78.64% (31.23%, 104.27%) | 166.73 (40.26, 286.94) | 13.47% (-27.63%, 39.3%) |
| Gansu | Age-standardized rate,  per 100,000 people | 4.05 (1.72, 6.45) | -18.79% (-44.14%, 8.91%) | 76.04 (31.17, 123.25) | -24.59% (-49.59%, 1.2%) | 75.67 (14.7, 139.42) | 49% (5.45%, 70.89%) | 151.71 (42.91, 252.48) | 0.06% (-33.49%, 21.75%) |
| Guangdong | Age-standardized rate,  per 100,000 people | 3.33 (1.37, 5.42) | -27.45% (-50.51%, -1.12%) | 63.87 (25.04, 105.35) | -30.29% (-52.44%, -3.59%) | 115.34 (19.71, 212.67) | 75.44% (36.54%, 102.98%) | 179.21 (44.49, 309.69) | 13.88% (-29.54%, 36.75%) |
| Guangxi | Age-standardized rate,  per 100,000 people | 4.02 (1.7, 6.46) | -10.27% (-36.82%, 22.55%) | 82.7 (35.23, 134.32) | -13.77% (-40.95%, 20.02%) | 113.9 (22.54, 206.82) | 75.59% (16.17%, 102.53%) | 196.6 (56.66, 328.81) | 22.29% (-22.69%, 50.76%) |
| Guizhou | Age-standardized rate,  per 100,000 people | 5.19 (2.49, 8.16) | -24.56% (-48.7%, 2.15%) | 102.51 (49.27, 163.11) | -28.11% (-51.84%, 0.7%) | 97.95 (22.59, 179.1) | 56.4% (7.45%, 80.1%) | 200.45 (72.69, 327.84) | -2.32% (-35.58%, 22.44%) |
| Hainan | Age-standardized rate,  per 100,000 people | 4.46 (1.6, 7.61) | -7.59% (-38.09%, 35.66%) | 87.9 (30.65, 150.14) | -11.47% (-41.61%, 29.69%) | 135.76 (22.45, 252.71) | 98.17% (47.87%, 130.13%) | 223.66 (53.11, 384.05) | 33.29% (-15.54%, 71.73%) |
| Hebei | Age-standardized rate,  per 100,000 people | 4.48 (1.86, 7.34) | 2.87% (-28.61%, 41.36%) | 86.29 (36.36, 142.74) | 0.33% (-29.58%, 38.25%) | 101.76 (19.45, 188.95) | 92.78% (25.26%, 123.78%) | 188.06 (56.86, 312.35) | 35.49% (-6.2%, 69.03%) |
| Heilongjiang | Age-standardized rate,  per 100,000 people | 3.59 (1.4, 5.74) | -40.48% (-59.46%, -20.31%) | 74.25 (28.9, 118.45) | -42.73% (-61.53%, -22.53%) | 113.45 (22.75, 204.42) | 70.63% (27.44%, 95.87%) | 187.7 (49.64, 313.95) | -4.3% (-38.73%, 18.45%) |
| Henan | Age-standardized rate,  per 100,000 people | 3.63 (1.52, 5.86) | -1.37% (-32.48%, 31.86%) | 72.54 (30.08, 118.75) | -3.22% (-36.75%, 30.06%) | 102.58 (20.2, 187.78) | 93.52% (27.22%, 119.23%) | 175.12 (48.01, 298.22) | 36.85% (-11.78%, 64.24%) |
| Hong Kong | Age-standardized rate,  per 100,000 people | 3.05 (1.32, 5.04) | -33.87% (-52.08%, -13.17%) | 54.08 (24.31, 88.59) | -38.93% (-56.15%, -17.81%) | 99.53 (23.32, 171.95) | 87.33% (64.77%, 128.2%) | 153.61 (49.04, 247.18) | 8.42% (-15.9%, 30.5%) |
| Hubei | Age-standardized rate,  per 100,000 people | 4.26 (2.01, 6.77) | -26.02% (-46.81%, -0.45%) | 79.57 (36.27, 126.85) | -30.01% (-50.28%, -3.56%) | 94.75 (19.86, 168.74) | 60.78% (7.03%, 84.89%) | 174.31 (60.1, 282.96) | 0.98% (-31.49%, 23.86%) |
| Hunan | Age-standardized rate,  per 100,000 people | 5.52 (2.53, 8.98) | -23.77% (-46.15%, 1.66%) | 105.43 (47.47, 170.13) | -30.12% (-51.76%, -4.28%) | 107.83 (22.74, 196.21) | 62.65% (22.35%, 84.24%) | 213.26 (76.15, 357.16) | -1.8% (-31.48%, 21.47%) |
| Inner Mongolia | Age-standardized rate,  per 100,000 people | 3.62 (1.56, 5.78) | -22.34% (-49.39%, 8.04%) | 68.73 (29.25, 108.93) | -25.45% (-51.88%, 4.98%) | 98.71 (19.47, 180.85) | 79.34% (21.83%, 104.82%) | 167.44 (50.76, 284.29) | 13.73% (-27.43%, 44.18%) |
| Jiangsu | Age-standardized rate,  per 100,000 people | 3.26 (1.12, 5.53) | -17.32% (-46.72%, 10.88%) | 59.52 (20.47, 101.75) | -21.95% (-49.67%, 5.38%) | 112.85 (23.74, 204.37) | 81.43% (28.49%, 108.63%) | 172.37 (43.79, 296.05) | 24.49% (-21.08%, 51.21%) |
| Jiangxi | Age-standardized rate,  per 100,000 people | 4.58 (2, 7.4) | -42.48% (-58.96%, -22.34%) | 85.51 (38.04, 140.2) | -48.11% (-63.5%, -28.17%) | 96.4 (18.45, 178.7) | 59.57% (11.62%, 82.15%) | 181.91 (56.81, 299.34) | -19.23% (-49.19%, 2.01%) |
| Jilin | Age-standardized rate,  per 100,000 people | 3.98 (1.7, 6.56) | -30.78% (-53.99%, -8.63%) | 78.87 (32.05, 129.49) | -32.3% (-54.24%, -8.31%) | 106.45 (19.11, 199.55) | 62.98% (12.92%, 85.21%) | 185.33 (50.16, 310.45) | 1.93% (-37.56%, 23.88%) |
| Liaoning | Age-standardized rate,  per 100,000 people | 5.21 (1.8, 8.81) | 7.81% (-23.72%, 47.11%) | 103.58 (34.93, 175.91) | 6.43% (-25.63%, 48.44%) | 112.63 (21.82, 207.47) | 64.75% (27.6%, 86.37%) | 216.21 (56.55, 371.98) | 30.49% (-4.51%, 56.57%) |
| Macao | Age-standardized rate,  per 100,000 people | 3.31 (1.5, 5.18) | -38.43% (-54.4%, -15.06%) | 60.27 (27.57, 93.98) | -43.91% (-58.9%, -23.25%) | 90.88 (15.42, 169.43) | 105.29% (48.62%, 134.88%) | 151.15 (46.53, 253.53) | -0.38% (-31.78%, 24.57%) |
| Ningxia | Age-standardized rate,  per 100,000 people | 3.87 (1.42, 6.41) | -17.68% (-46.73%, 16.01%) | 73.76 (25.81, 122.97) | -20.82% (-48.8%, 9.83%) | 77.82 (14.01, 143.93) | 57.4% (8.6%, 79.89%) | 151.58 (42.42, 255.08) | 6.3% (-30.06%, 31.21%) |
| Qinghai | Age-standardized rate,  per 100,000 people | 6.5 (2.84, 10.81) | 4.95% (-26.98%, 42.48%) | 128.42 (55.54, 213.48) | -3.07% (-33.75%, 32.89%) | 88.68 (16.52, 164.86) | 75.69% (11.64%, 103.04%) | 217.09 (78.9, 359.36) | 18.66% (-19.4%, 49.23%) |
| Shaanxi | Age-standardized rate,  per 100,000 people | 4.27 (1.88, 6.94) | -19.98% (-44.91%, 7.58%) | 81.42 (35.05, 133.16) | -24.57% (-48.65%, 4.3%) | 78.78 (16.17, 145.17) | 50.38% (6.4%, 71.6%) | 160.2 (53.06, 259.19) | -0.08% (-31.77%, 23.87%) |
| Shandong | Age-standardized rate,  per 100,000 people | 2.65 (1.08, 4.32) | -21.84% (-46.08%, 7.89%) | 52.13 (21.22, 85.89) | -21.67% (-46.87%, 8.99%) | 105.2 (20.16, 189.51) | 94.45% (44.04%, 121.19%) | 157.33 (41.77, 268.95) | 30.39% (-14.43%, 58.56%) |
| Shanghai | Age-standardized rate,  per 100,000 people | 3.79 (1.22, 6.65) | -22.46% (-50.83%, 8.21%) | 67.89 (21.98, 119.09) | -27.57% (-54.95%, 1.35%) | 118.7 (22.64, 224.48) | 73.95% (36.51%, 98.33%) | 186.6 (41.85, 328.01) | 15.2% (-21.36%, 40.51%) |
| Shanxi | Age-standardized rate,  per 100,000 people | 3.55 (1.48, 5.65) | -15.43% (-42.61%, 15.61%) | 67.67 (27.16, 110.26) | -20.23% (-47.64%, 10.58%) | 118.94 (22.61, 221.98) | 90.93% (37.25%, 120.03%) | 186.6 (49.4, 320.72) | 26.83% (-23.92%, 56.55%) |
| Sichuan | Age-standardized rate,  per 100,000 people | 3.77 (1.5, 6.4) | -1.93% (-33.89%, 32.52%) | 75.44 (30.01, 128.18) | -6.66% (-36.05%, 29.82%) | 102.79 (19.02, 192.26) | 68.18% (21.23%, 91.36%) | 178.22 (52.37, 301.96) | 25.57% (-15.17%, 54.78%) |
| Tianjin | Age-standardized rate,  per 100,000 people | 4.38 (1.43, 7.87) | -4.19% (-36.11%, 31.29%) | 84.74 (27.8, 148.22) | -4.75% (-36.68%, 33.54%) | 127.18 (23.79, 234.27) | 110.83% (54.6%, 141.84%) | 211.92 (51.66, 367.02) | 41.95% (-4.69%, 75.19%) |
| Xinjiang | Age-standardized rate,  per 100,000 people | 5.29 (2.03, 8.5) | -17.92% (-42.98%, 12.75%) | 112.42 (44.63, 184.91) | -19.2% (-45.11%, 13.53%) | 120.26 (22.64, 221.31) | 79.36% (29.54%, 102.93%) | 232.67 (66.52, 391.69) | 12.86% (-21.49%, 41.37%) |
| Xizang | Age-standardized rate,  per 100,000 people | 4.73 (2.37, 7.65) | -42.65% (-58.96%, -23.48%) | 104.19 (52.32, 166.7) | -45.81% (-62.84%, -24.67%) | 72.42 (16.26, 131.5) | 56.48% (-10.88%, 87.5%) | 176.61 (74.84, 281.46) | -25.96% (-51.38%, -0.63%) |
| Yunnan | Age-standardized rate,  per 100,000 people | 5.27 (2.23, 8.57) | -7.31% (-38.08%, 26.15%) | 105.05 (45.5, 169.28) | -11.34% (-40.39%, 23.16%) | 89.98 (16.7, 166.25) | 68.74% (17.55%, 91.86%) | 195.03 (63.46, 324.04) | 13.52% (-20.91%, 43.09%) |
| Zhejiang | Age-standardized rate,  per 100,000 people | 3.78 (1.52, 5.93) | -18.61% (-44.36%, 9.99%) | 67.08 (26.93, 104.15) | -23.96% (-48.43%, 4.87%) | 96.69 (19.14, 173.84) | 84.42% (24.09%, 110.56%) | 163.78 (45.48, 268.92) | 16.44% (-25.19%, 42.98%) |
| **Neoplasms** | | | | | | | | | |
| Anhui | Absolute number,  thousands | 8.42 (2.29, 18.99) | -14.8% (-44.85%, 33.02%) | 193.59 (50.98, 434.85) | -32.13% (-55.87%, 4.3%) | 7.84 (2.08, 14.85) | 115.54% (40.04%, 226.89%) | 201.43 (53.01, 447.13) | -30.27% (-54.49%, 7.44%) |
| Beijing | Absolute number,  thousands | 2.55 (0.88, 4.72) | 98.04% (35.17%, 210.49%) | 56.76 (18.92, 107.2) | 59.87% (9.86%, 142.01%) | 3.18 (0.83, 5.97) | 352.3% (214.51%, 565.15%) | 59.94 (19.8, 112.47) | 65.54% (13.82%, 149.47%) |
| Chongqing | Absolute number,  thousands | 5.09 (1.68, 9.87) | 78.84% (8.46%, 193.56%) | 117.19 (38, 235.41) | 40.61% (-14.81%, 136.34%) | 5 (1.39, 9.4) | 375.61% (194.09%, 678.69%) | 122.19 (39.56, 243.41) | 44.78% (-12.19%, 142.16%) |
| Fujian | Absolute number,  thousands | 4.57 (1.4, 9.91) | 8.75% (-32.68%, 90.34%) | 117.15 (34.88, 253.84) | -3.32% (-40.02%, 64.19%) | 4.98 (1.36, 9.6) | 226.21% (97.21%, 433.18%) | 122.13 (36.39, 261.31) | -0.47% (-37.75%, 69.22%) |
| Gansu | Absolute number,  thousands | 2.7 (0.76, 7.01) | 17.34% (-20.05%, 82.17%) | 65.99 (18.05, 171.35) | -3.92% (-35.8%, 47.27%) | 1.86 (0.52, 3.8) | 124.33% (55.36%, 246.28%) | 67.85 (18.62, 175.06) | -2.39% (-34.59%, 50.11%) |
| Guangdong | Absolute number,  thousands | 10.89 (3.7, 20.51) | 31.6% (-10.93%, 113.66%) | 282.3 (90.8, 540.66) | 22.25% (-17.93%, 99.22%) | 16.72 (4.34, 32.58) | 343.98% (193.95%, 535.27%) | 299.03 (94.95, 568.72) | 27.41% (-14.26%, 108.09%) |
| Guangxi | Absolute number,  thousands | 5.36 (1.78, 10.75) | 51.21% (4.29%, 117.09%) | 139.86 (43.7, 279.36) | 34.22% (-6.21%, 95.63%) | 5.75 (1.51, 11.04) | 266.84% (131.16%, 417.47%) | 145.62 (45.16, 290.17) | 37.67% (-3.86%, 100.97%) |
| Guizhou | Absolute number,  thousands | 4.54 (1.54, 9.08) | 37.94% (-6.87%, 104.58%) | 113.33 (36.91, 223.45) | 16.08% (-23.79%, 70.5%) | 3.32 (0.9, 6.33) | 160.4% (56.07%, 282.6%) | 116.65 (37.86, 229.67) | 17.95% (-22.87%, 73.66%) |
| Hainan | Absolute number,  thousands | 1.18 (0.38, 2.63) | 57.74% (6.8%, 151.03%) | 30.08 (8.96, 67.41) | 41.08% (-6.51%, 121.84%) | 1.03 (0.27, 2.03) | 234.55% (112.37%, 419.29%) | 31.11 (9.12, 69.17) | 43.84% (-4.49%, 125.83%) |
| Hebei | Absolute number,  thousands | 9.45 (2.75, 20.11) | 29.23% (-25.55%, 114.84%) | 231.85 (64.11, 494.9) | 14.83% (-35.16%, 87.51%) | 8.46 (2.12, 16.65) | 194.28% (81.84%, 356.25%) | 240.31 (65.85, 511.55) | 17.35% (-33.51%, 91.4%) |
| Heilongjiang | Absolute number,  thousands | 5.3 (1.82, 10.27) | 30.83% (-5.35%, 87.4%) | 130.21 (42.53, 257) | 5.54% (-24.46%, 51.61%) | 4.98 (1.29, 9.5) | 167.29% (77.12%, 280.57%) | 135.19 (43.78, 265.9) | 7.94% (-22.89%, 54.62%) |
| Henan | Absolute number,  thousands | 11.5 (3.23, 27.59) | -1.11% (-47.47%, 68.58%) | 264.47 (73.04, 630.44) | -16.48% (-55.56%, 41.11%) | 9.78 (2.32, 20.07) | 140.66% (38.67%, 273.95%) | 274.25 (76.06, 651.78) | -14.49% (-54.45%, 45.05%) |
| Hong Kong | Absolute number,  thousands | 1.37 (0.43, 2.43) | 78.88% (28.19%, 153.7%) | 28.25 (8.28, 50.53) | 45.71% (3.04%, 106.81%) | 1.51 (0.42, 2.82) | 226.21% (134.38%, 333.33%) | 29.76 (8.68, 53.36) | 49.92% (5.52%, 112.08%) |
| Hubei | Absolute number,  thousands | 7.52 (2.54, 15.41) | 15.69% (-21.16%, 78.58%) | 180.41 (58.83, 369.83) | -1.61% (-32.48%, 49.03%) | 9.42 (2.78, 18.06) | 231.25% (120.7%, 368.92%) | 189.83 (62.04, 386.97) | 1.94% (-30.22%, 53.21%) |
| Hunan | Absolute number,  thousands | 7.45 (2.78, 13.93) | 25.67% (-8.31%, 73.03%) | 181.51 (65.49, 342.82) | 2.63% (-25.92%, 41.8%) | 7.06 (1.96, 13.29) | 167.17% (80.46%, 276.7%) | 188.57 (67.81, 354.68) | 5.05% (-24.17%, 45.11%) |
| Inner Mongolia | Absolute number,  thousands | 3.07 (1.05, 6.2) | 27.52% (-19.78%, 101.01%) | 75.11 (24.51, 151.98) | 4.23% (-35.25%, 64.44%) | 3.36 (0.9, 6.32) | 232.71% (117.13%, 390.21%) | 78.47 (25.36, 157.24) | 7.39% (-33.35%, 69.98%) |
| Jiangsu | Absolute number,  thousands | 10.91 (3.34, 26.21) | 4.39% (-42.61%, 69.37%) | 240.11 (70.48, 572.41) | -14.8% (-54.34%, 37.9%) | 10.34 (2.96, 20.42) | 173.98% (69.86%, 316.87%) | 250.45 (74.01, 591.94) | -12.31% (-53.07%, 41.65%) |
| Jiangxi | Absolute number,  thousands | 4.79 (1.58, 10.15) | -7.06% (-32.97%, 32.55%) | 117.16 (37.61, 250.35) | -22.1% (-44.56%, 11.48%) | 4.25 (1.2, 8.37) | 122.5% (50.02%, 227.61%) | 121.42 (39.17, 257) | -20.29% (-43.27%, 13.93%) |
| Jilin | Absolute number,  thousands | 3.46 (1.2, 6.66) | 21.78% (-15.61%, 68.94%) | 85.94 (28.25, 167.48) | -0.64% (-33.24%, 39.13%) | 3.7 (0.91, 7.09) | 186.75% (77.04%, 306.56%) | 89.64 (29.15, 173.75) | 2.12% (-31.72%, 42.95%) |
| Liaoning | Absolute number,  thousands | 8.41 (2.9, 16.53) | 96.24% (39.6%, 176.42%) | 196.08 (64.61, 389.19) | 65.42% (15.61%, 135.4%) | 9.78 (2.68, 18.47) | 353.65% (222.76%, 530.01%) | 205.86 (67.51, 406.63) | 70.57% (19.53%, 143.12%) |
| Macao | Absolute number,  thousands | 0.08 (0.03, 0.14) | 100.71% (44.86%, 190%) | 1.84 (0.65, 3.24) | 88% (33.77%, 174.46%) | 0.08 (0.02, 0.14) | 303.06% (180.31%, 498.37%) | 1.91 (0.68, 3.37) | 92.05% (36.78%, 179.67%) |
| Ningxia | Absolute number,  thousands | 0.65 (0.18, 1.69) | 61.36% (2.43%, 144.26%) | 16.53 (4.51, 42.43) | 37.25% (-14.22%, 108.82%) | 0.6 (0.14, 1.2) | 295.75% (146.33%, 508.59%) | 17.13 (4.65, 43.35) | 40.45% (-12.38%, 113.74%) |
| Qinghai | Absolute number,  thousands | 0.71 (0.2, 1.83) | 55.73% (2.49%, 154.56%) | 18.94 (5.24, 49.43) | 30.84% (-15.47%, 113.82%) | 0.49 (0.13, 1.05) | 191.72% (84.29%, 344.9%) | 19.43 (5.4, 50.56) | 32.69% (-14.07%, 117.73%) |
| Shaanxi | Absolute number,  thousands | 3.84 (1.2, 8.79) | -1.52% (-42.45%, 67.05%) | 92.88 (28.07, 213.94) | -18.23% (-52.01%, 39.94%) | 3.27 (0.83, 6.31) | 131.14% (42.82%, 269.06%) | 96.15 (28.92, 219.16) | -16.4% (-50.76%, 42.88%) |
| Shandong | Absolute number,  thousands | 12.12 (3.83, 27.69) | 17.45% (-29.38%, 95.04%) | 285.9 (89.14, 666.94) | 0.06% (-41.88%, 64.51%) | 11.96 (3, 23.51) | 201.74% (82.34%, 366.28%) | 297.86 (93.48, 686.88) | 2.82% (-40.32%, 67.22%) |
| Shanghai | Absolute number,  thousands | 4.12 (1.39, 7.85) | 78.58% (24.71%, 179.94%) | 87.29 (27.89, 168.69) | 45.7% (2.5%, 127.78%) | 4.15 (1.13, 7.91) | 233.94% (130.22%, 398.51%) | 91.45 (28.95, 176.59) | 49.53% (5.06%, 133.1%) |
| Shanxi | Absolute number,  thousands | 4.45 (1.2, 10.72) | 11.78% (-34.25%, 76.17%) | 106.04 (28.86, 254.24) | -7.03% (-47.02%, 45.7%) | 3.73 (0.97, 7.22) | 152.69% (59.36%, 302.21%) | 109.77 (30.05, 260.18) | -4.99% (-45.77%, 48.23%) |
| Sichuan | Absolute number,  thousands | 13.78 (4.3, 28.96) | -14.21% (-45.24%, 32.04%) | 322.25 (98.59, 691.66) | -32.51% (-57.34%, 8.23%) | 11.64 (3.64, 21.97) | 95.47% (28.12%, 201.64%) | 333.88 (102.94, 709.85) | -30.93% (-56.21%, 10.6%) |
| Tianjin | Absolute number,  thousands | 1.67 (0.6, 3.18) | 97.84% (41.64%, 185.08%) | 38.25 (12.91, 73.45) | 69.39% (18.14%, 140.6%) | 2.08 (0.47, 4.15) | 333.57% (193.76%, 527.13%) | 40.33 (13.38, 77.76) | 74.88% (21.49%, 148.18%) |
| Xinjiang | Absolute number,  thousands | 1.98 (0.57, 4.43) | 33.03% (-19.64%, 131.34%) | 56.81 (15.86, 124.22) | 25.86% (-26.01%, 110.89%) | 1.48 (0.4, 2.88) | 180.3% (65.75%, 351.33%) | 58.3 (16.37, 127.01) | 27.65% (-25.03%, 113.98%) |
| Xizang | Absolute number,  thousands | 0.19 (0.05, 0.48) | -17.32% (-42.52%, 24.67%) | 5.91 (1.58, 14.8) | -17.26% (-42.6%, 25.28%) | 0.1 (0.02, 0.22) | 26% (-15.31%, 94.62%) | 6.02 (1.61, 15) | -16.78% (-42.1%, 25.85%) |
| Yunnan | Absolute number,  thousands | 4.5 (1.53, 8.82) | 51.71% (8.04%, 123.1%) | 117.56 (37.94, 232.9) | 32.9% (-7.46%, 97.25%) | 3.77 (1.1, 7.2) | 211.72% (106.86%, 366.41%) | 121.33 (39.12, 239.42) | 35.31% (-5.69%, 100.81%) |
| Zhejiang | Absolute number,  thousands | 9.91 (3.21, 19.83) | 56.89% (8.74%, 143.73%) | 231.93 (72.07, 468.39) | 36.92% (-7.5%, 111.8%) | 12.1 (3.4, 23.06) | 349.13% (185.27%, 550.46%) | 244.04 (75.86, 493.35) | 41.81% (-4.43%, 119.46%) |
| Anhui | Age-standardized  percent | 6.3% (1.89%, 14.4%) | -41.72% (-58%, -16.99%) | 6.02% (1.8%, 13.65%) | -39.99% (-57.89%, -13.56%) | 9.05% (3.01%, 15.96%) | -16.85% (-41.69%, 13.42%) | 6.1% (1.84%, 13.7%) | -39.26% (-57.46%, -12.44%) |
| Beijing | Age-standardized  percent | 6.65% (2.55%, 11.84%) | -25.35% (-40.68%, -3.36%) | 6.33% (2.29%, 11.27%) | -24.78% (-41%, -2.93%) | 8.74% (2.55%, 14.96%) | -1.19% (-24.29%, 18.22%) | 6.42% (2.31%, 11.34%) | -23.75% (-40.57%, -1.57%) |
| Chongqing | Age-standardized  percent | 5.84% (2.07%, 10.7%) | -45.12% (-61.98%, -18.77%) | 5.51% (1.89%, 10.25%) | -43.81% (-61.67%, -17.89%) | 9.21% (3.1%, 15.82%) | -14.41% (-43.54%, 10.01%) | 5.6% (1.92%, 10.35%) | -42.94% (-61.24%, -16.85%) |
| Fujian | Age-standardized  percent | 6.69% (2.2%, 14.29%) | -38.52% (-58.41%, -5.25%) | 6.39% (1.99%, 13.66%) | -36.31% (-57.27%, -0.34%) | 9.5% (3.13%, 16.5%) | -11.13% (-40.43%, 24.04%) | 6.48% (2.02%, 13.72%) | -35.5% (-56.66%, 0.96%) |
| Gansu | Age-standardized  percent | 6.97% (2%, 17.37%) | -30.89% (-46.32%, -6.63%) | 6.52% (1.82%, 16.07%) | -29.4% (-45.94%, -6.68%) | 8.55% (2.84%, 17.06%) | -14.37% (-35.51%, 11.38%) | 6.57% (1.85%, 16.09%) | -29.01% (-45.65%, -6.23%) |
| Guangdong | Age-standardized  percent | 6.68% (2.42%, 12.19%) | -29.66% (-47.43%, -4.14%) | 6.28% (2.18%, 11.53%) | -27.51% (-45.8%, -0.84%) | 10.08% (3.09%, 16.93%) | 0.94% (-27.74%, 22.53%) | 6.42% (2.22%, 11.72%) | -26.1% (-45%, 0.43%) |
| Guangxi | Age-standardized  percent | 6.71% (2.34%, 12.93%) | -23.37% (-39.6%, -7.7%) | 6.27% (2.06%, 12.09%) | -20.03% (-37.13%, -4.71%) | 9.77% (3.09%, 16.92%) | 5.16% (-28.05%, 26.49%) | 6.36% (2.08%, 12.21%) | -19.08% (-36.56%, -3.57%) |
| Guizhou | Age-standardized  percent | 7.6% (2.74%, 14.1%) | -25.21% (-39.86%, -10.33%) | 7.19% (2.51%, 13.33%) | -21.01% (-37.51%, -3.25%) | 9.94% (3.23%, 16.83%) | -5.54% (-37.52%, 17.33%) | 7.25% (2.52%, 13.4%) | -20.53% (-37.41%, -2.3%) |
| Hainan | Age-standardized  percent | 7.47% (2.56%, 15.25%) | -21.09% (-34.96%, -2.36%) | 7.12% (2.29%, 14.5%) | -17.76% (-33.07%, 0.6%) | 9.78% (3.15%, 16.97%) | 0.6% (-28.04%, 24.2%) | 7.19% (2.3%, 14.52%) | -17.14% (-33.1%, 1.55%) |
| Hebei | Age-standardized  percent | 6.5% (1.99%, 13.32%) | -38.4% (-58.84%, -8.43%) | 6.13% (1.81%, 12.75%) | -36.93% (-58.72%, -6.4%) | 8.91% (2.59%, 16.02%) | -14.74% (-45.17%, 12.5%) | 6.2% (1.82%, 12.8%) | -36.3% (-58.44%, -5.66%) |
| Heilongjiang | Age-standardized  percent | 5.64% (2.05%, 10.63%) | -33.43% (-45.33%, -17.35%) | 5.3% (1.86%, 10.17%) | -32.79% (-45.37%, -16.64%) | 8.63% (2.71%, 14.92%) | -7.38% (-33.99%, 11.33%) | 5.37% (1.88%, 10.28%) | -31.99% (-44.9%, -15.96%) |
| Henan | Age-standardized  percent | 6% (1.65%, 14%) | -47.21% (-68.31%, -12.88%) | 5.72% (1.57%, 13.17%) | -45.31% (-68.25%, -13.96%) | 8.41% (2.44%, 15.52%) | -23.37% (-51.5%, 8.57%) | 5.79% (1.59%, 13.2%) | -44.71% (-68.01%, -13.42%) |
| Hong Kong | Age-standardized  percent | 8.09% (2.68%, 13.59%) | -3.34% (-17.2%, 17.86%) | 7.69% (2.4%, 12.94%) | -2.74% (-18.04%, 16.35%) | 10.99% (3.28%, 18.34%) | 5.08% (-16.21%, 20.76%) | 7.81% (2.44%, 13.08%) | -1.79% (-17.02%, 17.19%) |
| Hubei | Age-standardized  percent | 6.02% (2.12%, 12.31%) | -37% (-54.17%, -16.98%) | 5.76% (1.96%, 11.68%) | -34.65% (-52.85%, -14.68%) | 9.44% (3.11%, 16.23%) | -6.59% (-33.86%, 14.78%) | 5.87% (2%, 11.8%) | -33.5% (-52.04%, -13.57%) |
| Hunan | Age-standardized  percent | 6.31% (2.36%, 11.77%) | -29.02% (-41.16%, -17.78%) | 5.95% (2.16%, 11.09%) | -25.4% (-38.53%, -13.1%) | 8.99% (2.82%, 15.51%) | -3.53% (-29.45%, 13.54%) | 6.03% (2.19%, 11.19%) | -24.62% (-38.04%, -12.06%) |
| Inner Mongolia | Age-standardized  percent | 5.91% (2.1%, 11.8%) | -41.37% (-57.49%, -17.47%) | 5.55% (1.87%, 11.15%) | -39.26% (-56.98%, -14.07%) | 9.3% (2.93%, 16.21%) | -9.57% (-35.85%, 11.77%) | 5.65% (1.9%, 11.26%) | -38.3% (-56.33%, -12.84%) |
| Jiangsu | Age-standardized  percent | 5.36% (1.71%, 12.29%) | -44.67% (-67.33%, -13.39%) | 5.19% (1.56%, 11.76%) | -41.96% (-65.4%, -14.29%) | 7.94% (2.45%, 14.59%) | -17.27% (-45.98%, 13.7%) | 5.26% (1.59%, 11.81%) | -41.17% (-64.83%, -13.17%) |
| Jiangxi | Age-standardized  percent | 6.48% (2.33%, 13.01%) | -32.31% (-45.89%, -17.22%) | 6.11% (2.1%, 12.33%) | -29.62% (-43.66%, -13.43%) | 9.16% (3.24%, 15.59%) | -7.69% (-32.57%, 16.17%) | 6.18% (2.12%, 12.41%) | -28.91% (-43.3%, -11.99%) |
| Jilin | Age-standardized  percent | 6.37% (2.24%, 11.85%) | -30.53% (-44.44%, -16.91%) | 6.04% (2.01%, 11.32%) | -29.26% (-44.81%, -14.96%) | 9.37% (2.72%, 16.15%) | -5.65% (-37.17%, 15.59%) | 6.13% (2.04%, 11.43%) | -28.37% (-44.51%, -13.55%) |
| Liaoning | Age-standardized  percent | 6.54% (2.27%, 12.59%) | -25.79% (-40.77%, -8.3%) | 6.17% (2.05%, 12.01%) | -24.26% (-40.21%, -6.65%) | 9.91% (2.95%, 17%) | 4.65% (-22.05%, 25.73%) | 6.28% (2.08%, 12.11%) | -23.09% (-39.8%, -5.19%) |
| Macao | Age-standardized  percent | 9.01% (3.5%, 14.57%) | -2.91% (-20.87%, 26.94%) | 8.52% (3.21%, 13.99%) | -2.74% (-21.65%, 29.06%) | 10.49% (4.15%, 16.58%) | -0.6% (-23.85%, 27.19%) | 8.58% (3.24%, 14.07%) | -2.32% (-21.63%, 29.71%) |
| Ningxia | Age-standardized  percent | 6.18% (1.99%, 15.39%) | -34.04% (-50.87%, -14.22%) | 5.85% (1.78%, 14.28%) | -31.3% (-49.69%, -9.98%) | 8.61% (2.71%, 15.88%) | -8.84% (-37.95%, 18.03%) | 5.92% (1.8%, 14.33%) | -30.61% (-49.17%, -9.05%) |
| Qinghai | Age-standardized  percent | 6.81% (2.01%, 16.81%) | -30.96% (-48.99%, -6.73%) | 6.38% (1.85%, 15.73%) | -28.72% (-48.05%, -5.93%) | 9.04% (2.67%, 17.94%) | -10.26% (-36.14%, 12.91%) | 6.43% (1.87%, 15.77%) | -28.28% (-47.76%, -5.21%) |
| Shaanxi | Age-standardized  percent | 5.74% (1.82%, 12.83%) | -42.18% (-61.52%, -19.21%) | 5.37% (1.64%, 11.9%) | -39.66% (-60.66%, -16.78%) | 8.01% (2.49%, 14.77%) | -18.17% (-46.03%, 7.1%) | 5.43% (1.66%, 11.99%) | -39.05% (-60.24%, -15.95%) |
| Shandong | Age-standardized  percent | 5.65% (1.84%, 12.84%) | -41.21% (-60.78%, -18.65%) | 5.4% (1.66%, 12.19%) | -38.76% (-60.24%, -15.47%) | 8.67% (2.63%, 15.61%) | -12.39% (-42.98%, 12.94%) | 5.49% (1.68%, 12.27%) | -37.92% (-59.77%, -14.03%) |
| Shanghai | Age-standardized  percent | 7.4% (2.57%, 13.64%) | -15.47% (-33%, 9.14%) | 6.98% (2.32%, 13.02%) | -15.38% (-30.76%, 7.33%) | 9.83% (3.25%, 16.79%) | -1.31% (-22.28%, 20.52%) | 7.07% (2.35%, 13.09%) | -14.57% (-30.52%, 8.39%) |
| Shanxi | Age-standardized  percent | 6.38% (1.97%, 14.71%) | -38.9% (-59.26%, -13.4%) | 5.99% (1.8%, 14%) | -38.06% (-58.53%, -13.63%) | 8.59% (2.78%, 15.67%) | -16.49% (-40.98%, 6.04%) | 6.05% (1.83%, 14.04%) | -37.47% (-58.06%, -13.03%) |
| Sichuan | Age-standardized  percent | 6.37% (2.16%, 12.59%) | -42.93% (-57.03%, -18.63%) | 5.95% (1.96%, 11.72%) | -41.01% (-57.1%, -19.31%) | 9.04% (3.13%, 15.51%) | -17.94% (-41.13%, 4.28%) | 6.02% (1.98%, 11.79%) | -40.37% (-56.71%, -18.89%) |
| Tianjin | Age-standardized  percent | 5.53% (1.94%, 10.48%) | -27.38% (-43.18%, -9.98%) | 5.29% (1.76%, 10.14%) | -24.63% (-41.12%, -8.8%) | 8.47% (2.26%, 14.62%) | -1.19% (-27.28%, 19.81%) | 5.39% (1.78%, 10.28%) | -23.46% (-41.27%, -7.16%) |
| Xinjiang | Age-standardized  percent | 6.31% (2.03%, 13.43%) | -36.14% (-55.93%, -9.15%) | 5.93% (1.81%, 12.39%) | -33.96% (-55.99%, -7.4%) | 7.88% (2.62%, 14.22%) | -17.32% (-46.23%, 12.15%) | 5.96% (1.82%, 12.4%) | -33.58% (-55.79%, -7.23%) |
| Xizang | Age-standardized  percent | 6.86% (1.94%, 17.11%) | -27.08% (-42.59%, -9.76%) | 6.34% (1.76%, 15.67%) | -26.17% (-41.18%, -8.37%) | 7.97% (2.28%, 16.22%) | -18.11% (-39.79%, 5.19%) | 6.36% (1.77%, 15.68%) | -26% (-41.07%, -8.13%) |
| Yunnan | Age-standardized  percent | 7.11% (2.67%, 13.5%) | -26.28% (-38.3%, -13.82%) | 6.64% (2.4%, 12.9%) | -21.92% (-34.05%, -7.18%) | 9.63% (3.29%, 16.23%) | -3.38% (-26.43%, 15.92%) | 6.7% (2.42%, 12.95%) | -21.33% (-33.99%, -6.07%) |
| Zhejiang | Age-standardized  percent | 6.96% (2.51%, 13.95%) | -27.26% (-40.5%, -9.72%) | 6.79% (2.36%, 13.66%) | -24.1% (-38.16%, -4.56%) | 10.42% (3.57%, 17.69%) | 1.66% (-29.75%, 26.91%) | 6.91% (2.4%, 13.71%) | -22.93% (-37.59%, -3%) |
| Anhui | Age-standardized rate,  per 100,000 people | 9.03 (2.45, 20.25) | -65.25% (-76.6%, -46.2%) | 209.67 (55.54, 467.36) | -67.97% (-78.92%, -50.46%) | 8.44 (2.2, 15.92) | -2.16% (-36.89%, 48.3%) | 218.11 (57.55, 483.87) | -67.11% (-78.29%, -49.12%) |
| Beijing | Age-standardized rate,  per 100,000 people | 8.23 (2.87, 15.22) | -46.52% (-62.85%, -19.16%) | 174.79 (58.68, 329.48) | -51.54% (-66.66%, -27.14%) | 9.64 (2.55, 18.07) | 33.75% (-7.8%, 92.35%) | 184.43 (61.38, 345.18) | -49.87% (-65.53%, -24.51%) |
| Chongqing | Age-standardized rate,  per 100,000 people | 9.49 (3.15, 18.41) | -60.6% (-75.7%, -35.8%) | 221.97 (71.52, 444.74) | -64.66% (-78.98%, -41.97%) | 9.36 (2.53, 17.86) | 15.14% (-30.22%, 82.33%) | 231.34 (74.21, 459.86) | -63.64% (-78.25%, -40.33%) |
| Fujian | Age-standardized rate,  per 100,000 people | 8.6 (2.68, 18.53) | -60.48% (-75.06%, -31.71%) | 209.36 (63.1, 451.31) | -62.63% (-76.57%, -36.39%) | 8.83 (2.44, 16.99) | 21.71% (-25.57%, 98.84%) | 218.19 (65.81, 463.79) | -61.56% (-75.83%, -34.49%) |
| Gansu | Age-standardized rate,  per 100,000 people | 7.9 (2.26, 20.15) | -56.27% (-69.27%, -34.47%) | 183.58 (51.11, 473.16) | -59.36% (-72.48%, -37.77%) | 5.15 (1.43, 10.49) | -10.62% (-37.94%, 32.87%) | 188.73 (52.71, 484.5) | -58.75% (-71.97%, -36.63%) |
| Guangdong | Age-standardized rate,  per 100,000 people | 8.3 (2.87, 15.44) | -55.27% (-69.49%, -29.54%) | 195.53 (64.22, 371.66) | -58.59% (-72.04%, -32.75%) | 11.59 (3.13, 22.33) | 48.2% (-0.32%, 111.53%) | 207.12 (67.21, 392.88) | -56.85% (-70.79%, -29.73%) |
| Guangxi | Age-standardized rate,  per 100,000 people | 8.02 (2.68, 16.1) | -34.92% (-55%, -6.88%) | 210.11 (65.52, 419.45) | -36.38% (-55.44%, -8.03%) | 8.58 (2.25, 16.46) | 68.4% (3.88%, 137.73%) | 218.69 (67.71, 435.63) | -34.79% (-54.34%, -5.55%) |
| Guizhou | Age-standardized rate,  per 100,000 people | 9.62 (3.33, 19.18) | -41.74% (-60.42%, -16.44%) | 232.57 (76.77, 457.34) | -45.71% (-63.98%, -19.93%) | 6.75 (1.84, 12.81) | 16.2% (-29.35%, 67.91%) | 239.32 (78.71, 468.34) | -44.88% (-63.47%, -18.91%) |
| Hainan | Age-standardized rate,  per 100,000 people | 9.54 (3.11, 21.24) | -44.06% (-61.47%, -13.47%) | 234.38 (70.75, 523.46) | -46.82% (-64.37%, -17.12%) | 8.02 (2.14, 15.72) | 23.25% (-21.3%, 91.82%) | 242.4 (72.1, 537.08) | -45.8% (-63.65%, -15.8%) |
| Hebei | Age-standardized rate,  per 100,000 people | 8.92 (2.61, 18.71) | -47.44% (-68.59%, -15.47%) | 211.52 (58.54, 447.32) | -49.11% (-70.72%, -16.37%) | 7.62 (1.94, 14.99) | 25.8% (-24.06%, 94.75%) | 219.15 (60.17, 460.65) | -48.03% (-70.04%, -15.05%) |
| Heilongjiang | Age-standardized rate,  per 100,000 people | 9.8 (3.45, 18.73) | -52.69% (-65.53%, -34.46%) | 229.53 (76.62, 448.43) | -56.09% (-68.69%, -37.65%) | 8.71 (2.28, 16.49) | 6.33% (-32.89%, 49.11%) | 238.24 (78.84, 463.92) | -55.13% (-68.01%, -36.24%) |
| Henan | Age-standardized rate,  per 100,000 people | 8.37 (2.36, 20.03) | -57.29% (-76.08%, -29.62%) | 189.7 (52.52, 450.1) | -60.7% (-79%, -34.32%) | 6.97 (1.62, 14.23) | 9.58% (-36.48%, 65.41%) | 196.66 (54.48, 464.88) | -59.78% (-78.49%, -32.57%) |
| Hong Kong | Age-standardized rate,  per 100,000 people | 8.44 (2.64, 14.97) | -39.83% (-56.28%, -17.14%) | 192.52 (54.35, 343.28) | -42.52% (-58.9%, -20.01%) | 10.34 (2.73, 19.48) | 28.02% (-10.33%, 70.38%) | 202.86 (56.79, 364.43) | -40.86% (-57.76%, -17.85%) |
| Hubei | Age-standardized rate,  per 100,000 people | 8.4 (2.86, 17.08) | -53.95% (-67.78%, -31.2%) | 195.11 (64.18, 396.8) | -56.59% (-69.84%, -35.02%) | 10.07 (2.96, 19.37) | 41.03% (-7.34%, 99.96%) | 205.18 (67.61, 414.85) | -55.06% (-68.81%, -33.06%) |
| Hunan | Age-standardized rate,  per 100,000 people | 7.29 (2.72, 13.59) | -48.11% (-61.75%, -29.92%) | 178.46 (64.52, 334.92) | -51.04% (-64.9%, -32.47%) | 6.86 (1.89, 12.87) | 22.39% (-18.74%, 70.81%) | 185.32 (66.68, 346.32) | -49.92% (-64.16%, -30.96%) |
| Inner Mongolia | Age-standardized rate,  per 100,000 people | 8.72 (3.04, 17.41) | -57.39% (-72.31%, -34.73%) | 197.64 (65.3, 396.71) | -60.78% (-75.39%, -38.71%) | 8.74 (2.37, 16.35) | 19.12% (-23.3%, 71.93%) | 206.39 (67.54, 410.6) | -59.63% (-74.76%, -36.86%) |
| Jiangsu | Age-standardized rate,  per 100,000 people | 7.76 (2.41, 18.42) | -60.83% (-77.07%, -37.42%) | 171.7 (50.58, 405.12) | -63.91% (-80.52%, -42.57%) | 7.32 (2.07, 14.49) | 12.1% (-31.3%, 69.92%) | 179.02 (53.03, 419.3) | -62.88% (-80%, -41.07%) |
| Jiangxi | Age-standardized rate,  per 100,000 people | 8.17 (2.72, 17.22) | -62.7% (-72.81%, -47.43%) | 192.91 (62.56, 409.64) | -65.91% (-75.65%, -51.67%) | 6.94 (1.96, 13.65) | -6.48% (-37.85%, 37.19%) | 199.85 (65.13, 419.8) | -65.14% (-75.22%, -50.37%) |
| Jilin | Age-standardized rate,  per 100,000 people | 8.89 (3.17, 16.93) | -53.69% (-67.75%, -36.42%) | 209.46 (69.52, 406.73) | -56.57% (-71.2%, -39.09%) | 8.87 (2.2, 16.86) | 19.09% (-28.41%, 68.98%) | 218.33 (71.69, 423.13) | -55.42% (-70.5%, -37.35%) |
| Liaoning | Age-standardized rate,  per 100,000 people | 11.09 (3.86, 21.58) | -29.02% (-48.72%, -0.49%) | 252.91 (83.32, 497.21) | -31.74% (-51.97%, -2.87%) | 12.35 (3.32, 23.47) | 80.84% (27.24%, 150.76%) | 265.26 (86.95, 519.09) | -29.7% (-50.47%, 0.24%) |
| Macao | Age-standardized rate,  per 100,000 people | 8.24 (3.23, 14.47) | -41.07% (-57.91%, -14.88%) | 185.88 (67.36, 327.13) | -45.94% (-61.41%, -22.2%) | 7.7 (2.61, 13.71) | 15.88% (-19.9%, 69.56%) | 193.57 (70.33, 340.71) | -44.77% (-60.54%, -20.27%) |
| Ningxia | Age-standardized rate,  per 100,000 people | 8.19 (2.41, 21.3) | -54.1% (-70.16%, -31.79%) | 188.24 (52.28, 482.23) | -57.61% (-73.43%, -34.99%) | 6.71 (1.69, 13.24) | 15.05% (-27.83%, 74.82%) | 194.95 (54.11, 492.78) | -56.67% (-72.82%, -33.61%) |
| Qinghai | Age-standardized rate,  per 100,000 people | 11.19 (3.27, 28.58) | -43.69% (-61.32%, -7.59%) | 266.86 (75.43, 692.43) | -48.69% (-66.25%, -16.26%) | 6.88 (1.86, 14.64) | 7.16% (-31.2%, 61.29%) | 273.74 (77.83, 708.73) | -48.01% (-65.76%, -14.84%) |
| Shaanxi | Age-standardized rate,  per 100,000 people | 6.88 (2.15, 15.54) | -62.66% (-77.21%, -35.89%) | 159.3 (47.96, 362.99) | -65.67% (-79.77%, -42.12%) | 5.54 (1.41, 10.71) | -7.53% (-43.46%, 43.61%) | 164.85 (49.39, 371.82) | -64.93% (-79.31%, -40.97%) |
| Shandong | Age-standardized rate,  per 100,000 people | 7.63 (2.44, 17.32) | -54.06% (-72.02%, -25%) | 178.54 (55.41, 413.23) | -56.46% (-74.33%, -27.71%) | 7.4 (1.84, 14.54) | 26.76% (-23.52%, 89.96%) | 185.94 (58.04, 425.6) | -55.29% (-73.62%, -26.5%) |
| Shanghai | Age-standardized rate,  per 100,000 people | 10.05 (3.4, 19.22) | -40.99% (-58.69%, -11.45%) | 212.24 (67.96, 411.14) | -46.83% (-62.45%, -18.69%) | 9.94 (2.65, 18.84) | 19.88% (-17.52%, 77.44%) | 222.18 (70.52, 428.72) | -45.47% (-61.53%, -16.76%) |
| Shanxi | Age-standardized rate,  per 100,000 people | 9.14 (2.48, 21.62) | -56.05% (-73.17%, -32.78%) | 205.55 (55.82, 487.83) | -60.32% (-76.93%, -38.53%) | 7.17 (1.88, 13.97) | 3.73% (-34.61%, 62.81%) | 212.73 (58.19, 500.98) | -59.48% (-76.44%, -37.59%) |
| Sichuan | Age-standardized rate,  per 100,000 people | 9.83 (3.08, 20.66) | -51.32% (-67.6%, -22.26%) | 234.95 (72.11, 502.92) | -55.54% (-71.43%, -27.54%) | 8.38 (2.57, 15.97) | 22.49% (-19.43%, 87.7%) | 243.34 (75.17, 516.14) | -54.54% (-70.83%, -25.97%) |
| Tianjin | Age-standardized rate,  per 100,000 people | 8.01 (2.9, 15.16) | -36.05% (-54.34%, -9.07%) | 177.08 (59.87, 338.97) | -38.55% (-56.48%, -13.62%) | 9.44 (2.1, 18.97) | 53.82% (3.04%, 122.64%) | 186.52 (61.98, 358.17) | -36.62% (-55.53%, -10.32%) |
| Xinjiang | Age-standardized rate,  per 100,000 people | 7.46 (2.24, 16.48) | -57.01% (-73.18%, -26.83%) | 189.94 (54.3, 418.97) | -58.34% (-75.06%, -29.42%) | 4.94 (1.39, 9.65) | -10.9% (-45.96%, 43.51%) | 194.88 (56.07, 428.19) | -57.77% (-74.74%, -28.71%) |
| Xizang | Age-standardized rate,  per 100,000 people | 5.96 (1.62, 15.19) | -61.75% (-73.51%, -43.5%) | 162.2 (43.66, 411.37) | -63.26% (-74.47%, -45.4%) | 2.88 (0.72, 6.16) | -44.92% (-61.62%, -16.96%) | 165.08 (44.57, 417.69) | -63.05% (-74.13%, -45.37%) |
| Yunnan | Age-standardized rate,  per 100,000 people | 7.71 (2.69, 15.09) | -39.18% (-56.28%, -13.55%) | 190.08 (62.24, 374.04) | -42.33% (-59.36%, -15.21%) | 6.06 (1.8, 11.44) | 29.31% (-13.03%, 90.96%) | 196.14 (64.23, 384.87) | -41.33% (-58.71%, -13.82%) |
| Zhejiang | Age-standardized rate,  per 100,000 people | 10.52 (3.48, 21.01) | -45.59% (-61.27%, -18.72%) | 234.78 (73.71, 472.53) | -49.37% (-65.42%, -22.86%) | 12.13 (3.44, 23.15) | 61.69% (3.33%, 132.92%) | 246.91 (77.58, 496.46) | -47.6% (-64.25%, -19.96%) |

**References**

1. Cuschieri, S., *The STROBE guidelines.* Saudi J Anaesth, 2019. **13**(Suppl 1): p. S31-s34.

2. Shi, W., et al., *Red meat consumption, cardiovascular diseases, and diabetes: a systematic review and meta-analysis.* Eur Heart J, 2023. **44**(28): p. 2626-2635.

3. Veettil, S.K., et al., *Role of Diet in Colorectal Cancer Incidence: Umbrella Review of Meta-analyses of Prospective Observational Studies.* JAMA Netw Open, 2021. **4**(2): p. e2037341.

4. Abdelhamid, A.S., et al., *Omega-3 fatty acids for the primary and secondary prevention of cardiovascular disease.* Cochrane Database Syst Rev, 2020. **3**(3): p. Cd003177.

5. Houston, L., et al., *Tree Nut and Peanut Consumption and Risk of Cardiovascular Disease: A Systematic Review and Meta-Analysis of Randomized Controlled Trials.* Adv Nutr, 2023. **14**(5): p. 1029-1049.

6. Hooper, L., et al., *Reduction in saturated fat intake for cardiovascular disease.* Cochrane Database Syst Rev, 2020. **8**(8): p. Cd011737.

7. Chareonrungrueangchai, K., et al., *Dietary Factors and Risks of Cardiovascular Diseases: An Umbrella Review.* Nutrients, 2020. **12**(4).

8. Luo, C., et al., *Nut consumption and risk of type 2 diabetes, cardiovascular disease, and all-cause mortality: a systematic review and meta-analysis.* Am J Clin Nutr, 2014. **100**(1): p. 256-69.

9. Boushey, C., et al., *USDA Nutrition Evidence Systematic Reviews*, in *Dietary Patterns and All-Cause Mortality: A Systematic Review*. 2020, USDA Nutrition Evidence Systematic Review: Alexandria (VA).

10. Qin, X., et al., *Dietary Factors and Pancreatic Cancer Risk: An Umbrella Review of Meta-Analyses of Prospective Observational Studies.* Adv Nutr, 2023. **14**(3): p. 451-464.

11. Imamura, F., et al., *Consumption of sugar sweetened beverages, artificially sweetened beverages, and fruit juice and incidence of type 2 diabetes: systematic review, meta-analysis, and estimation of population attributable fraction.* Bmj, 2015. **351**: p. h3576.

12. Yin, J., et al., *Intake of Sugar-Sweetened and Low-Calorie Sweetened Beverages and Risk of Cardiovascular Disease: A Meta-Analysis and Systematic Review.* Adv Nutr, 2021. **12**(1): p. 89-101.

13. Aburto, N.J., et al., *Effect of increased potassium intake on cardiovascular risk factors and disease: systematic review and meta-analyses.* Bmj, 2013. **346**: p. f1378.

14. D'Elia, L., et al., *Habitual salt intake and risk of gastric cancer: a meta-analysis of prospective studies.* Clin Nutr, 2012. **31**(4): p. 489-98.

15. Kliemann, N., et al., *Food processing and cancer risk in Europe: results from the prospective EPIC cohort study.* Lancet Planet Health, 2023. **7**(3): p. e219-e232.

16. Micha, R., S.K. Wallace, and D. Mozaffarian, *Red and processed meat consumption and risk of incident coronary heart disease, stroke, and diabetes mellitus: a systematic review and meta-analysis.* Circulation, 2010. **121**(21): p. 2271-83.

17. Djuricic, I. and P.C. Calder, *Beneficial Outcomes of Omega-6 and Omega-3 Polyunsaturated Fatty Acids on Human Health: An Update for 2021.* Nutrients, 2021. **13**(7).
