# Supplementary material for: Dietary Risk Factors and Associated Disease Burden Among Chinese Adults Aged 25 Years and Older: Systematic Analysis of the Global Burden of Disease Study 2021
Source: JMIR Public Health Surveill. 2025 Aug 25;11:e72978. doi: 10.2196/72978 (PMC12441878; doi:10.2196/72978)

###### Figure S1: Deaths number and ASR-Deaths (A), YLLs number and ASR-YLLs (B), YLDs number and ASR-YLDs (C) and DALYs number and ASR-DALYs (D) of all causes attributable to dietary risk factors by sex in China, 1990 - 2021.


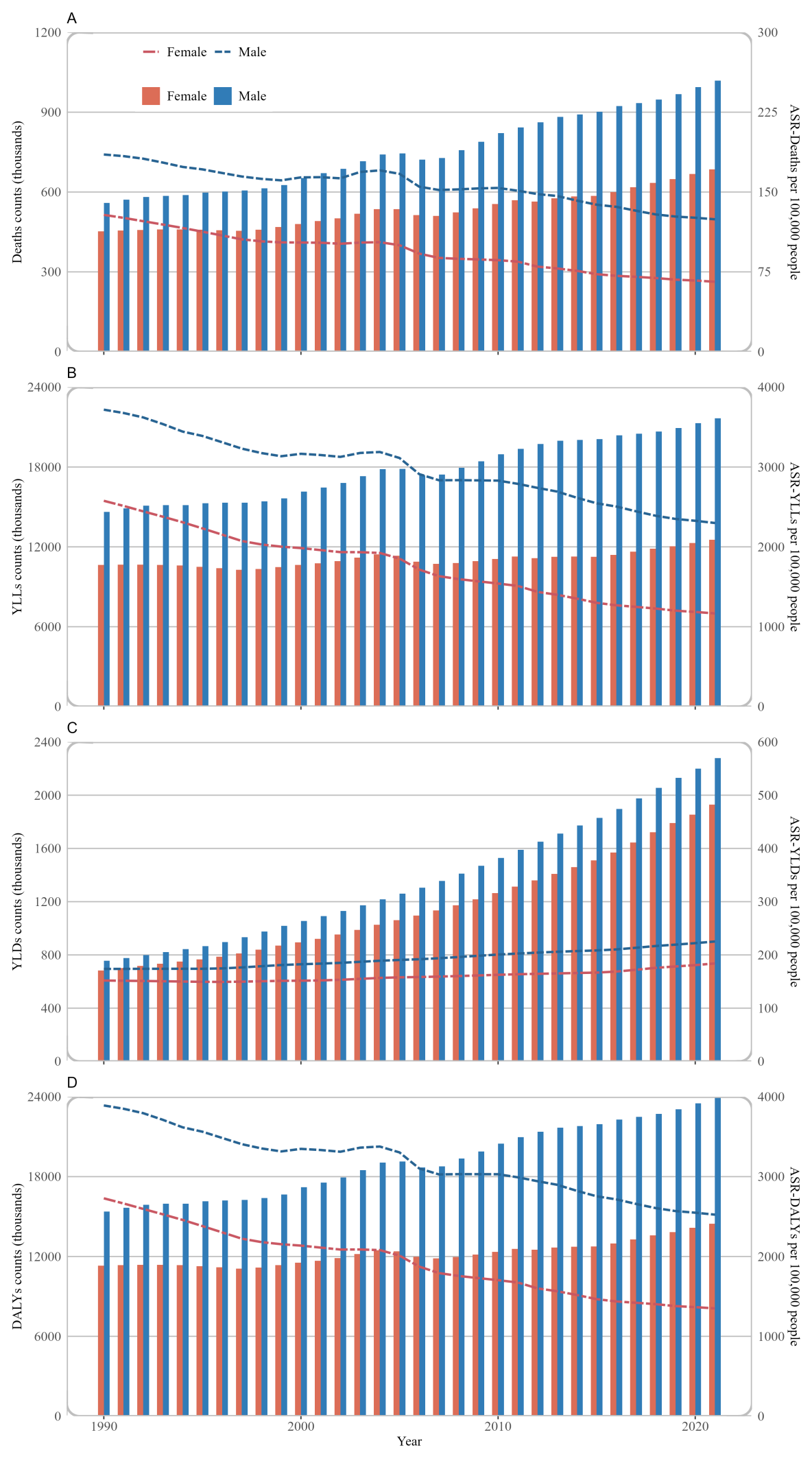


**Figure S2: Deaths number and age-specific deaths rate (A), YLLs number and age-specific YLLs rate (B), YLDs number and age-specific YLDs rate (C) and DALYs number and age-specific DALYs rate (D) of all causes attributable to dietary risk factors by sex in 2021, in China.**


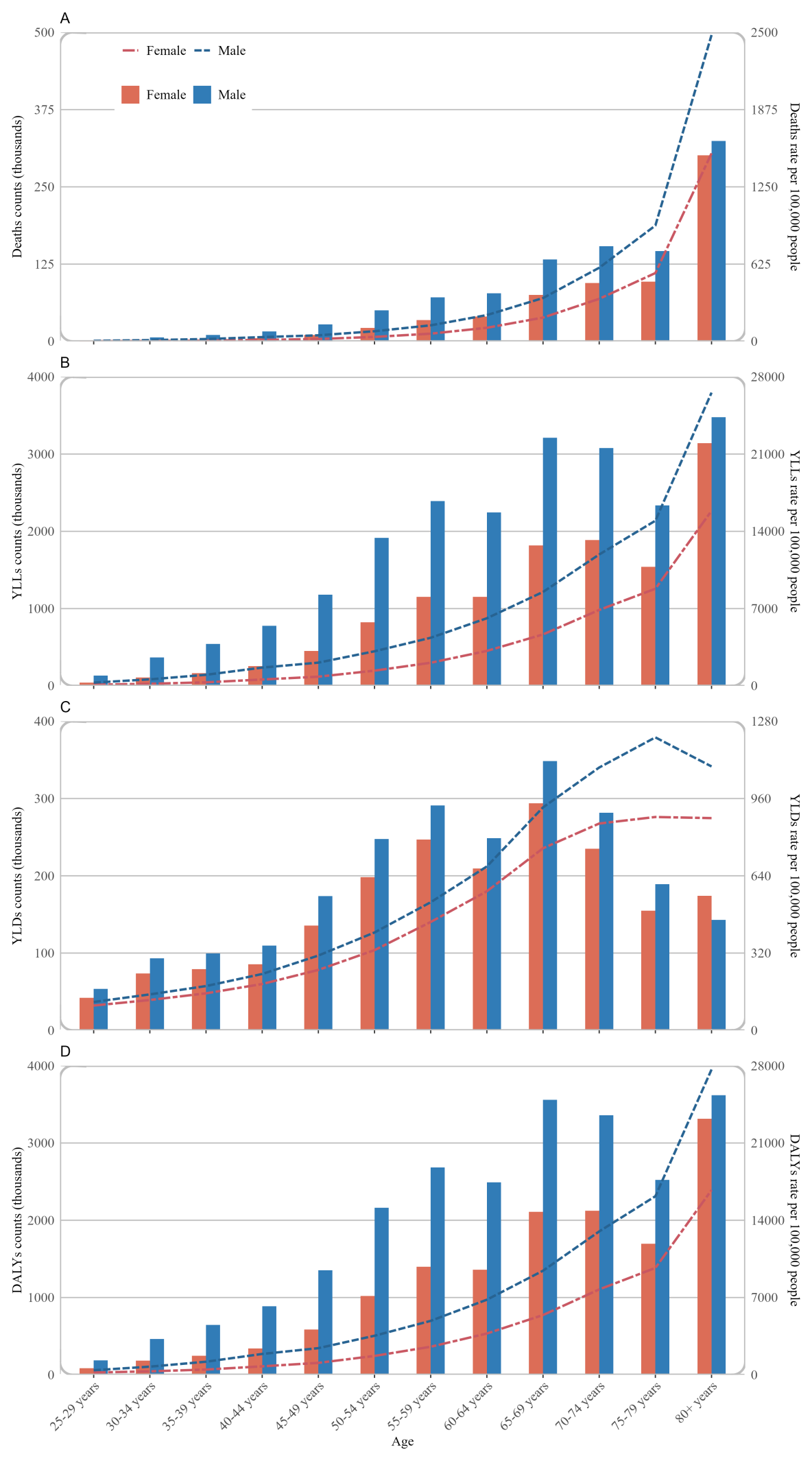


###### Figure S3: Deaths number and age-specific deaths rate (A), YLLs number and age-specific YLLs rate (B), YLDs number and age-specific YLDs rate (C) and DALYs number and age-specific DALYs rate (D) of cardiovascular diseases attributable to dietary risk factors by sex in 2021, in China.


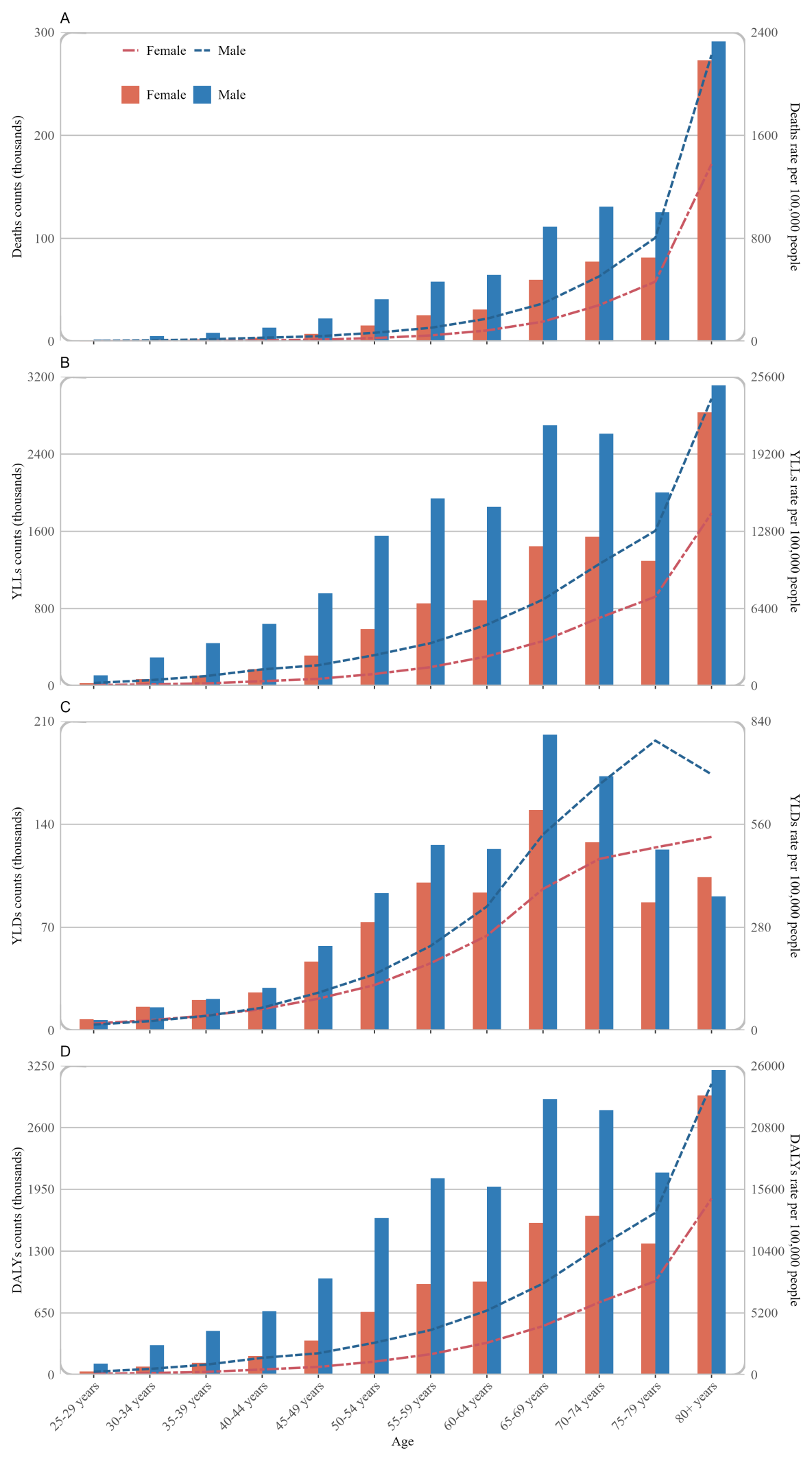


###### Figure S4: Deaths number and age-specific deaths rate (A), YLLs number and age-specific YLLs rate (B), YLDs number and age-specific YLDs rate (C) and DALYs number and age-specific DALYs rate (D) of diabetes and kidney diseases attributable to dietary risk factors by sex in 2021, in China.


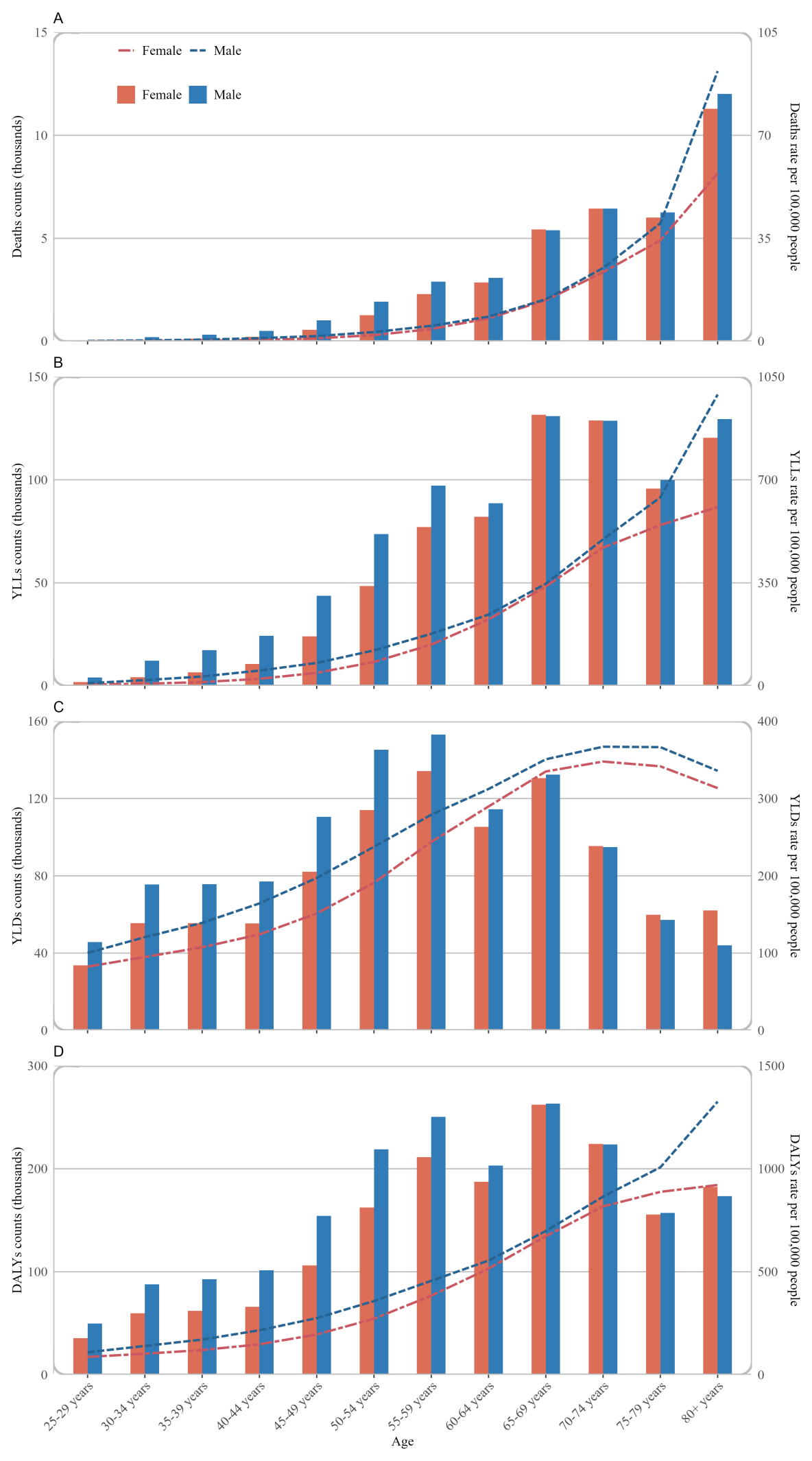


###### Figure S5: Deaths number and age-specific deaths rate (A), YLLs number and age-specific YLLs rate (B), YLDs number and age-specific YLDs rate (C) and DALYs number and age-specific DALYs rate (D) of neoplasms attributable to dietary risk factors by sex in 2021, in China.


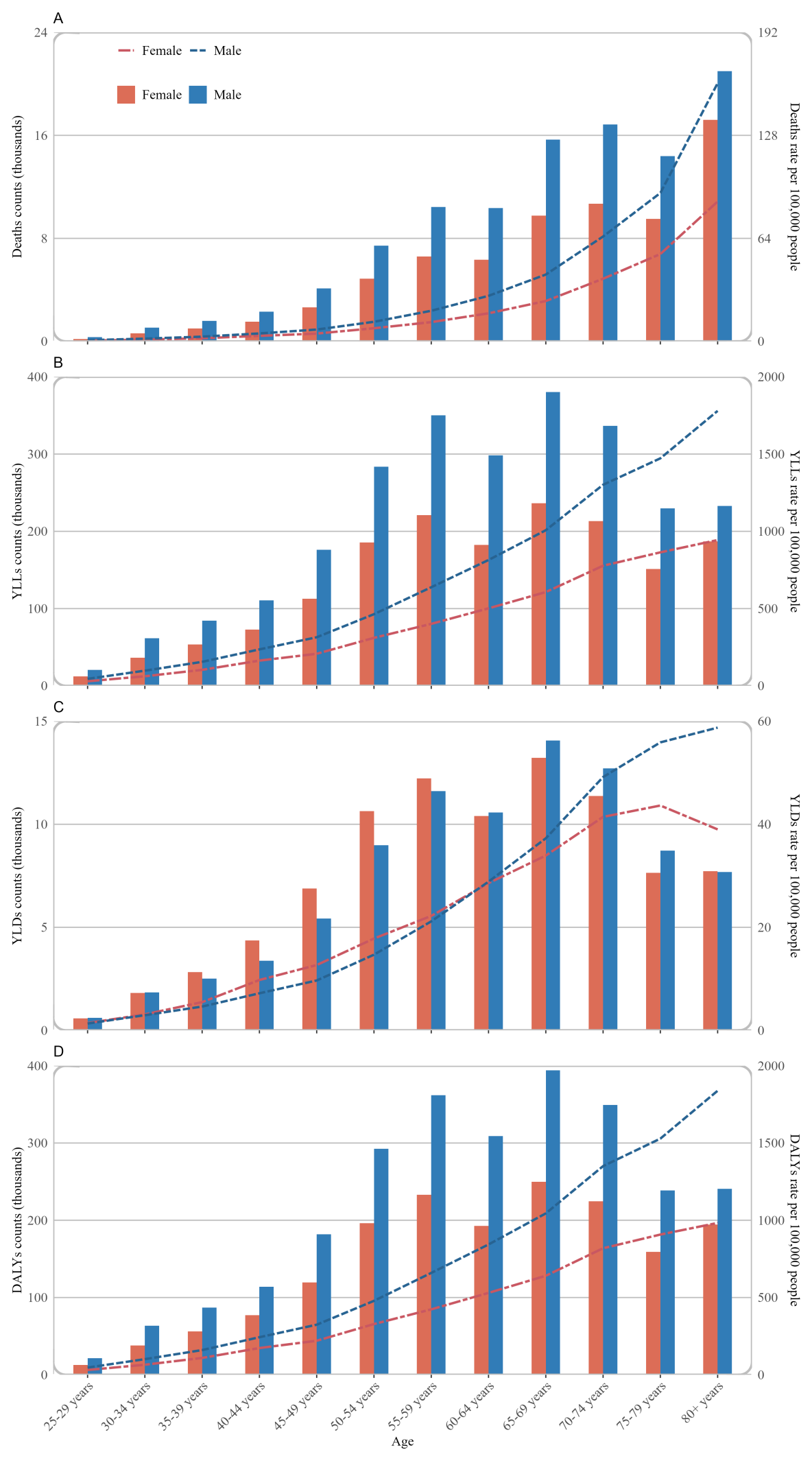


###### Figure S6: Burden of all causes (A), cardiovascular diseases (B), diabetes and kidney diseases (C), and neoplasms (D) attributable to dietary risk factors for female by region in 2021, in China.


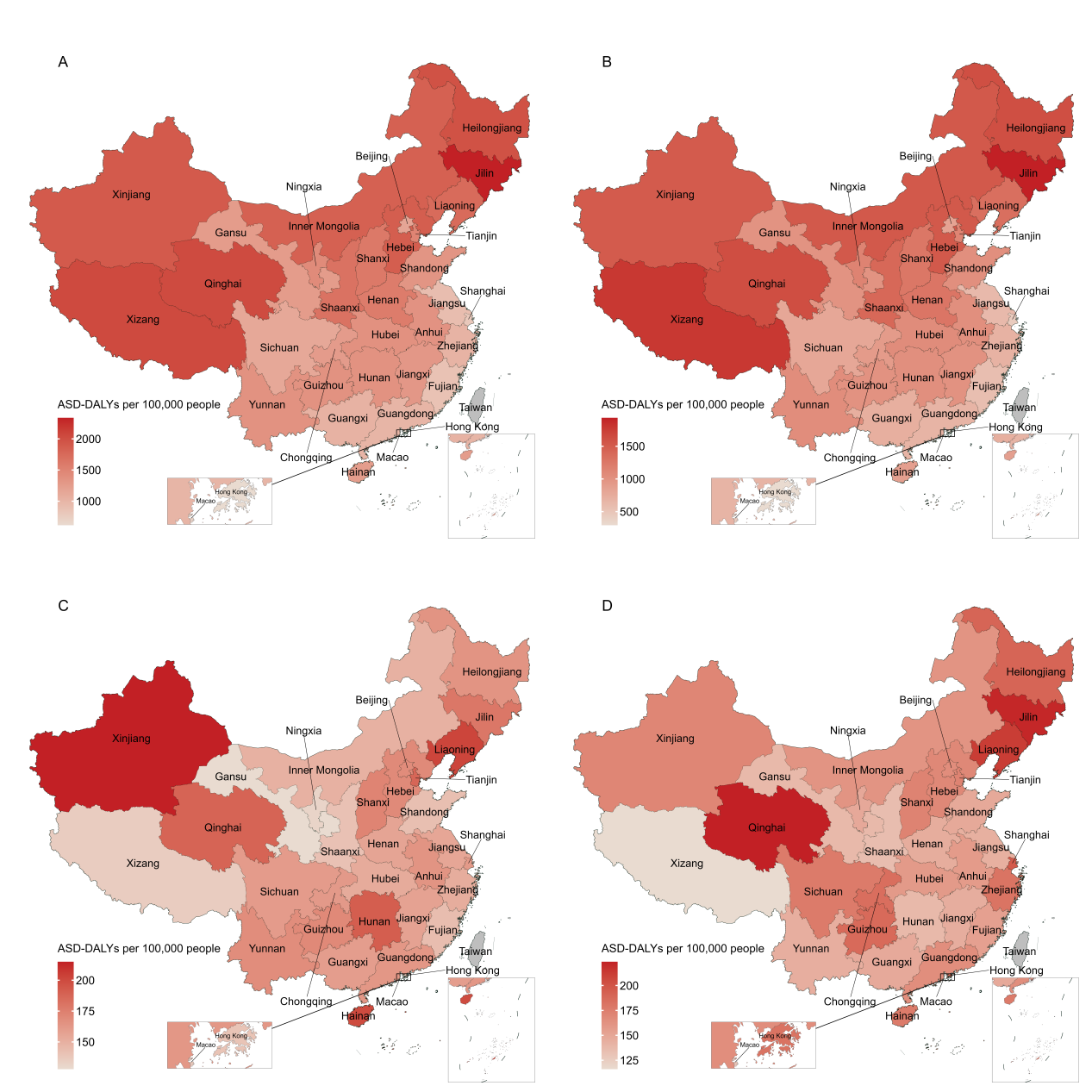


###### Figure S7: Burden of all causes (A), cardiovascular diseases (B), diabetes and kidney diseases (C), and neoplasms (D) attributable to dietary risk factors for male by region in 2021, in China.


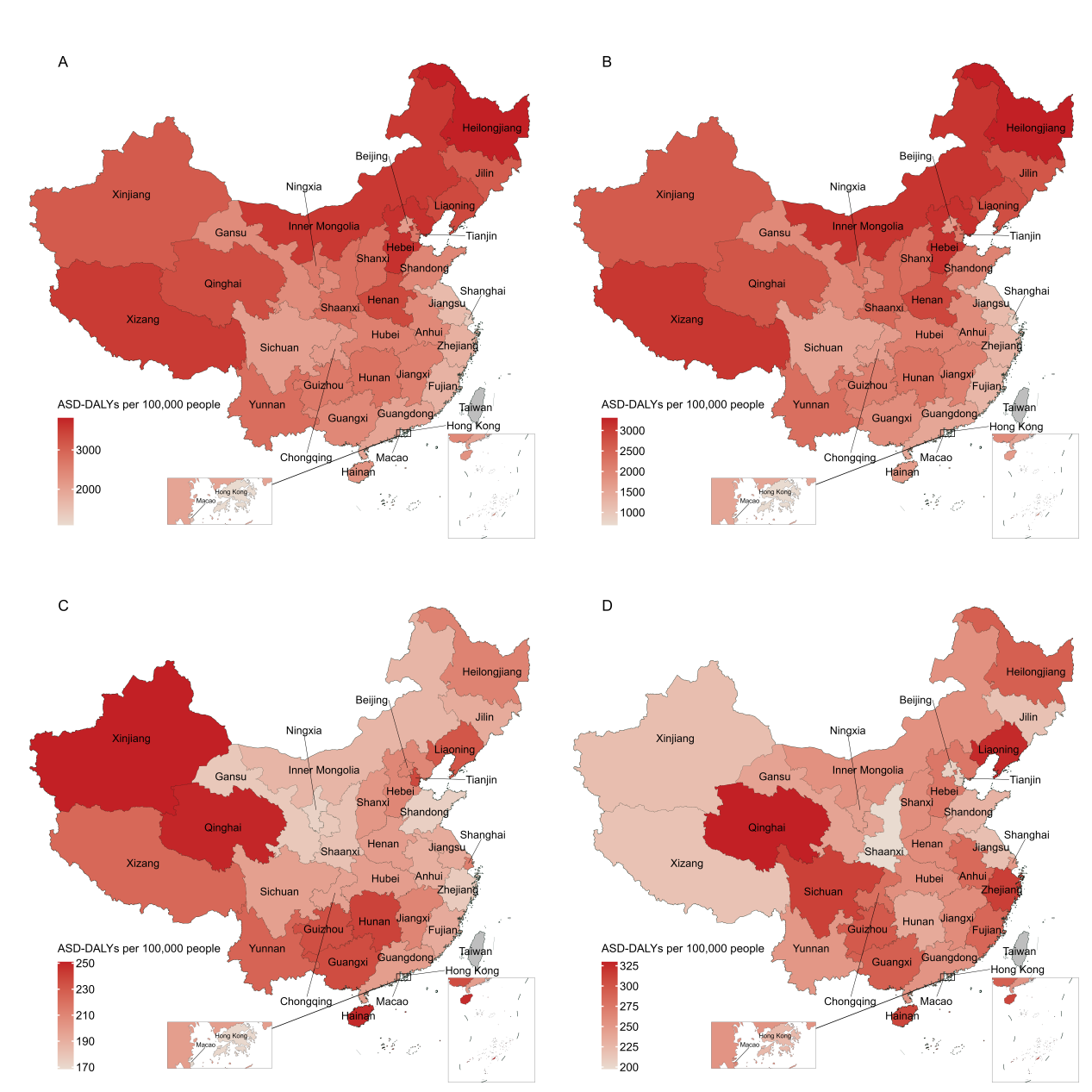

Supplement: Multimedia Appendix 2 [file publichealth-v11-e72978-s002.docx]
